# Enhancing (Super)hydrophobicity of Natural Fibers: An Overview of Methodologies and Their Sustainability Assessment

**DOI:** 10.1021/acssuschemeng.5c08518

**Published:** 2026-06-11

**Authors:** Petra Jerič, Blaž Likozar, Uroš Novak

**Affiliations:** † Jožef Stefan International Postgraduate School, Jamova 39, 1000 Ljubljana, Slovenia; ‡ Faculty of Mechanical Engineering, 112807 University of Maribor, Smetanova ulica 17, 2000 Maribor, Slovenia; § Department of Catalysis and Chemical Reaction Engineering, 68913 National Institute of Chemistry, Hajdrihova 19, 1000 Ljubljana, Slovenia

**Keywords:** hydrophobicity, sustainability assessment, natural fibers, PFAS-free coatings methodologies

## Abstract

Escalating regulatory pressures have accelerated the development of nature-based alternatives to per- and polyfluoroalkyl substances (PFAS) for hydrophobic textile coatings, driven by concerns over persistence and toxicity. Despite substantial progress, research remains fragmented, with diverse methodologies and limited comparative evaluation of environmental and industrial performance. This review systematically examines wet and dry approaches for applying PFAS-free, natural-based low-surface-energy materials and inducing surface roughness, with a focus on sustainability metrics including energy and water use, solvent consumption, time efficiency, scalability, versatility, waste generation, cost, performance, and durability. Each method is additionally evaluated through an integrated sustainability assessment combining environmental impact and industrial feasibility to identify the most practical and eco-friendly strategies. By highlighting critical bottlenecks and mapping opportunities in process design for biobased chemistries, this review provides a strategic roadmap to accelerate the understanding of the current state and to take an initial step toward upscaling and industrial adoption of PFAS-free alternatives and hydrophobic finishes for natural textiles. In addition, a frequency analysis of reported techniques from 2008 to 2024 reveals temporal trends in methodological development and highlights the dominant approaches and emerging technologies driving the transition toward more sustainable hydrophobic textile engineering.

## Graphical abstract

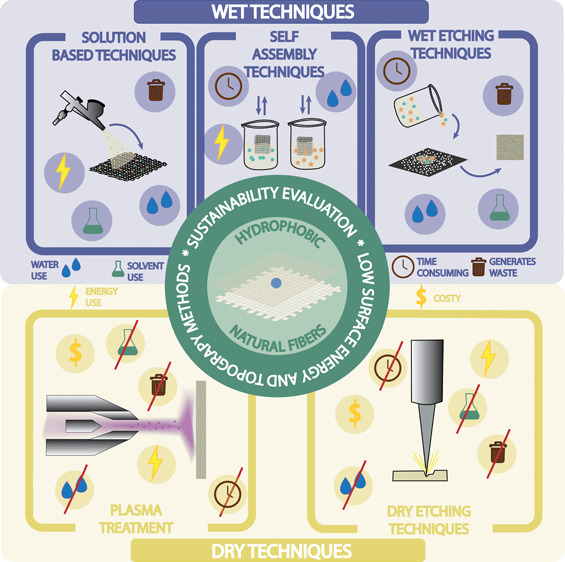

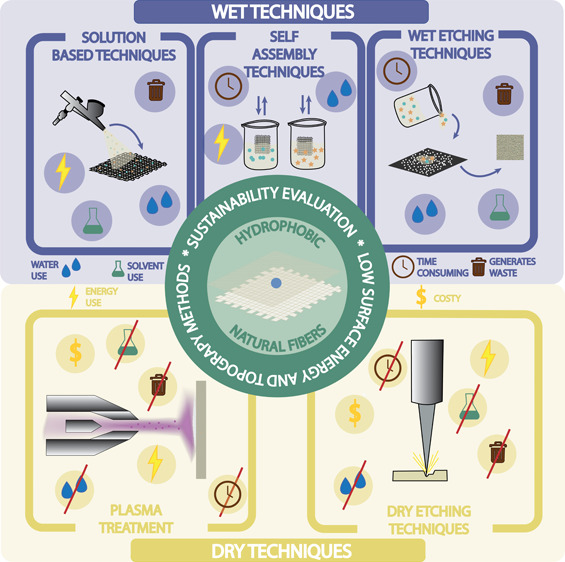

## 1 Introduction

The dual imperatives of performance and sustainability increasingly shape the development of durable, functional textiles. Natural fibers, including cotton, linen, jute, silk, and wool, are renewable and biodegradable, offering softness, breathability, comfort, and a lower environmental footprint than many synthetic polymers.^1,2^ Despite the dominance of synthetics, natural fibers still account for roughly 30% of global textile production,^3^ underscoring their enduring relevance in both daily life and industrial applications. However, their inherent hydrophilicity, due to the high content of hydroxyl and amine groups in cellulose- and protein-based structures, poses significant challenges to broader adoption. While this property facilitates moisture absorption and comfort, it also increases susceptibility to wetting, staining, and microbial colonization.^4^ Enhancing these materials with hydrophobic or superhydrophobic properties not only mitigates these drawbacks but also reduces bacterial adhesion and proliferation by lowering surface energy, ultimately extending textile lifespan and performance.^4^ However, when subjected to environmentally harmful modifications, natural fibers can lose their inherent biodegradability and can contribute significantly to fiber pollution, in some cases exceeding the impact of synthetic materials. For example, untreated cotton textiles may decompose within months under favorable conditions, whereas cotton fabrics treated with polyfluorinated waterproofing agents can persist for decades, with reported material half-lives extending up to 92 years.^5^

The growing recognition of the environmental persistence and toxicity of long-chain per- and polyfluoroalkyl substances (PFAS) has prompted extensive regulatory action worldwide, reflected in a series of national and international policy frameworks and restrictions.^6^ In the European Union the Registration, Evaluation, Authorization and Restriction of Chemicals (REACH)^7^ regulation banned C9–C14 per-fluoro-carboxylic acids (PFCAs) and PFHxA in consumer textiles. The REACH also includes PFAS in their catalog of substances of very high concern (SVHC). The European Commission’s July 2025 Action Plan calls for a comprehensive PFAS restriction extending to technical textiles and sealing applications.^8^ Further France (law no. 2025-188) banned the manufacture, import, export and market placement of PFAS-containing clothing, footwear and waterproofing agents effective January 2026; the ban widens to all textiles from 2030, with fines for noncompliance.^9^ Similarly, Denmark (Executive Order BEK no. 4649) banned clothing, footwear and waterproofing agents with total fluorine ≥ 50 mg F kg^–^ a grace period allows sale of existing stock until 1 January 2027.^9^ In the United States, oversight is implemented through the Toxic Substances Control Act (TSCA),^10^ the Environmental Protection Agency’s PFAS Action Plan,^11^ and a growing number of state-level bans targeting PFAS in consumer and industrial products. China classifies PFAS as “Highly Restricted Toxic Chemicals” and Japan regulates them under its Class I Specified Chemical Substances framework, limiting their use in textiles.^12^ In addition to these legal frameworks, industry initiatives such as the Zero Discharge of Hazardous Chemicals (ZDHC)^13^ program further promote safer chemical management and the elimination of hazardous substances from textile production. Manufacturing Restricted Substances List (MRSL) ensure the elimination of harmful inputs and control of effluents during manufacturing. Furthermore, voluntary certifications such as OEKO-TEX^14^ and Global Organic Textile Standard (GOTS)^15^ demonstrate chemical stewardship and strictly limit hazardous residues in the final consumer products. PFAS (including side-chain fluoridated polymers used for durable water repellents) are increasingly regulated due to their persistence, mobility, and bioaccumulation throughout the product life cycle (release during weathering/washing; microfibers; disposal). Releases of low-molecular-weight PFAS during the aging and washing of treated textiles have been documented, and older PFAS-finished textiles may pose greater exposure risks. Mounting regulatory pressures are driving both the textile industry’s shift to PFAS-free chemistries, such as silicones and alkylsilanes, and intensified research into safer, sustainable hydrophobization alternatives.^16,17^

Sustainability assessment of hydrophobic textile modifications requires a holistic framework integrating the environmental, social, and economic pillars of sustainability—*planet, people*, and *profit.* ^18^ Environmentally responsible approaches prioritize nontoxic, preferably biobased or renewable materials and green solvents while avoiding persistent and hazardous substances.^19^ However, sustainability extends beyond feedstock origin and must consider life-cycle resource efficiency and environmental impacts. Critical parameters include water and solvent use, chemical intensity, energy demand during drying and curing, process efficiency, and emissions to air, water, and solid waste streams.^18^ Coating durability is equally important, as prolonged hydrophobic performance reduces reapplication frequency and lifetime environmental burdens.^20^ Surface treatments should preserve fiber recyclability or biodegradability and prevent toxic residues during disposal or incineration. Environmental performance is therefore best evaluated through life-cycle-based assessments that capture impacts across production, use, and end-of-life stages while preventing burden shifting.^20–22^ Within such assessments, clearly defined system boundaries are essential, as they determine which processes and life-cycle stages are included. Common approaches include cradle-to-gate (raw material extraction to factory exit), cradle-to-grave (full life cycle to disposal), cradle-to-cradle (closed-loop material recovery), and gate-to-gate, which focuses solely on impacts within a defined production facility.^18,23^ Appropriate boundary selection is critical: overly narrow scopes may overlook significant impacts, whereas overly broad scopes can become impractical and data-intensive.^16^ The social dimension emphasizes worker and consumer safety, ethical sourcing, prevention of community exposure to hazardous chemicals, and transparency.^18^ The economic dimension addresses industrial feasibility, scalability, and market competitiveness while minimizing long-term costs, energy demand, and material consumption.^24^

The literature on PFAS-free hydrophobic coatings for natural textiles shows strong innovation, particularly in biobased chemistry and bioinspired structuring. However, despite high laboratory water repellency, practical performance is often insufficiently assessed, with durability testing frequently omitted and biodegradability rarely evaluated. Previous reviews are limited by their focus on PFAS-free formulations or method descriptions rather than systematic sustainability assessment, along with unclear system boundaries, a lack of comparable metrics, and minimal consideration of industrial feasibility. These gaps highlight the need for a systematic framework to evaluate both the environmental impact and industrial applicability of methods used for safer, natural-based PFAS-free hydrophobization.

This turtorial review aims to (1) systematically categorize hydrophobic modification methods used for regulation-driven fluorine-free coatings; (2) address a gap in the literature by evaluating their sustainability, especially through their environmental impact and industrial feasibility; (3) integrate qualitative assessment with comparative quantitative and semiquantitative analyses; (4) identify research trends and technological advances; (5) highlight key limitations, scalability challenges, and barriers to scalability to industrial scale; and (6) propose strategic directions for future research, material innovation, and the development of sustainable, and industry-ready hydrophobic textile coatings. An evaluation of sustainability using a multitiered approach is presented. First, a structured qualitative assessment based on gate-to-gate criteria covering simplicity, cost, energy demand, solvent and water use, time efficiency, scalability, versatility, waste generation, durability, and functionality was conducted. Key measurable environmental indicators at the gate-to-gate stage, including energy consumption, water use, solvent use, and waste generation, were further analyzed through quantitative and semiquantitative comparisons. Industrial feasibility was evaluated in terms of scalability and equipment requirements, time efficiency, process control, durability, and cost through semiquantitative assessment. By integrating environmental and economic dimensions into the final sustainability assessment and comparison, this framework identifies hydrophobic coating strategies suitable for sustainable industrial adoption. Toxicity and biodegradability are not assessed separately, as the focus is on natural-based, PFAS-free systems, which are assumed to have low toxicity and favorable biodegradability, primarily governed by material composition rather than processing methods, which are the main scope of this review.

Several assumptions and limitations affect the interpretation of the findings. Laboratory-scale data were used as proxies for sustainability comparisons, although pilot and industrial processes differ in scale, efficiency, energy integration, solvent recovery, and waste treatment; thus, results are indicative rather than representative of commercial practice. A gate-to-gate system boundary was applied due to limited life-cycle data and lack of comparable studies. Cross-study comparisons required harmonized assumptions on wet pick-up, liquor ratios, material flows, bath recovery, equipment losses, and process efficiency. Energy demand was estimated by assuming that drying and curing dominate the thermal loads, using simplified heat-balance calculations. Volatile solvents were assumed to fully evaporate, with residual bath fractions treated as effluent. A full life-cycle assessment in accordance with ISO 14040/44 ^25^ would be needed for definitive benchmarking. Industrial feasibility assessments may not fully capture economic, regulatory, and supply chain constraints. Despite these limitations, this review provides a comprehensive and actionable roadmap for advancing sustainable, PFAS-free hydrophobic modifications of natural textiles.

## 2 Theoretical Background

### 2.1 Hydrophobicity

Hydrophobicity is typically assessed by measuring the contact angle of a liquid on the surface. A water contact angle (WCA) greater than 90° is considered hydrophobic, while an angle greater than 150° is considered superhydrophobic^26^ as schematically represented in Figure 1a. Another essential criteria for superhydrophobicity is a low sliding angle (SA), also referred to as the tilt angle the minimum inclination of a solid surface at which a water droplet begins to slide off. In superhydrophobic fabrics, the SA is typically < 10.^27^ Achieving superhydrophobicity requires low surface energy and hierarchical nano/microscale roughness,^28^ a property offering benefits such as self-cleaning, anti-icing, antifogging, corrosion resistance, biofouling prevention, and friction reduction.^29^ Nature offers examples like the lotus leaf (Figure 1b), where water droplets roll off its wax-covered, micro/nanostructured surface, carrying away contaminants—a phenomenon known as the “lotus effect”.^30,31^

**Figure 1:**
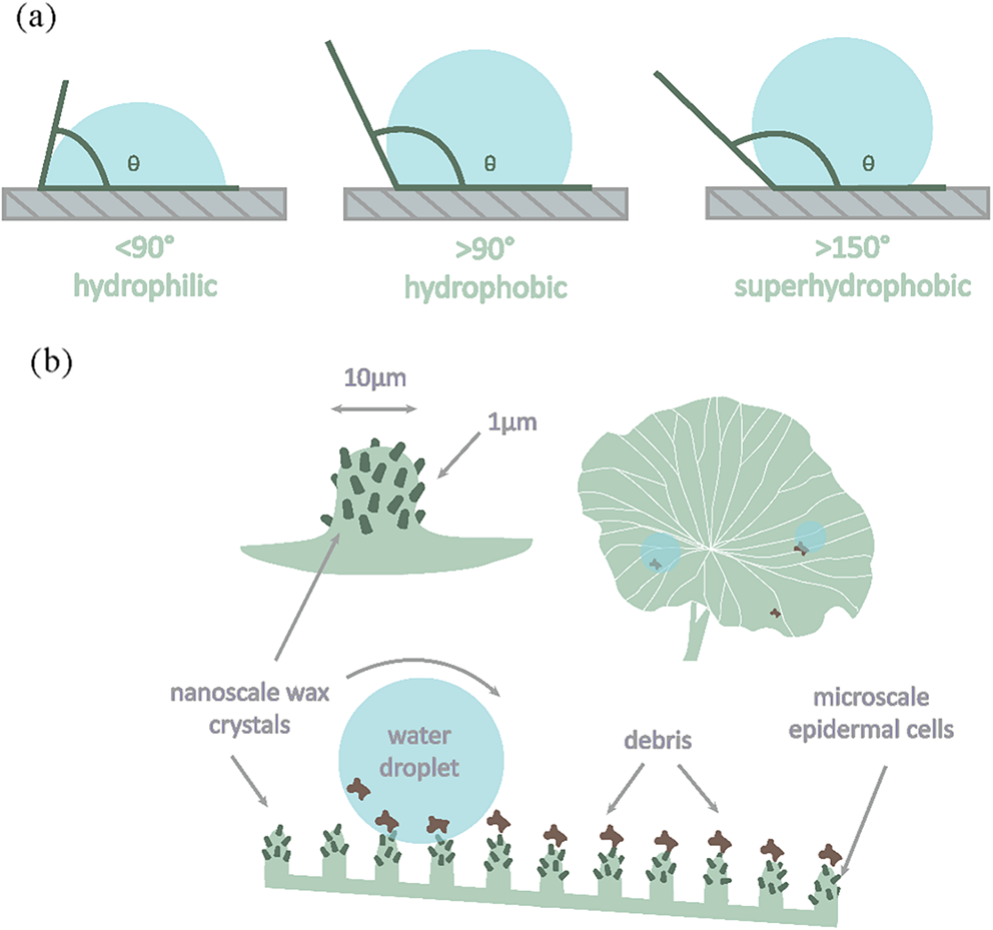
(a) Hydrophilic, hydrophobic and superhydrophobic contact angle, (b) superhydrophobic and self-cleaning lotus leaf effect.

### 2.2 Chemical Modification Processes Involved in Hydrophobization

The hydrophobization of natural textiles is a sophisticated process that relies on the synergistic function of both chemical and physical mechanisms. Physical connections, such as hydrogen bonding, van der Waals forces, mechanical entanglement, and capillary action, are crucial for ensuring the adhesion, durability, and functionality of the coated material. These noncovalent and mechanical interactions form the basis of physical modification strategies, which focus on altering the geometric structure and surface energy of the textile without necessarily creating new covalent bonds with the coating molecule (although often physical pretreatment facilitates subsequent chemical bonding).^32–34^ To improve chemical and physical connections, pretreatments that involve cleaning (e.g., scouring, desizing), cationization, and enzymatic treatment,^35,36^ plasma treatment, amination,^35^ or ultrasonic cleaning^37^ are performed to enhance fabric receptivity.^37^

Chemical modification is paramount because it involves the covalent attachment of functional groups to the abundant hydroxyl groups present in natural fibers.^4,38^ This chemical bonding is crucial for guaranteeing the durability and wash-fastness of the hydrophobic coating by preventing the functional groups from detaching during mechanical abrasion, laundering, or exposure to environmental stresses.^39–42^ Most common chemical reactions are shown in Figure 2.

**Figure 2:**
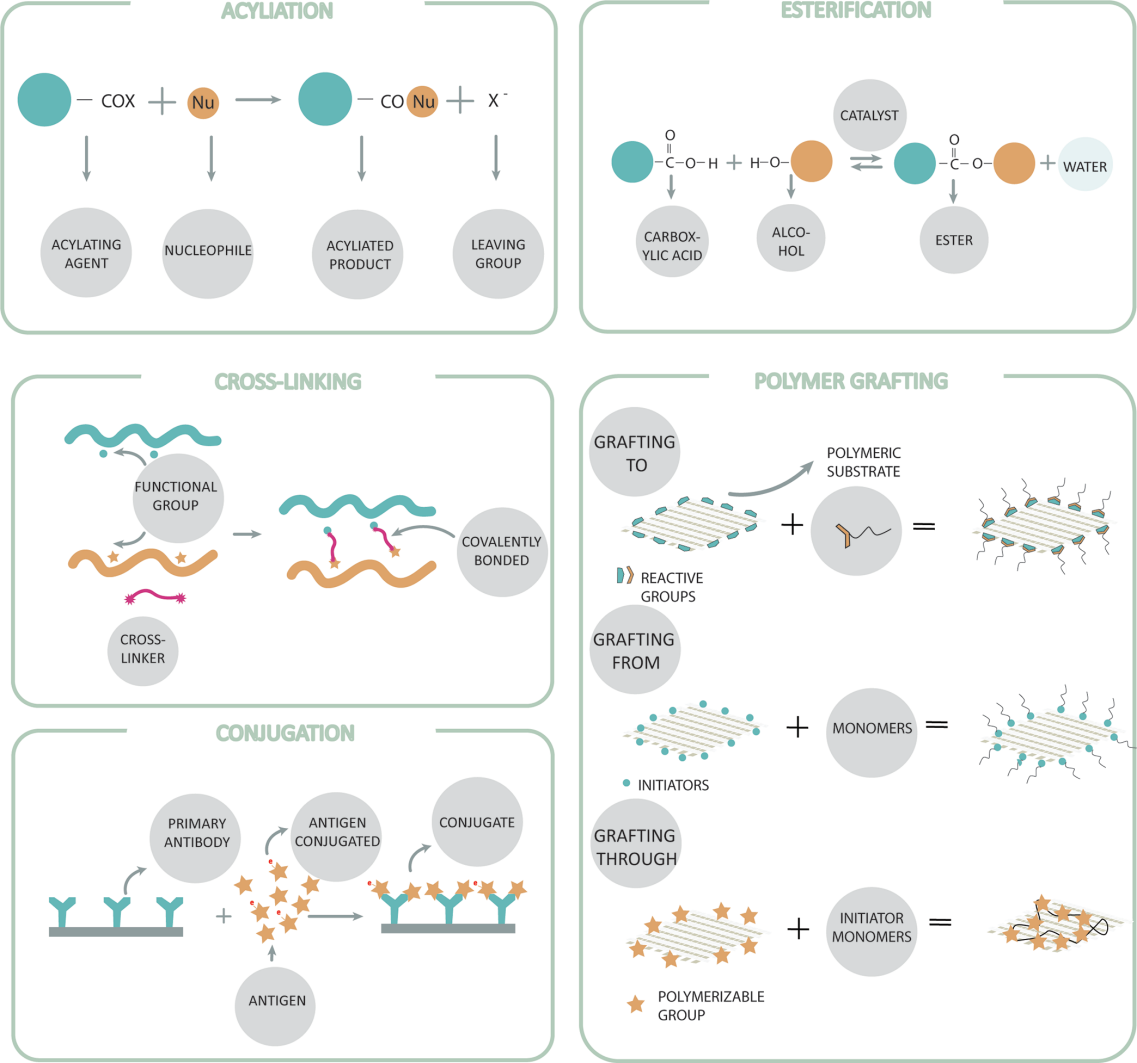
Most common chemical mechanisms: acylation, cross-linking, esterification, conjugation, polymerization, polymer grafting.

#### 2.2.1 Acylation

The reaction attaches acyl groups to substrates, targeting polar compounds.^43^ It shares key advantages of silylation, yielding less polar, more volatile derivatives, while acylation more effectively targets polar multifunctional compounds like carbohydrates and amino acids, producing fewer reactive byproducts. Acylation allows the conversion of compounds with active hydrogens, such as −OH, −SH, and −NH, into esters, thioesters, and amines, respectively.^43^

Depending on the compounds used, the reaction can be environmentally friendly. Liu et al.^44^ modified flax fibers through acylation using enzymatic lipase activity, conducted in the presence of a substantial excess of canola oil.

#### 2.2.2 Esterification

It is one of the most significant reactions in organic synthesis.^45^ The esters are found everywhere, both as natural and synthetic organic compounds, and are hydrophobic.^46^ Esterification is a chemical reaction in which an organic compound (typically an alcohol) reacts with an organic acid (carboxylic acid) to form an ester and water. The process involves the removal of a hydroxyl group (−OH) usually from the alcohol and a hydrogen atom from the carboxylic acid to form the ester linkage (RCOO-R’), with R and R’ representing organic groups. The primary objective of esterification should encompass the following criteria: precise control over the stoichiometric 1:1 ratio of carboxylic acid to alcohol, utilization of a neutral catalyst, elimination of the need for dehydration technology, and achieving both a complete conversion of reactants and a 100% yield.^45^ Mahmud et al.^47^ employed citric acid to modify the wetting properties of starch. In a study conducted by Huang et al.,^48^ esterification was carried out between catalyst and botulin.

#### 2.2.3 Polymer Grafting

It is a technique that involves attaching polymer chains onto a substrate to modify its properties. The substrate is first functionalized with reactive groups that can bond with the polymer chains. Once the substrate is functionalized, the hydrophobic polymer is grafted onto it via polymerization. The polymer chains attach to the functionalized groups on the substrate, forming a coating that renders the textile hydrophobic.^49^ Among the methods of modification of polymers, graft copolymerization offers an attractive and versatile means of imparting a variety of functional groups to a polymer through changing parameters such as the polymer types, the degree of polymerization, and the polydispersities of the main chain and the side chains, the graft density, and the distribution of the grafts.^49^ There are three general methods for synthesizing graft copolymers: “grafting to”, (prepolymerized chains are attached to reactive backbone polymers), “grafting from,” (conducting polymer backbone synthesized with initiation side functions as a macroinitiator from where side chains are grown) and “grafting through” (after polymerization, macromonomers are synthesized to form backbone polymer).^50^

Zhong et al.^39^ conducted an advanced grafting procedure, aiming to affix fatty acids with varying chain lengths onto cotton fabric. To achieve this, they utilized a mild accelerator—fatty anhydride. Enzymes play a vital role in green grafting reaction methods, enabling precise and environmentally sustainable chemical modification of polymers that is often not achievable with traditional synthetic approaches, as enzymes operate under exceptionally mild conditions. The enzyme acts fundamentally as a highly selective biocatalyst, facilitating the formation of covalent bonds between the textile substrate (the polymer backbone) and the functional molecule (the grafting agent) under mild conditions.^21^ Dong et al.^51^ performed laccase-mediated grafting of ODA onto lignin-rich jute fabrics, where laccase acts as a catalyst for long-chain amine to lignin phenolic sites. Similarly, Thakur et al.^52^ used laccase to graft ferulic acid onto coconut choir. Hossain et al.^53^ deposited lauryl gallate (C12 alkyl gallate) onto wool fabric catalyzed by laccase resulting in multifunctional fabric antioxidant, antibacterial, and water-repellent properties.

#### 2.2.4 Cross-Linking

It involves forming strong covalent bonds, or bridges, between polymer chains or molecules, resulting in a three-dimensional network or structure. Cellulose cross-linkers can be categorized into two primary types: those capable of self-polymerization and simultaneous cross-linking with cellulose, and those specifically engineered to cross-link cellulose, referred to as cellulose reactants. In the first scenario, three-dimensional polymers are generated by a process in which the cross-linkers self-polymerize and form a complex network of bonds with cellulose. Conversely, in the second approach, the cross-linking agents interact with the hydroxyl groups of cellulose, forming covalent bonds that enhance the material’s structural integrity. This dual classification provides a versatile framework for tailoring cellulose-based materials to meet a diverse range of applications.^54^ Replacing hydroxyl groups with hydrophobic moieties to improve stability, e.g., citric acid or epoxidized soybean oil (ESO) with stearic acid, is commonly used.^55,56^

#### 2.2.5 Conjugation

A conjugation reaction involves the joining or coupling of molecules or entities to create a larger, more complex structure. This can happen through various chemical processes, resulting in the formation of conjugated compounds or conjugates.^56,57^ Kim et al.^55^ used enzymatic conjugation of protein-flavonoids through laccase catalysis, culminating in the synthesis of biologically active polymers. The subsequent attachment of these conjugates to cationized flax fibers was efficiently achieved by leveraging ionic interactions between negatively charged proteins and the substrate, accomplished through incubation of the specimens.

### 2.3 Natural Compounds Used in the Hydrophobization of Natural Textiles

Natural materials are derived from abundant, renewable biomass (plants, animals, biowaste) and are biodegradable, reducing dependence on petroleum and minimizing persistent waste. They are often nontoxic.^58^ Sourcing materials from biowaste is crucial for addressing ethical and environmental concerns related to deforestation or competition for food resources.^19^ Herein, only a concise overview of selected nature-based approaches, as their comprehensive discussion and cooperative coating performance have already been included in our previous review article.^17^ Sustainable hydrophobization of textiles increasingly leverages natural, low–surface–energy compounds such as plant and animal waxes (carnauba,^59^ beeswax),^26^ natural fatty acids,^60–62^ and other natural hydrophobic compounds like betulin,^63^ tannins,^64^ rosin, and melanin. Natural polymers, especially polysaccharides, serve both as binders and, when used as NPS, also as generators of surface roughness. Naturally derived NPS, such as chitosan (CS), cellulose nanocrystals (CNC), and cellulose nanofibers (CNF), are critical because they can serve as a good alternative to replace metal NPS, which can be toxic if inhaled and are still commonly used in research. There are also studies using biomass and agricultural waste, such as eggshells,^65^ recycled cellulose from textile residues,^66^ and extracted CNF from peanut shell powder. In real-world applications, these natural compounds are often combined with some synthetic components, forming hybrid (natural-synthetic) systems.^19^ While natural-based compounds are increasingly favored due to their sustainability, abundance, and low toxicity,^19,58^ they frequently lack the robust mechanical durability required to withstand abrasive wear, repeated washing cycles, and prolonged chemical exposure.^19,67,68^ Consequently, synthetic components, such as silicates, acrylates, and poly­(dimethylsiloxane) (PDMS), are frequently integrated to act as binders, cross-linking agents, or supplementary low-surface-energy modifiers.^69,70^ These hybrid coatings leverage the environmentally friendly nature of natural-based substances, while the synthetic additives, through mechanisms like cross-linking, provide the necessary chemical adhesion and mechanical stability to the coating layer. Natural waxes often exhibit poorer mechanical and thermal stability when used alone, necessitating their combination with polymers or NPS to achieve adequate durability.^19^ This strategic integration is crucial for achieving high-performance criteria, ensuring that nature-inspired solutions can effectively match or exceed the efficiency and durability standards previously set by environmentally hazardous substances like per- and polyfluoroalkyl substances (PFAS). In Table 1, we gathered the most commonly used natural compounds for hydrophobic coatings of natural textiles.

**Table 1:** Most Commonly Used Natural Compounds in Hydrophobic Coatings for Natural Textiles, Their Role in Hydrophobic Coatings, Source, Environmental Impact, Their Functional Groups, and Examples of Their Hydrophobic Performance

Natural compounds and their source	Environmental impact	Functional group and role in the hydrophobic coating	Hydrophobic performance
***Carnauba wax***	Renewable, biodegradable, nontoxic, edible, abundant. If harvesting is done improperly or excessively, it could potentially harm the long-term viability of the individual palms and the overall stand, thereby increasing the risk of deforestation. The extraction of wax powder from the dried leaves generates large volumes of fibrous waste (straw).^71,72^	Esters (−COO−), hydroxyl group (−OH), hydrocarbons.	WCA (cotton, carnauba wax, poly-l-lysine) = 155 (±15)°, SA (cotton, carnauba wax + poly-l-lysine) = 9°^59^
Leaves of carnauba palm (*Copernicia prunifera*).^72^		Low surface energy material.^59^	
***Beeswax***	Renewable, biodegradable, has higher greenhouse emissions compared to plant-based waxes due to the energy required for beekeeping operations (0.44 to 3.18 kg CO_2_ e/kg).^73^	Esters (−COO−), carboxyl group (−COOH), hydroxyl group (−OH), alkane/alkyl chains (CH_2_ in CH_3_), ketone/aldehyde groups (C=O in ketones/aldehydes).	WCA (taro root, TiO_2_, beeswax) = 128 (±2)°^26^
Honeybees, primarily *Apis mellifera.* ^26^		Low surface energy material^26^	
***Stearic acid (STA)***	Renewable, biodegradable, tallow-based production can achieve lower primary energy use and a negative cradle-to-gate green warming potential (GWP) under favorable allocation assumptions. Vegetable oil routes generally exhibit higher GWP due to land-use change.^74^	Carboxylic group (−COOH), long saturated alkyl chain −CH_2_ (methylene), and −CH_3_ (methyl).^75^	WCA (STA) = 110 (±10)°^76^
Plant and animal fats (tallow, beef fat), and oils (palms, soybean, canola, coconut oils).^19^		Low surface energy material^76^	
***Oleic acid***	Renewable, biodegradable, low-toxicity for human health, moderate acute toxicity to *Daphnia* (EC_50_ ≈ 0.3 mg L^–1^), indicating that high concentrations can affect freshwater invertebrates. Derived oleic acid emits approximately 1.5–3.5 kg CO_2_ e/kg; tallow-derived oleic acid emits slightly higher due to methane emissions from livestock.^77^	Carboxylic group (COOH), carbon–carbon double bond (C=C), long hydrocarbon chain.	WCA (oleic acid, argon plasma) > 150°^60^
Plant and animal fats and oils (mango, avocado, olive oil, and canola oil).^77^		Low surface energy material.^60^	
***Cellulose nano crystals (CNC)***	Renewable, biodegradable, nontoxic, greenhouse gases emissions for production show wide variations, ranging from 1.8 to 1100 kg CO_2_.^78^	Hydroxyl groups (−OH), hemiacetal groups.^79^	WCA(CNC, CS) = 135°^80^
Wood pulp,^40^ agricultural residues,^80^ plant fibers.^79^		Nano/micro surface roughness.	
***Cellulose nano fibers (CNF)***	Renewable, biodegradable, nontoxic, producing CNF typically requires high energy input for homogenization/refining,^79^ high current costs, limited capacity of pilot- and early stage production, GHG emissions for production show wide variations, ranging from 1.8 to 1100 kg CO_2_.^79^	Hydroxyl groups (−OH), carbonyl groups (C=O).	WCA (CNF, octadecylamine-ODA) = 152(±3)°^40^
Wood pulp, agricultural residues, plant fibers.^79^		Nano/micro surface roughness.^40^	
***Chitosan***	Renewable, abundant, made from byproducts, biodegradable, and low-persistence at the end of life. The extraction of chitin from shells and its subsequent deacetylation into CS often relies on harsh chemical processes, which can generate problematic effluents.^81^	Hydroxyl (−OH), protonated amino (−NH) groups.	WCA (CS, TiO_2_) = 16°^82^
Crustaceous shells^82^		Adhesion, antimicrobial role, nano/micro surface roughness (if in the form of NPS).^82^	
***Lignin***	Abundant, renewable, and biodegradable, but with disposal issues.^83^	Hydroxyl groups (−OH), methoxyl (OCH_3_), carbonyl (−C=O), and carboxyl (−COOH) groups.	WCA (beeswax, lignin, *n*-hexane) = 150°^84^
Agricultural and forestry^83^ wood residues, energy crops, flax and jute fiber, peanut shells.^85^		Hydrophobic building block,^83^ adhesion, surface roughness.	
***Natural rubber***	Renewable resource, biodegradable, lower carbon footprint, but risks deforestation and loss of biodiversity.^62,86^	Carbon–carbon double bonds (C=C), hydroxy (−OH) groups, carboxyl (−COOH) groups, amine/amide (−NH_2_/-NH-C=O).	WCA (NRL, AuNP) = 132° ^62,86^
Sap of rubber trees, mainly *Hevea brasiliensis.* ^17^		Low surface energy material, surface roughness (if in the form of NPS), film forming capability.^87^	
***Zein NPS***	Renewable, biodegradable (with long-term stability in the environment), lower inherent toxicity, and sensitive to pH/ionic strength.^88^	Amide I (C=O), amide II (CH stretching, N–H bending, amino acid side, hydrophobic side chains, hydroxyl (−OH), carboxyl group (−COOH).	WCA (zein NPS, ethanol) > 90°^88^
Corn (maize)^88^		Nano/micro surface roughness, antimicrobial.^88^	
***Betulin***	Renewable and biodegradable, the sustainability of betulin extraction depends on the solvent used. Liquid CO_2_ extraction (50 bar, 16 °C) with 20 wt % ethanol as a cosolvent results in a lower environmental impact.^89^ Large-scale harvesting of birch bark could impact forest ecosystems if bark removal exceeds sustainable limits; however, the reviewed studies focus on valorizing existing industrial bark residues rather than new harvesting, thereby minimizing habitat disturbance.^90^	Hydroxyl group (OH), isopropenyl group C=CH_2_, hydrocarbon moiety C_30_H_50_.	WCA (betulin, ethanol) = 123°^63^
The outer bark of the brich trees (*Batula species*).		Low surface energy material.^63^	
***Rosin***	Renewable, abundant, biodegradable^91^	Carboxyl group (−COOH) Alkene (C=C)	WCA (zein, rosin) = 133(±3)°^91^
Pine trees^91^		Low surface energy material.^91^	
***Phytic acid (PA)***	Renewable, low-toxicity, and green manufacturing processes can be used to produce food sources.^84^	Phosphate groups P–O(OH)_2_	WCA (PA)=83°^92^
Cereal, grains, legumes, oil seeds, nuts, fruits, vegetables, pollens, and spores.		Surface roughness via etching.^92^	
***Tannin***	Biodegradable, renewable, abundant. The extraction of tannin is energy- and water-intensive. High concentrations of tannin in effluents can harm marine organisms.^93^	Phenolic hydroxyl (−OH) groups, aromatic rings.	WCA (tannin, CNC, ODA)=158°^94^
Barks, leaves, seeds, stems, fruits.		Cross-linking agent, surface modifier (if the form of NPS), UV, and antioxidant protection.^95^	
***Melanin NPS***	Renewable, nontoxic, biodegradable.^69^	Catechol Groups (dihydroxyphenolic groups), carboxy group (−COOH).	WCA (melanin, PMDS) = 164 (±4)°
Yak hair, alpaca hair, cuttlefish ink.^69^		Micro/nano surface roughness.	
***Laccase***	Biodegradable, renewable, sustainability depends on robust enzyme sourcing and formats (e.g., immobilized laccase), enzyme costs, and operational stability.^96,96^	Copper-binding motifs (Cu+) amino-acid (CH(NH_2_)-COO) side chains.	WCA(laccase, ODA) = 117°^44^
Plants (e.g., lacquer tree sap), fungi (especially white-rot *basidiomycetes*), bacteria, insects, and even some archaea.		Catalyst.	
***Lipase***	Renewable, biodegradable, enzyme costs, and operational stability.^97^	Hydroxyl (−OH group), Imidazole (Im) group, carboxyl (−COOH) group.	Water uptake (lipase, canola oil) = 1.7% in 500 min
Bacteria (e.g., *B. subtilis*), fungi, yeast, animal pancreas, and plant tissues.^97^		Catalyst.	

## 3 Methodological Framework

### 3.1 Literature Selection

Out of more than 100 reviewed studies on PFAS-free hydrophobic modifications, 49 were selected for detailed analysis. To better evaluate the sustainability of natural-based coating processes, only research papers reporting at least one naturally derived compound in their formulation were included. Completely natural-based coatings are, unfortunately, almost nonexistent; therefore, this inclusion criterion allowed us to focus on systems that incorporate a natural component while still reflecting the realistic landscape of current research. Studies featuring this type of content, conducted between 2008 and 2024, were retrieved from the Google Scholar database. The year 2008 was chosen because it marks the publication of the earliest article on nature-based hydrophobic coatings for textiles that we were able to find. The hydrophobization methods used in these articles were categorized into subcategories and described in this review article.

### 3.2 Method Classification and Frequency Analysis

Frequency analysis was performed by counting all deposition and treatment methods reported across the reviewed studies. When multiple techniques were employed within a single hydrophobic modification, each method was included in the count. Trends were then identified and analyzed across the entire data set, as well as separately for studies published in the past three years (2022–2024), to evaluate which approaches are most frequently used for achieving PFAS-free hydrophobization of natural textiles recently.

### 3.3 Sustainability Assessment Framework

#### 3.3.1 The Qualitative Sustainability Assessment

A descriptive sustainability assessment was performed for all reviewed hydrophobic methods using gate-to-gate criteria, including simplicity, cost, energy demand, solvent and water use, time efficiency, scalability, versatility, waste generation, performance, and durability. The evaluation was based on data extracted from primary research articles and review literature describing these approaches. Sustainability assessments are provided following the description of each method.

#### 3.3.2 The Quantitative Assessment of Environmental Impact

To enable comparative gate-to-gate analysis, a quantitative environmental impact assessment was conducted for four key indicators: energy use, water consumption, solvent consumption, and waste generation, normalized to a functional unit of 1 m^2^ of fabric (200 g m^–2^). These criteria were selected because they capture the primary process-level environmental burdens in textile finishing and could be consistently derived from the available data. Energy use strongly influences greenhouse gas emissions and operational costs, making it critical for both environmental and economic sustainability. Water consumption is highly significant due to its implications for resource scarcity, effluent generation, and wastewater treatment demand. Solvent consumption carries substantial weight because volatile organic compounds contribute to air emissions and occupational exposure risks. Waste generation reflects material efficiency and the environmental load associated with effluents and byproducts.

Information for the assessment was compiled from primary research articles and review studies. Because the data set is limited to laboratory-scale experiments, the results should be interpreted as indicative. A comprehensive cradle-to-gate life cycle assessment at pilot or commercial scale would be required for definitive benchmarking, but was beyond the scope of this review. Process steps for each method—including application, drying, and curing—were mapped, and liquor ratios were determined based on reported wet pick-up values.

Energy use was estimated through a simplified heat balance around the drying and curing stages, which represent the dominant energy consumers in textile finishing. The total energy demand was calculated as the sum of (I) the latent heat required to evaporate the measured water mass (L ≈ 2.26 MJ/kg of water), (II) the sensible heat for warming the fabric and air to the processing temperature, and (III) estimated oven and stenter losses, derived from reported power consumption and residence time data. For dry finishing methods (e.g., plasma or UV curing), energy demand was computed from electrical power and line speed, yielding energy per meter of treated fabric.

Water consumption was calculated based on the reported liquor ratio and the number of wet processing steps involved in each method (e.g., padding, rinsing, washing). The liquor ratio, defined as the volume of treatment bath per mass of fabric (L/kg), was multiplied by the fabric mass throughput per square meter (200 g/m^2^ basis) and by the number of wet steps to estimate the total process water use. This approach accounts for variations in wet pick-up and bath reuse across different studies.

Solvent consumption was estimated based on the wet pick-up of the treated fabric, which represents the mass fraction of coating solution absorbed relative to the dry fabric weight. From this, the total applied solution mass per square meter of fabric was calculated. The solids content of the coating formulation, obtained from literature or reported experimental data, was then used to separate the solvent fraction from the total applied solution. The solvent mass was therefore computed as the product of the total solution mass and the solvent weight fraction (1 – solids content). This approach accounts for formulation concentration and wet pick-up variability across different studies.

Waste and emissions were estimated using a mass balance approach on the applied chemicals. Unexhausted fractions of the treatment baths, determined from reported uptake or exhaustion efficiencies, were considered to contribute to the effluent load. Volatile components and solvents released during drying and curing were treated as VOC emissions, with the volatile fraction estimated by assuming complete evaporation of low-boiling components. Assumptions regarding bath recovery and complete evaporation were applied uniformly to ensure comparability across studies.

#### 3.3.3 The Semiquantitative Assessment of Environmental Impact

The semiquantitative environmental impact evaluation was based on results from the quantitative analysis. The overall environmental impact score for each method was determined using a semiquantitative normalization approach based on four gate-to gate indicators: energy use, water consumption, solvent consumption, and waste generation. For each indicator, the highest average value among all methods was set as the 100% reference. Relative values were then scored as −1 (60–100% of the maximum), 0 (10–60%), or +1 (<10%). Indicator scores were summed to yield a final environmental impact score ranging from −4 to +4, with all indicators weighted equally. Environmental impact was classified according to the final score: +4 (very low environmental impact; highly favorable), +3 (low impact), +2 (moderate impact), +1 (slightly elevated impact), 0 (moderate environmental impact), −1 (elevated impact), −2 (high impact), −3 (very high impact), and −4 (extremely high impact; environmentally burdensome).

#### 3.3.4 The Semiquantitative Industrial Feasibility Assessment

Criteria, including scalability and equipment, time-efficiency, process control, performance, durability, costs were incorporated in a complementary, semiquantitative framework to capture industrial feasibility and functional performance, which are not reflected by quantitative environmental impact metrics. Qualitative evidence from the literature was translated into numerical scores based on predefined criteria representing practical implementation conditions, with equal weighting applied across assessment types to maintain neutrality. Where available, quantitative data were included; for example, time efficiency was calculated based on reported durations of each process step in the literature. Industrial feasibility was scored on a scale from −4 to 4, where 4 indicates very high feasibility or widespread industrial use, 3 denotes high feasibility with minor limitations, 2 reflects moderate feasibility with potential for optimization, 1 represents limited but possible implementation, 0 neither feasible nor infeasible, −1 corresponds to difficult implementation at industrial scale, −2 denotes very difficult implementation, −3 indicates extremely challenging or mostly lab-scale methods, and −4 reflects methods that are not industrially feasible. Additionally, the methods were grouped into 3 main classes depending on industrial maturity: established, emerging, and lab-scale.

#### 3.3.5 The Overall Sustainability Assessment

It integrates the semiquantitative environmental impact evaluation and the industrial feasibility assessment into a single metric, providing a balanced perspective on both environmental performance and practical implementability. The score is calculated as

Overall Sustainability Score=0.5×Environmental Impact Score+0.5×Industrial Feasibility Score

We assume equal weighting for transparency, but note that alternative weightings could be applied depending on stakeholder priorities. Toxicity and end-of-life considerations are not included into assessment, as all formulations are assumed to be natural-based, nontoxic, and biodegradable. This framework is intended to assess the sustainability of the methods themselves, enabling comparison of their relative environmental and operational performance rather than the inherent properties of the formulations. Scores range from −4 to +4, with higher values indicating greater sustainability. Methods scoring +4 are considered very highly sustainable, combining minimal environmental burden with excellent industrial feasibility. Scores of +3 denote high sustainability, reflecting low environmental impact and generally feasible implementation with minor limitations. A score of +2 indicates moderate sustainability, with moderate environmental impact and potential for process optimization. Scores of +1 reflect slightly favorable sustainability, while 0 denotes neutral sustainability, often due to trade-offs. Negative scores indicate lower sustainability, with −1 and −2 corresponding to elevated or high environmental impact and limited feasibility, −3 representing very low sustainability, typically for methods that are mostly lab-scale or environmentally burdensome, and −4 reflecting extremely low sustainability, combining high environmental burden with poor industrial feasibility.

## 4 Methods Employed to Enhance the Hydrophobicity of Natural Textiles

### 4.1 Wet Methods

#### 4.1.1 Solution-Based Deposition Techniques

These conventional coating techniques are commonly employed to deposit low surface energy materials or NPS, thereby imparting hydrophobicity through surface chemistry modification and increased surface roughness.^98,99^

##### 4.1.1.1 Dip Coating

The dip-coating method (Figure 3, Table 2) is widely used for modifying the surfaces of textiles. The process can be broken down into four primary stages, which can be performed discontinuously (in batches) or as part of a continuous production line.^33,100^ The first step is immersion, where the textile substrate is submerged in a tank containing the coating solution.^99,101,102^ In the second stage -dwelling, the substrate may remain in the solution for a set period to ensure complete wetting and infiltration of the coating material into the porous structure of the textile.^99,101,102^ In the withdrawal stage, the textile is withdrawn from the solution at a constant, controlled speed. A thin liquid film adheres to the substrate surface as it is pulled out.^58,99,103^ Excess liquid drains off. The film solidifies as the solvent evaporates. This stage is usually accelerated by heat (e.g., in an oven with hot air or infrared heating), which also helps to cure the coating, permanently fixing it to the textile fibers. This dip-and-dry cycle can be repeated multiple times to create multilayered coatings, which can increase the thickness and uniformity of the film or build complex functional layers by using different solutions in successive steps.^100^ As the textile is withdrawn, it entrains a layer of the liquid, which is influenced by a competition between viscous drag (pulling the liquid up), gravity (pulling it down), and the surface tension of the liquid (capillary forces).^103^ The final film thickness is primarily governed by two regimes related to withdrawal speed. In the draining regime (high speeds), thickness is controlled by the balance between viscous drag and gravity, with faster withdrawal yielding thicker coatings. In the capillary regime (low speeds), solvent evaporation dominates, and capillary action draws additional solutions to the drying line, increasing the thickness as the speed decreases. Key factors influencing film thickness and morphology include withdrawal speed, solution properties (viscosity, density, surface tension, concentration), environmental conditions (temperature, humidity), and substrate characteristics, as surface roughness and energy affect wetting and adhesion.^33,99,100,104^ A dip-coating system consists of a motor-driven, programmable linear stage (often stepper-motor based) that controls immersion and withdrawal speeds, a substrate holder or clamp, a solution reservoir (dip tank) for the coating bath, optional temperature control for the bath, and a drying/curing unit (heated air, infrared or oven) to evaporate the solvent and solidify the film.^105^

**Figure 3:**
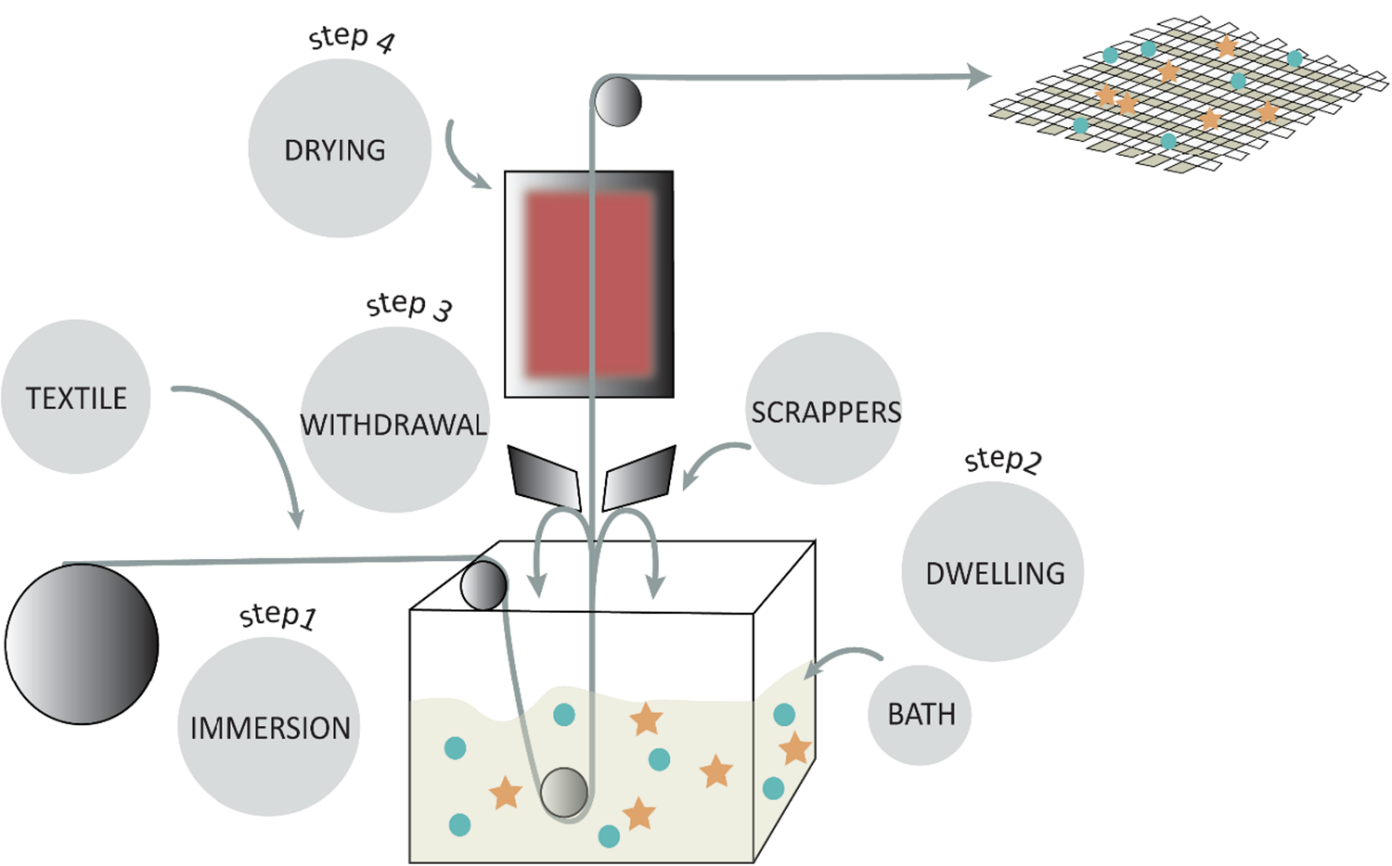
Schematic presentation of dip-coating: immersion of textile in the solution, dwelling, withdrawal, scrapping of access solution and drying.

**Table 2:** Natural-Based Hydrophobic Coatings Using the Dip Coating Technique: Used Methods and Reaction Mechanism, Textile Substrate and Coating Materials, Process Steps, and Treatment Conditions, Hydrophobic Performance and Durability

Methods used and reaction mechanisms	Textile substrate and coating materials	Process steps and treatment conditions	Hydrophobic performance and durability	ref
dip-coating	100% cellulosic fibers	1.) Cleaning & drying of the leaves (80 °C, 4 h),	WCA (taro root, TiO_2_ beeswax) = 128 (±2)°, WCA (taro root) = 96 (±9)°, WCA (taro root, TiO_2_) = 104 (±2)°, WCA (taro root, beeswax) = 112 (±6)°	^26^
		2.) preparation of coating material,		
esterification, van der Waals forces	taro-root leaves extract, TiO_2_ NPS, beeswax	3.) dipping and air drying.		
dip-coating	100% cellulosic fibers	1.) Cleaning of material (30 min with ethanol),	WCA (CESO treatment) = 115°, WCA (CESO+CNC treatment) = 143°, WCA (CESO+CNC+HDTS) = 157°	^98,99^
		2.) immersion in CESO for 0.5 h, curing: 150 °C for 0.5 h,		
esterification, grafting	CNC, (cured epoxidized soybean oil) CESO, hexadecyltrichlorosilane (HDTS)	3.) immersion in CNC for 1 h, curing at 150 °C, 2.5 h,		
		4.) immersion in HDTS for 6 h.		
dip-coating	100% cellulosic fibers	1.) CS-Tio_2_ solution preparation,	WCA (CS) = 62°, WCA (CS, 1 wt % TiO_2_) = 75°, (CS, 3 wt % TiO_2_) = 123°, SA (CS, 3 wt % TiO_2_) = 60°, WCA (CS, 5 wt % TiO_2_) = 140°, SA (CS, 5 wt % TiO_2_) = 25°, WCA (CS, 7 wt % TiO_2_) = 161°, SA (CS, 7 wt % TiO_2_) = 5°	^82^
physical interactions	CS, TiO_2_	2.) immersion for 30 min,		
		3.) squeezing and drying (pressure 2 kg/cm^2^), dried at room temperature.		
dip-coating	100% cellulosic fibers	1.) Preparation of the coating solution,	WCA = 160°, SA 7°	^111^
cross-linking, grafting	CS, amino carbon nanotubes (ACNTs), ODA)	2.) immersion for 10 min, air-dried for 12 h.		
dip-coating	100% cellulosic fibers	1.) Pretreatment via Soxhlet extraction for 8 h,	WCA > 90°, drop penetration time = 50 min	^112^
physical interactions	hydrophobins	2.) immersion for 30 min,		
		3.) drying 20 min at 100 °C.		
dip-coating	100% cellulosic fibers	1.) Extraction of betulin,	WCA = 136°	^48^
oxidation, esterification	betulin, toluene	2.) immersion single air-dry overnight.		
dip-coating	100% cellulosic fibers	1.) One step immersion, stirred on 50 °C, 3 h,	WCA = 158°	^113^
polymerization, cross-linking	caffeic acid, FeSO_4_, laccase	2.) drying in an oven, 60 °C.		
dip-coating	cellulosic fibers	1.) Preparation of coating,	WCA = 159°	^42^
reduction (of copper acetate), physical interactions	STA, copper	2.) immersion for 12 h at room temperature,		
		3.) rinsing (distilled water, ethanol),		
		4.) air-dried for 4 h.		
dip-coating physical interactions	cellulosic fibers	1.) Prewashing of cotton,	WCA = 165 (±1)°	^114^
physical interactions	STA, TiO_2_, PDM	2.) synthesis of TiO_2_ NPS,		
		3.) STA; functionalization of TiO_2_,		
		4.) immersion for 0.5 min, withdrawn at a constant speed of 10 cm s^–1^, squeezed,		
		5.) air-dried at 15 min at ambient temperature,		
		6.) curing in an oven at 150 °C for 5 min.		
dip coating	cellulosic fibers	1.) Preparation of tannic-acid self-polymer,	WCA = 139 (±2)°	^115^
		2.) immersion in PTA–PTA-CS at 50 °C at water bath for 48 h,		
self-polymerization, cross-linking, phase separation	CS, tannic acid (TA)	3.) drying at 60 °C,		
		4.) immersion in PTA-CS for 1h at 50 °C,		
		5.) dried at 105 °C for 3h,		
		6.) immersion in PDMS solution, fully oscillated for 2.5 min,		
		7.) drying at 105 °C for 5 h.		
dip-coating	cellulosic fibers	1.) Pretreatment (scoring) at 80 °C for 30 min, dried at 120 °C for 30 min,	WCA = 150°	^116^
dip-pad-dry silanization	CS, PDMS	2.) creating nanoscale roughness: immersion in CS coating,		
		3.) wet textile exposed to ammonia gas, causing the CS to precipitate from the acidic solution and form nanostructured roughness,		
		4.) drying at 80 °C, 5 min,		
		5.) lowering surface energy, immersion for 1 min,		
		6.) drying in preheated oven 80 °C for 5 min and curing at 150 °C for 1 min,		
		7.) rinsing with water several times,		
		8.) drying in preheated oven 80 °C for 5 min and curing at 150 °C for 1 min.		
pretreatment, dip-coating	cellulosic fibers	1.) Cotton pretreatment,	WCA = 149°	^117^
cross-linking polymerization, physical interactions (hydrogen bonding, electrostatic interactions)	PA, titanium oxide, poly(ethylenimine) ethanol	2.) immersion in poly(ethylenimine) solution for 10 min, 25 °C, washed with water, dried at 70 °C to unchanged weigh,	WCA (after 30 abrasion cycles) = 143°, WCA (after 50 abrasion cycles) = 153°, WCA (immersion for 7 2 h in organic solvents) > 138°	
		3.) immersion in TiO_2_ solution for 30 min at 25 °C, curing at 105 °C for 5 min, washed, dried at 70 °C,		
		4.) immersion in urushiol ethanol solution for 5 h at 25 °C, curing in an oven at 120 °C for 2 h.		
scouring pretreatment, nanoseeding/nanorod treatment, dip coating	100% jute fabric	1.) Pretreatment (scouring)	WCA (ZnO, STA) = 148 (±9)°, WCA (ZnO) = 55 (±10)°, WCA (STA) = 110 (±10)°, WCA (AATCC Test Method 61–1996) < 150°,	^76^
hydrolysis (pretreatment), grafting	ZnO NPS, STA, ethanol	2.) ZnO nanoseeding: immersion in NaOH, ZnAc, ethanol solution, 4 times for 115 min, drying at 120 °C for 15 min		
		3.) ZnO nanostructure growth: immersion in zinc nitrate hexahydrate (ZnNi) and hexamethylenetetramine (HMTA) for 5 h at 95 °C, rinsing in distilled water, dried at room T,		
		4.) immersion in STA for 3h, dried for 15 min at 100 °C.		
in situ synthesis, dip-coating	100% cellulosic fibers	1.) Preparation of hybrid colloid (heating on 90 °C for 40 min),	WCA (7 coating cycles) = 132°, WCA (8, 9 coating cycles) = 132°	^62^
physical interactions	natural rubber NPS, gold NPS	2.) immersion in a colloid suspension of natural rubber and gold NPS, drying in an oven at 100 °C.		
chemical reduction method (silver NPS), dip-coating	100% cellulosic fibers	1.) Cleaning and oven drying of cotton,	WCA (NRL) = 97°, WCA (NRL: Ag NPs = 1:1) = 80°, WCA (NRL: Ag NPs = 1:3) = 65°, WCA (NRL) = 102°	^87^
esterification, reduction, physical (van der Waals Forces, hydrogen bonding)	natural rubber latex, CS-*graft*-poly(acrylamide), silver NPS	2.) immersion in NRL/silver NPS, squeezed with roller (30% compression), air-drying at room temperature for 10 min,		
		3.) UV-curing with 365 nm UV. lamp for 3 min on each side of fabric,		
		4.) washing three times in deionized water on 30 °C for 5 min,		
		5.) drying in oven at 60 °C for 30 min.		
dip coating	100% cellulosic fibers	1.) Preparation of cotton (washing in NaOH, detergent),	WCA (CS+PaNi+ZnO+STA) = 154°, SA = 5°, WCA (CS+ PaNi+ZnO) = 90°, WCA (CS+PaNi) = 104°	^2^
in situ-polymerization	CS, polyaniline (PAni), ZnO, STA	2.) immersion in CS solution, stirring for 2 h,	WCA (washing for 45 min, 10 cycles) = 125°,	
		3.) addition of PaNI to the immersed cotton, stirring for 6 more hours at 5 °C,	WCA (acid, alkali, 24 h) = 135°, WCA (peeling test, 50 cycles) = 151°,	
		4.) rinsing,	WCA (sandpaper test, 13 cycles) = 153°	
		5.) drying,		
		6.) immersion in ZnO solution		
		7.) air drying,		
		8.) immersion in STA for 3hours		
		9.) washing with ethanol, drying in a hot air oven.		
low pressure plasma (microwave plasma), dip-coating	silver nitrate (AgNO_3_)	1.) Preparation of cotton (scouring, desizing, bleaching),	WCA = 161°	^60^
plasma grafting (for activation)	oleic acid	2.) plasma pretreatment (activation and cleaning) with aragon (microwave plasmareactor; generator frequency: 2.45 GHz, discharge power: 500 W, exposure time 240 s, Ar flow rate; 60 mL/min, pressure 25 Pa),		
		3.) immersion in oleic acid solution,		
		4.) air drying,		
		5.) post plasma treatment (Ar plasma power:500W, exposure time 240 s, Ar flow rate; 60 mL/min, pressure 25 Pa),		
		6.) rinsing multiple time with ethanol.		

The technique offers several advantages, primarily its simplicity, minimal equipment requirements, and cost-effectiveness.^100,101^ The method is commercially scalable^100,104^ and compatible with a wide range of substrates and coating solutions.^104^ However, it is time-consuming due to the necessary slow withdrawal speed, possible multiple steps, and long drying and curing stages as the fabric is fully saturated in liquid. That is also why the method is more energy-consuming despite lower processing temperatures, which prolongs drying times and subsequently makes this method more time-consuming.^104^ Another disadvantage is the necessity for a high volume of coating solution and large tanks. Only about 20% of the solution in large tanks may be utilized effectively, even with long deposition life spans.^19^ Excess solution dripping can generate a significant amount of waste if not properly recovered and reused.^103,104,106^ Achieving precise and uniform film thickness is difficult, especially on porous and uneven textile surfaces.^100,104^ Additionally, the coating can impede the breathability of the textile, as highlighted in research by Sharif et al.,^107^ where it was noted that the air permeability experienced a reduction of approximately 20%., Films prepared using the dip-coating method can exhibit poor adhesion, which in turn leads to reduced durability and gradual deterioration, particularly after repeated washing or exposure to abrasion.^100,108,109^ Thick layers also influence material stiffness.^100^ This is especially relevant for films with poor interfacial adhesion to fibers, where the coating can be removed by washing or abrasion unless strengthened via a binder, cross-linking, or surface activation. Simple small-molecule hydrophobes often achieve high initial WCA, but leach rapidly.^63^ In the study by Huang et al.^63^ after 2 h of laundering at 40 °C, the betulin finish was largely removed, and the WCA dropped to ∼0° within 20 s, highlighting poor adhesion and durability on cotton. Incorporation into a copolymer (betulin terephthaloyl chloride - TPC) improved performance, though durability remained inferior compared to more strongly bound systems. Similarly, a CS–polyaniline–ZnO–stearic acid (STA) dip coating achieved superhydrophobicity (WCA ≈ 154°) and self-cleaning properties, but washing reduced performance (WCA fell to ∼125° in water and ∼118° with detergent after 10 cycles). Mechanical abrasion (∼13 scratch cycles) partially damaged the coating; while WCA remained >150°, the surface became sticky, indicating a high SA.

Xu et al.^4^ and Cheng et al.^110^ both utilized ESO in combination with sebacic acid and 1,8-diazabicycloundec-7-ene, but differed in layering: Xu et al. applied a single-layer system with STA post-treatment, achieving WCA of ∼159.7° while Cheng et al. added a secondary CNC coating, both involving curing steps at 150 °C achieving WCA of ∼155°. Zhang et al.^91^ achieved hydrophobicity of ∼133° using a zein-resin dip followed by water rinsing and drying. Pakdel et al.^69^ incorporated polydimethylsiloxane (PDMS) and melanin particles into an ultrasonic dip-coating bath, with air drying at room temperature, resulting in durable and high WCA of ∼164°. Banerjee et al.^87^ utilized Ag-CS NPS blended with natural rubber latex, applying the coating for over 24 h and curing it via UV exposure to ensure uniform coverage achieving WCA of ∼97–102°.

Singh et al.^26^ developed a coating formulation based on taro root extract, beeswax, and TiO_2_, incorporating preheating steps at 40 and 80 °C for wax melting and leaf drying, respectively, followed by air drying. Substrates coated with taro root extract alone exhibited a WCA of 96°, which increased to 112° with the addition of beeswax. The combination of taro root extract and TiO_2_ resulted in a WCA of 104°, while the ternary formulation containing taro root extract, beeswax, and TiO_2_ achieved the highest WCA of 128°, highlighting the synergistic effect of all components.

##### 4.1.1.2 Pad-dry-cure (PDC)

The PDC method (Figure 4, Table 3) consists of three main stages: padding (impregnation), drying, and curing. In the padding step, the fabric is passed through a solution containing the finishing agent, then squeezed by rollers to ensure uniform absorption and to remove excess liquid. This step ensures uniform absorption and controls the wet pickup, which can often be as high as 120% ± 5%^92^ or strictly regulated at 100%.^4,38^ This prevents chemical overuse and prepares the fabric for subsequent drying and curing. In the second stage, the fabric is dried—typically using hot air, infrared, or steam drying—to remove the liquid medium and promote adhesion of the finishing agent. Typically, drying occurs at moderate temperatures (e.g., 60 to 80 °C).^70^ This is followed by the final and crucial step of curing, where controlled heat, UV light, or chemical reactions fix the agent permanently to the fibers, ensuring durable performance and the desired fabric properties.^76^ The curing process uses higher temperatures, typically ranging from 150 to 160 °C. A pad-dry-cure line consists of a padder, predry/IR unit, drying tunnel, curing oven, and supporting equipment for tensioning, washing, and control.

**Figure 4:**
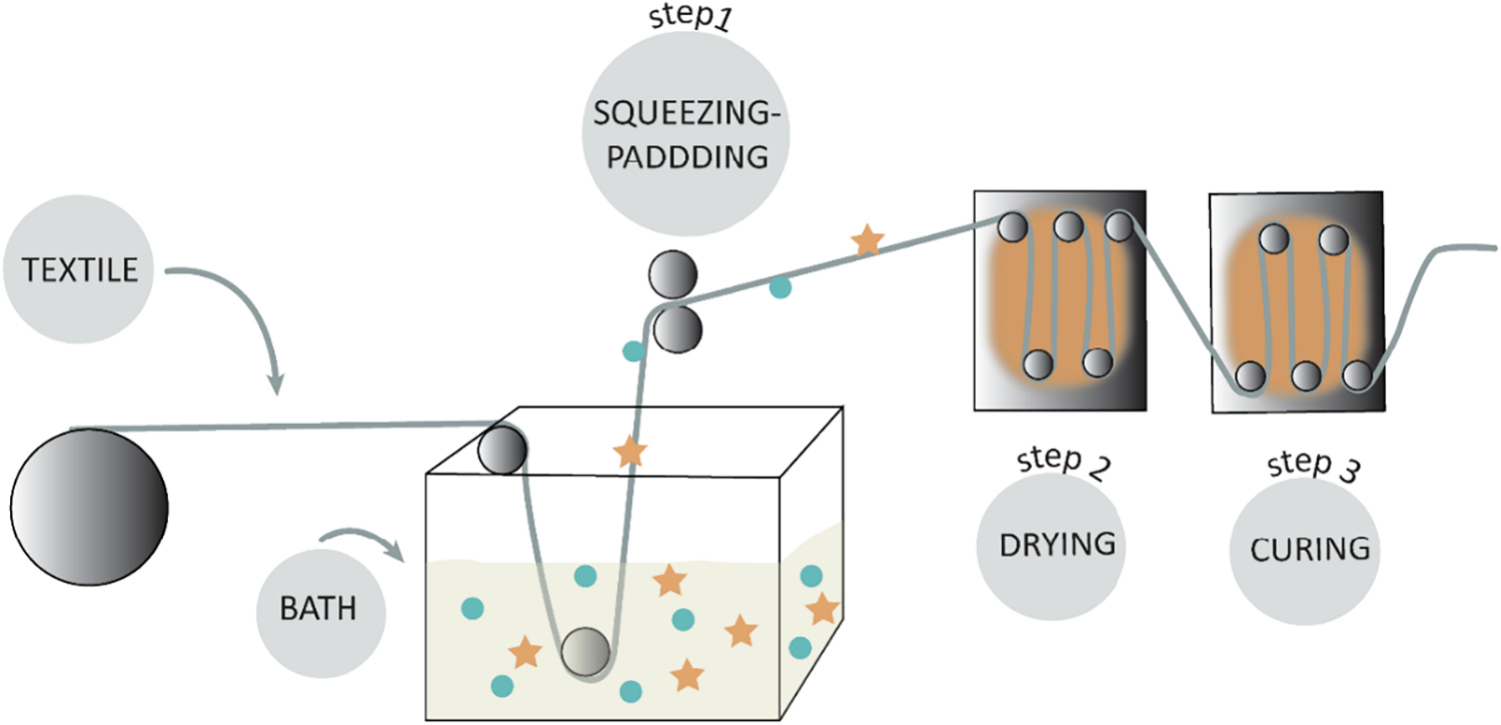
Schematic presentation of pad-dry-cure method: immersion in coating solution, padding/squeezing, drying and curing.

**Table 3:** Natural-Based Hydrophobic Coatings Using the PDC: Used Methods and Reaction Mechanism, Textile Substrate and Coating Materials, Process Steps, and Treatment Conditions, Hydrophobic Performance and Durability

Methods used and reaction mechanisms	Textile substrate and coating materials	Process steps and treatment conditions	Hydrophobic performance and durability	ref
NaOH pretreatment, PDC	100% cellulosic fibers	1.) Preparation of CNC, CS suspension (stirring at 60 °C for 2 h),	WCA = 135°	^80^
electrostatic interactions	CNC, CS	2.) pretreatment of cotton (scouring, cleaning)		
		3.) dipping for 1 h, padded via two dips and two nips,		
		4.) drying at 60 °C for 30 min, 5.) curing at 90 °C for 10 min.		
PDC	100% cellulosic fibers	1.) Pretreatment (soaking in water at 70 °C),	WCA = 97 (±6)°	^124^
grafting	CNC, butyl acrylate	2.) padding two dips and nips,		
		3.) drying at 80 °C, 3 min,		
		4.) curing at 160 °C, 3 min.		
PDC	100% cellulosic fibers	1.) Pretreatment of the cotton, fabric scouring at 40 °C,	WCA(0,05%melanin, PDMS) = 164 (±4)°	^69^
cross-linking	melanin NPS, PDMS	2.) immersion in a solution of melanin NPS, mixed with ultrasonic waves for 15 min,		
		3.) drying at room t overnight.		
sol–gel (silica), PDC	cellulosic fibers	1.) Preparation of silica sols,	sink in time = 35 s	^125^
electrostatic interaction, hydrogen bonding, dipole–dipole interactions	CS, silica sol	2.) standard coating padding,		
		3.) drying at 120 °C, 3 min.		
scouring, nanoseeding, growth of nanostructures, PDC	jute fibers and nonwovens	1.) Preparation of nanoparticle dispersion, ultrasonification for 30 min,	WCA (SA) = 120°, WCA (SA+ZnO) = 148°, WCA (SA+TiO_2_) = 132°	^126^
		2.) immersion for 10 min,		
		3.) padding via laboratory padder (two nips),		
		4.) dried in a convection oven at 80 °C, 5 min,		
		5.) curing at 150 °C, 10 min,		
physical interactions	STA, ZnO nanoparticles, TiO_2_	6.) immersion in fatty acid as a solution, padding (two nips),		
		7.) drying/curing at 80 °C for 5 min,		
		8.) room temperature again for 12 h.		
depletion, impregnation by dropwise addition, PDC	100% cellulosic fibers	1.) Preparation of zein solution,	WCA = 150°	^88^
physical interactions	zein NPS, ethanol	2.) addition of 70% ethanol,	WCA (10 laundry cycles) = stability,	
		3.) deposition/impregnation by dropwise addition, padding (best performing)	WCA (30 rubbing cycles) > 140	
		4.) Air-drying in an oven, 3 min, 80 °C,		
		5.) curing in an oven 2 min, 140 °C.		

The PDC coating method is relatively simple due to the primarily mechanical nature of the padding machines.^118^ It is an industrially recognized, cost-efficient and highly versatile method for imparting desired functional properties.^2^ The method offers high production efficiency because it is continuous and largely automated, enabling fast processing of large quantities of fabric for industrial-scale applications.^70,112^ PDC technique uses a high volume of water (or solvents) in the system tank, but retains a comparably low volume in the final product. This differs from simple dip coating or batch exhaust processes, which rely on draining rather than mechanical squeezing. This minimizes chemical wastage by ensuring excess finishing solution is removed, which can be recycled or reused in subsequent processes. Parameters like dye/coating concentration, pressure, temperature, and curing time can be precisely controlled, resulting in predictable and reproducible outcomes.^76,119–121^ Although the squeezing step reduces excess moisture, the substrates still require an extended drying period for complete water evaporation, potentially leading to increased energy consumption. PDC finishes generally provide uniform coatings with good adhesion and durability, as they generate stable cross-linked structures on the textile substrate. With a properly engineered stack (fiber activation → nanoparticle/binder pad → hydrophobe and cure), multiple studies demonstrate retention of superhydrophobicity after 25–30 laundering cycles and significant abrasion, while maintaining fabric hand and breathability.^80,118,122,123^ The high curing thermal treatment can sometimes negatively impact the inherent physical properties of cellulosic fabrics, leading to a decrease in softness, whiteness, and tensile strength due to possible fiber degradation.^92^

Several studies have applied the pad-dry-cure technique to treat natural-based hydrophobic coatings on textiles, demonstrating its adaptability with various bioderived agents. Yang et al.^80^ used a CS/CNC biocomposite, prepared via homogenization and thermal mixing, followed by a double-dip, double-nip padding process with subsequent drying at 60 °C for 2 h and curing at 90 °C for 10 min, resulting in WCA of ∼135°. Tian et al.^64^ used diluted persimmon tannin, with padding followed by curing at 120 °C for 20 min, achieving ∼145°. Zhang et al.^124^ treated cotton with CNC in combination with butyl acrylate (BA), resulting in WCA of ∼97°. Pakdel et al. presented strategy to endow cotton textiles with superhydrophobicity, UV shielding and photothermal heating by coating the fibers with natural melanin extracted from yak hair and a polydimethylsiloxane (PDMS) matrix via PDC; the melanin/PDMS layer achieves a water-contact angle of ∼164°, provides a UV-protection factor of ≈198, and raises surface temperatures to 45.3 °C under near-infrared illumination, while retaining high abrasion resistance, washing fastness and water-vapor permeability, thereby offering a sustainable multifunctional textile for personal thermal-management applications.

Despite variations in natural compounds and drying/curing parameters, these studies all highlight the effectiveness of the PDC method for uniformly applying biobased hydrophobic coatings to different natural fabrics.

##### 4.1.1.3 Spraying

It is a versatile technique employed to apply a coating or layer of material onto a surface by dispersing the coating material in the form of fine droplets through a spraying device.^101^ (Figure 5, Table 4) It is a contact-free approach suitable for any substrate material. It involves ejecting fine liquid particles by a jet stream of carrier gas onto the substrate.^101^ The dynamics of spray droplet impingement influence the results. Substrate properties such as roughness, permeability, and surface energy contribute significantly to droplet spreading and surface wetting.^101^ When the molten droplets collide with the cooler surface, they transform into delicate, plate-like particles that firmly adhere to the surface. These particles organize into layers, interlocking and overlapping before ultimately solidifying. The final coating thickness can be tailored based on the number of application passes, allowing for flexibility in achieving the desired thickness. Different experimental parameters influence the final coating. Those are the nozzle tip speed, solution precursor flow rate, number of spray cycles, substrate temperature, substrate type, substrate vibration, and distance between the nozzle and substrate.^101^ There are two main types of spraying techniques: cold and thermal spraying, which are, at their core, dry techniques. However, to our knowledge, they have not been used for hydrophobization of natural textiles with natural compounds so far.^127^ Thermal spraying techniques use high heat sources to melt or partially melt feedstock materials before propelling them onto a substrate, typically for robust, thick metal or ceramic coatings.^127^ Methods include atmospheric plasma spraying (7500–13500 °C), high-velocity oxy-fuel spraying (≈2500 °C, supersonic combustion jet), and flame spraying (≈2500 °C, fuel gas combustion).^127^ In contrast, the cold spray technique accelerates solid particles to supersonic speeds using pressurized gases (e.g., N_2_, He), bonding them through kinetic rather than thermal energy—ideal for heat-sensitive materials like polymers and textiles. High-pressure cold spray achieves particle speeds up to 1200 m/s, while low-pressure systems are more straightforward and cheaper but less efficient.^127^ In the context of textile functionalization, where heat sensitivity and material properties are critical, a third, more application-specific category often emerges: wet/solution-based spraying, which relies on liquid precursors and moderate curing temperatures rather than high-energy plasma or combustion flames.^58^ In this category are air-spray coatings, which use a spray gun or nozzle pressurized by air.^102,128^ Electro spray applies the coating material to a substrate using a high-potential difference to influence droplet deposition, often yielding uniform parts in the nanoscale.^102^ Solution/suspension precursor spraying utilizes nanoscaled powders or solutions (e.g., sol–gel precursors) as the injection medium in spray applications, tailored for specific microstructures.^127^ Atmospheric pressure plasma jet spraying, while technically utilizing plasma, it is often classified near solution methods for textiles because it operates near room temperature and is suitable for in-line application of liquid/gaseous precursors onto heat-sensitive materials.^127^

**Figure 5:**
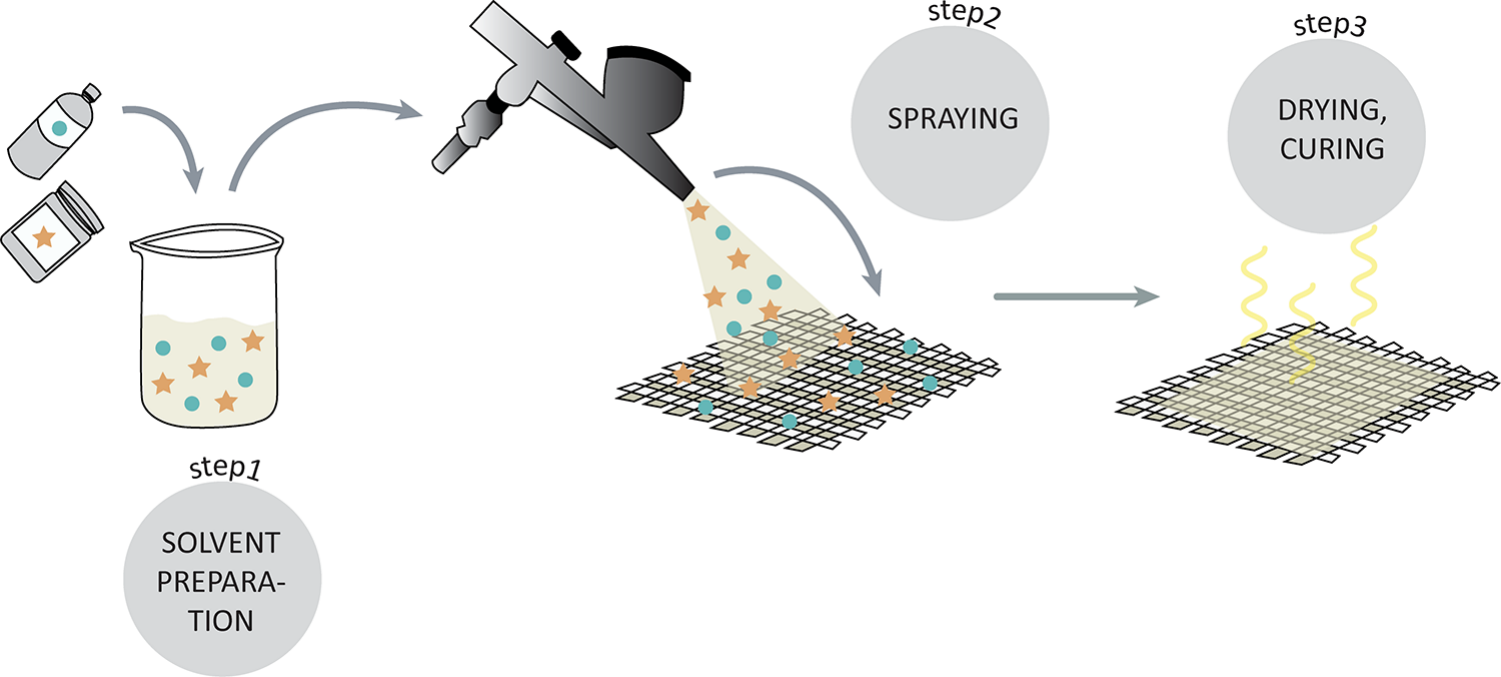
Schematic presentation of wet/solution-based spraying: solvent preparation, spraying, and drying/curing step.

**Table 4:** Natural-Based Hydrophobic Coatings Using the Spraying Method: Used Methods and Reaction Mechanism, Textile Substrate and Coating Materials, Process Steps, and Treatment Conditions, Hydrophobic Performance and Durability

Methods used and reaction mechanisms	Textile substrate and coating materials	Process steps and treatment conditions	Hydrophobic performance and durability	ref
spray coating	100% cellulosic fibers	1.) Cleaning of cotton in ethanol using ultrasound for 10 min, drying at 80 °C for 1 h,	WCA = 155°	^98^
		2.) preparation of ZnO as a suspension, using ultrasound for 30 min,	WCA (chemical stability in 1–13pH) > 150°, WCA (after immersion in water and oil for 10 days) > 150°	
esterification	ESO + sebacic acid = CESO, ZnO, STA	3.) spraying with adhesion solution,		
		4.) drying in a fan-assisted oven for 30 min,		
		5.) spraying of ZnO solution, drying at 150 °C for 3 h,		
		6.) immersion in STA solution,		
		7.) drying at 120 °C for 1 h.		
spray coating	100% cellulosic fibers	1.) Preparation of beeswax, lignin solution, heating at 100 °C for 20 min,	WCA = 150°	^84^
physical interactions (hydrogen bonding)	beeswax, lignin, N-hexane	2.) spraying	WCA (chemical stability, pH = 2, pH = 13) > 150°,	
		3.) drying in room T for 24 h.	WCA (thermal stability, 120 °for 60 min) = 135°	
spray coating	cellulosic fibers	1.) Preparation of PTA, CNC, stirring at room T, 24 h,	WCA = 140°	^94^
self-polymerization, grafting	CNC, ODA, self-polymerized TA	2.) ODA grafting reaction, stirring at 50 °C for 25 h,	WCA (detergent washing for 30 min) > 140°	
		3.) freeze-drying of the solution for 1 week,		
		4.) spraying,		
		5.) drying at ambient temperature.		
spraying, UV-assisted Click reaction	cellulosic fibers	1.) Precursor and solution preparation,	WCA = 168°	^131^
grafting	rosin acid, SiO_2_	2.) spray impregnation,	WCA (110 abrasion cycles with 1000mesh standpaper) = 154 (±0.2)°, WCA (immersion in 1 wt % detergent solution, at 300 rpm at 50 °C, 50 cycles) = 153°,WCA (immersion in pH = 1, pH = 13, organic solvents for 24 h) 154 (±2.3)°	
		3.) UV-assisted irradiation for 30 min,		
		4.) drying/curing at 60 °C for 2 h.		
plasma pretreatment with oxygen, spraying	surgical-grade cotton	1.) Substrate pretreatment (oxygen plasma),	WCA = 159 (±3)°	^81^
cross-linking, grafting	CS, ODA, glutaraldehyde	2.) synthesis of coating material,	WCA (55 cycles of rubbing on 600 grit sandpaper under a 150 g load) = 156°,	
		3.) spray coating via pen gun,	WCA (80 cycles, adhesive tape peeling) = only slight decline in WCA	
		4.) drying in ambient conditions.		
plasma pretreatment with oxygen, spraying	100% cellulosic fibers	1). Synthesis of coating material (ODA dissolved in ethanol at 50 °C, addition of GA, stirring for 4 h, isolation),	WCA = 152 (±3)°	^40^
oxidation, cross-linking	CNF (from hardwood pulp), ODA, glutaraldehyde (GA)	2). plasma pretreatment o of cotton (microwave plasma, 200 W, 2 min),	WCA (exposure to natural sunlight for 32 days, 5 h per day) = 148°, WCA (30 washing cycles in lukewarm soapy water, 50 °C, stirred for 30 min) = 134°, WCA (rubbing on a ceramic tile for 500 times) = 150°	
		3). spray coating using pen gun and nitrogen as the blowing gas,		
		4). drying at 60 °C for 30 min.		

Although the spraying process is conceptually simple, its successful execution requires a high level of expertise and precise control. The technique is quick and cost-efficient. Spray lines require only pumps, nozzles, and ventilation, resulting in a lower capital cost compared to vacuum plasma or supercritical CO_2_ systems.^129^ Managing waste from spray coating typically involves ensuring the excess material is collected and either recycled or disposed of properly. Overspray collection and filtration systems (e.g., booth curtains, centrifugal separators) are needed, but their footprint is modest and has little impact on land use.^24^ The amount of water and solvent used can vary depending on the type of coating material and the specific application process.^101^ Compared to padding, modern spray/atomization, or nebulization platforms work at liquor ratios as low as 1:0.2 and reduce process water/solvent by 80–90%.^24^ Because only the droplets that reach the fabric are needed for film formation, reagent overdosing is lower than in batch baths, reducing chemical loss and subsequent wastewater load.^79^ Spray and other low-wet-pickup techniques can lower thermal-drying demand by roughly 30–40% because less liquid must be evaporated compared with conventional pad-dry-cure routes.^123^ Industrial monorails or roll-to-roll spray lines have demonstrated speeds compatible with existing finishing ranges, while enabling single-side or double-side deposition in a single pass, thereby improving overall line productivity.^24^ In wet spraying, the most significant factor determining energy consumption is the evaporation of the liquid medium (water or solvent) and the thermal curing of polymer coating.^24^ Curing processes often require high temperatures from 80 to 220 °C to accelerate chemical bond formation and ensure coating durability.^24,70^ Spray can dispense molten lipids, nanoparticle slurries, sol–gel precursors, water-borne polymers, or reactive monomer blends, enabling fluorine-free, biobased, or inorganic routes to hydrophobicity.^79^ Because the process is contact-free and low-pressure, it can handle delicate silk, heat-sensitive synthetics, or thick 3-D spacer fabrics where dip coating and PDC are ineffective.^129^ Unused droplets can be recirculated. High-transfer-efficiency or electrostatic guns minimize fugitive emissions. Collection systems demand limited plant floor space, so land-use increment is negligible relative to legacy finishing halls. Cold-sprayed powders remain largely unmelted, enabling easier mechanical or solvent recovery of the coating material during recycling streams. In contrast, aqueous spray finishes using nanocellulose or polysaccharides are compatible with paper/fiber repulping processes.^79^ Cold spraying techniques also offer other significant sustainability benefits, including energy efficiency, material preservation, and adaptability to various feedstock materials. While challenges such as high gas consumption and limited polymer applications persist, ongoing advancements in cold spray technology and the adoption of renewable materials could further improve its environmental performance.^130^ The spray coating process, especially when using nanodispersions, can be controlled to coat the fibers without completely blocking the macroscopic pores of the textile structure, thus helping to maintain acceptable air and water vapor permeability. All the observed spray-coated studies achieved excellent hydrophobic performance at around ∼150° or higher. Chen et al.^131^ sprayed a solution of rosin acid modified with SiO_2_, followed by UV-click curing, and achieved a WCA of ∼168° with high abrasion, washing, and chemical resistance, maintaining a WCA higher than ∼150 °C even after 110 abrasion cycles, 50 washing cycles, and 24 h of immersion in an organic solvent. Roy et al.^40^ applied a dispersion of CNF and ODA, prepared via ultrasonic treatment and magnetic stirring, using a nitrogen-assisted pen gun for precise deposition. An oxygen plasma pretreatment enhanced adhesion between the coating and fabric, resulting in a superhydrophobic surface with a WCA of 162° and excellent mechanical durability, maintaining high hydrophobicity even after abrasion and multiple washing cycles. Xiang et al.^94^ reported a facile, environmentally benign route to fabricate superhydrophobic CNCs that can be readily integrated into a wide range of substrates, from paper and textiles to metal and polymer surfaces, without the need for fluorinated reagents. The authors first isolated CNCs from wood-derived cellulose by sulfuric-acid hydrolysis, then modified the nanocrystals with a low-surface-energy silane (hexadecyltrimethoxysilane) through a simple condensation reaction performed in aqueous media at ambient temperature; the silane grafting simultaneously introduced nanoscale roughness and hydrophobic alkyl chains, yielding a water-contact angle exceeding ∼150° and a sliding angle below 10° (Cassie–Baxter regime). Because the modification is covalently bound to the CNC surface, the resulting particles display excellent chemical stability, UV resistance, and mechanical durability, maintaining superhydrophobicity after repeated abrasion cycles, washing tests, and exposure to acidic or alkaline environments. The versatility of the engineered CNCs was demonstrated by dip-coating or, when higher throughput was required, by spray-coating, both of which produced uniform, transparent superhydrophobic films on various substrates, including cotton.

Zhang et al.^132^ sprayed a beeswax–lignin suspension, prepared by melting and mixing the components in *n*-hexane, onto cellulosic fabric from a fixed 12 cm and at a constant pressure. Hydrophobicity was achieved after resting the coated fabric at room temperature for 24 h, yielding a water contact angle of ∼155° and good washing durability due to the strong interfacial interaction between lignin and cellulose. The studies highlight the flexibility of spray methods in achieving controlled and uniform deposition of biobased coatings.

##### 4.1.1.4 Casting Techniques

Casting techniques are presented in Table 5. From these techniques, solvent casting—schematically illustrated in Figure 6a—is the most employed method. It is utilized initially for film formation at both laboratory and pilot scales. This technique involves dissolving or dispersing a selected compound in a suitable solvent.^133^ The chosen solvent must wet the fibers yet allow rapid drainage to avoid excessive add-on. Viscosity (1–200 mPa·s) is tuned with solids content (2–30 wt %) and rheology modifiers. Uniform wetting promotes monolayer-like coverage, ensuring low contact-angle hysteresis. Too-high viscosity traps air bubbles, while too-low viscosity leads to runoff and weight gain. Then the fabric is moved under a doctor blade or slot die, carrying a metered wet film (typical wet pickup: 10–60 g/m^2^). The resulting solution is then poured into a mold and allowed to dry, enabling solvent evaporation and the formation of a film that adheres to the mold. Alternatively, the compound can be cast onto a release paper, partially dried, and then laminated (transfer casting) onto the fabric under mild pressure. Knife gap, web tension, and forward speed set wet-film thickness.^134^ Various air-drying mechanisms, such as hot air ovens, tray dryers, microwaves, and vacuum dryers, aid in the casting process by facilitating solvent removal and film peeling. Temperatures are kept at 60–140 °C for aqueous or low-boiling organic systems. The air-drying stage is crucial, as it enhances the intramolecular relationships between polymer chains and contributes to achieving an optimal microstructure for the film.^102^ Fabric porosity allows two-sided evaporation, accelerating solvent removal but risking ≫coffee-ring≪ edge build-up; adjustable airflow and web oscillation mitigate this. Excess shrinkage is minimized by tentering. Complete solvent removal (<1 wt %) is critical as residual solvent disrupts hydrophobic molecular orientation, lowering WCA and increasing tackiness or odor.^133^ When compared with dip-coating or spray application, casting allows for a longer residence time for polymer chain rearrangement. The final stage is postcure/cross-linking. This technology offers notable advantages, including consistently even thickness distribution, optimal optical clarity, and remarkably low haze.^135^ The technique is relatively simple.^130^ However, it is generally time-consuming due to slow solvent evaporation and diffusion, which is also why the technique is costly and impractical for large-scale production. Despite this, solvent casting is viable at an industrial scale in roll-to-roll mode.^136^ Additionally, limited solvent diffusion within the film and the requirement for solvent recovery further reduce the overall processing speed.^136^ The energy consumption associated with the solvent casting technique is generally low for the initial processing step. Still, it can become significantly high and costly due to the required solvent management and extended drying times. Solvent recovery requires high energy input and is cited as a primary reason why solvent-cast products are often more expensive to manufacture than extruded films.^136^ For eco-friendlier water-based coatings, the low volatility of water necessitates a slow and extended drying process, leading to a higher amount of energy consumption for continuous drying and curing operations.^130^ Modern continuous solvent-casting lines (belt or drum) routinely run at 10–60 m/min, which translates (depending on film width) to >600–6000 m^2^/h of product area output. The rapid expansion of liquid crystal display applications has spurred advancements in materials and enhanced techniques for solvent casting and coating processes.^136^ Nevertheless, solvent-casting technology is not universally applicable to all types of polymer films. It is best suited for a specific range of materials that can be transformed into films. Films with a thin thickness cannot be extruded without stretching and producing excessively thick films. Consequently, products created through solvent casting tend to incur higher costs.^136^ Water-based coatings are more cost-effective.^130^ The process often relies on organic solvents, which are nonrenewable, emit harmful volatile organic compounds, and pose environmental and health risks during evaporation. Replacing them with water or low-toxicity alternatives aligns with green chemistry principles. Solvent casting can further generate toxic solvent waste, as these solvents dissolve polymers for coating applications and can be hazardous if not properly managed. The focus is on minimizing residual solvent, optimizing drying time, and achieving defect-free polymeric coatings while utilizing cost-effective, eco-friendly, and easily recoverable solvents.^130^ Scaling up of this technique may require careful control of solvent evaporation to prevent uneven coatings or fiber stiffening.^137^ Solvent casting allows controlled deposition of hydrophobic agents, such as long-chain fatty acids, waxes, or modified polysaccharides, which penetrate the surface pores of the textile fibers and create a continuous coating. The method also enables tuning of coating thickness and surface roughness, both critical factors for enhancing water repellency. Moreover, solvent-cast coatings generally exhibit good adhesion and moderate durability against washing and abrasion. However, performance depends on the choice of polymer, solvent, and post-treatment (e.g., curing or heat-setting).^130^ Huang et al.^63^ surface-coated films from betulin and betulin copolymer as coating on cellulosic fibers fabric samples by compress molding instrument under a pressure of 80 kN at 210 °C for 4 min. The same coatings were also applied using a dip-coating (immersion) technique, as previously discussed. Fabrics treated by the solvent-casting method exhibited static WCAs of ∼123° for betulin film-coated fabric and ∼123° for 104° for betulin-TPC copolymer film-coated fabric. In comparison, dip-coated fabrics achieved much higher initial WCA; however, solvent-cast or film-coated fabrics maintained significantly better durability, achieving a WCA of ∼80°. These results demonstrate a trade-off between initial hydrophobicity and coating stability, with solvent casting offering superior adhesion and long-term performance.

**Figure 6:**
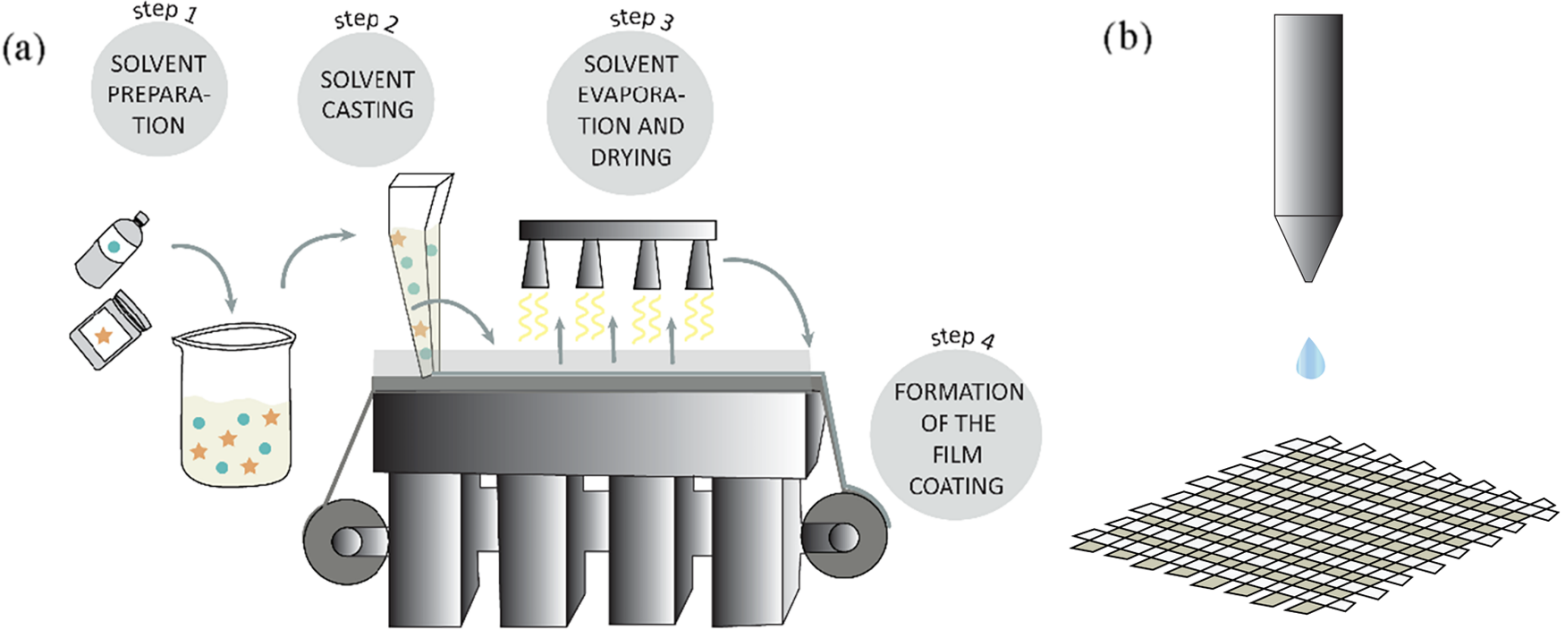
Schematic presentation of casting techniques: (a) solvent casting: solvent preparation, solvent casting, solvent evaporation, drying, film formation, (b) drop casting technique.

**Table 5:** Natural-Based Hydrophobic Coatings Using Solvent Techniques: Used Methods and Reaction Mechanism, Textile Substrate and Coating Materials, Process Steps, and Treatment Conditions, Hydrophobic Performance and Durability

Methods used and reaction mechanisms	Textile substrate and coating materials	Process steps and treatment conditions	Hydrophobic performance and durability	ref
pretreatment, solvent casting, and impregnation by compression molding	100% cellulosic fibers	1.) Cotton retreatment (washing),	WCA (a) = 123°, WCA (b) = 104°	^63^
		2.) soaking fabrics in berulin/betullin-TPC solution,		
		3.) air-drying,	WCA (washing in washing machine, 40 °C for 2 h), solution a = 80°,	
esterification	a) botulin, ethanol, b) betulin-TPC copolymer in tetrahydrofuran	4.) oven drying,		
		5.) dissolving cta with coating solution, solvent casting		
		6.) hot-pressing of the films on cotton.		
drop casting, dip-coating physical interactions (hydrogen bonding)	100% cellulosic fibers	1.) Preparation of components,	WCA (eggshell powder, beeswax) > 90°	^65^
		2.) deposition of eggshell powder in ethanol via drop casting,		
		3.) air drying,		
	eggshell powder, food-grade natural beeswax, bovine serum albumin, hexane, ethanol	4.) immersion in Beeswax solution,		
		5.) drying at 60 ° for several hours.		

For coatings on small surfaces, using a minimal amount of solvent, the drop casting (Figure 6b) method can be employed. In this technique, a solution is poured onto a substrate in the form of drops and allowed to dry without spreading. When multiple droplets are cast, they mix together upon contact, forming a noncircular decline with a concave contact line. Gonçalves et al. used a dropwise impregnation (form of drop-casting), along with depletion and padding, to impregnate cellulosic fibers with free zein solutions (10–50 g/L in 70–90% ethanol).^88^ While dropwise impregnation did successfully impart initial hydrophobic character WCA > 90° to the cellulose fibers, it did not achieve the persistent performance necessary for a commercial textile compared to other methods. The padding method, which fully impregnates the fabric and achieves maximum material uptake, ultimately produced a superior, more cohesive, and thicker hydrophobic zein film, providing the most robust and durable antiwetting properties.

##### 4.1.1.5 Spin Coating

Although traditionally favored for rigid substrates, the spin coating technique (schematically presented in Figure 7, Table 6) serves as a powerful method for fabricating uniform, nanoscale hydrophobic coatings on flexible, porous natural textiles by precisely controlling the surface morphology and chemical composition, the two prerequisites for achieving superhydrophobicity.^67,108^ The technique involves four main stages: solution dispensing, rotation-dominated thinning (where the spin-up material starts spreading on the substrate/spin-off material is entirely on the substrate), solvent evaporation, and curing.^101^ The prepared textile substrate is securely mounted on a rotating platform, often on a rigid support (such as a glass slide). The coating solution is dispensed onto the center of the textile while it is stationary (static dispense) or at a low rotational speed.^67,101^ The process typically involves two phases: spin up (or spread phase) where the speed is gradually increased (e.g., up to 1000 rpm for lab scale) to spread the solution across the substrate, and spin off (or spin–out phase) where high speeds (e.g., 2000 to 6000 rpm) fling excess liquid off the surface due to centrifugal force. High spin speeds are generally recommended for achieving maximum uniformity.^101,138^ As the solvent evaporates, the solution thins, and the dissolved material deposits onto surface asperities. The textile is then subjected to a postprocessing step involving heat (e.g., curing at 120 to 160 °C) to remove residual solvent, solidify the coating, or promote cross-linking for durability.^67,124^ Heating treatments, such as steam ironing or oven drying at 70 to 120 °C for short periods, can be used after washing to restore any lost hydrophobicity (self-healing effect) by inducing the migration and reorientation of long alkyl chains from the interstices back to the surface. Spin coating is generally considered a simple, relatively quick, versatile, and cost-effective method for production.^101^ High-speed rotation drives most of the motor/electronics load, especially during multistep spin programs and long spin times, which is responsible for the energy consumption. Thicker or more viscous formulations, along with lower solvent volatility, increase spin duration and thus motor time. Although lots of the solvent evaporates during spinning via airflow, many hydrophobic coatings still require thermal or UV post-treatment to cross-link or consolidate, and ovens/UV lamps can dominate total energy use if used (similar to other coating routes.^101^ Spin coating is, in general, one of the most widely used techniques at the laboratory scale due to its excellent reproducibility and compatibility with a broad range of solution viscosities.^99^ However, it is not well-suited for industrial scale-up because it typically results in significant material waste (most dispensed precursor solution is flung off the substrate surface during the spin-off phase due to centrifugal force) and is limited in its ability to coat large-area substrates.^101^ Spin-coating fundamentals explicitly allow water or organic solvents; the solvent choice primarily affects evaporation rate and film quality. In practice, aqueous systems demand tighter control (wetting/surfactant, humidity, temperature, and spin recipe) because water evaporates more slowly (lower vapor pressure, higher latent heat) and is more sensitive to “coffee-ring” and drying artifacts.^101^ A spin-coating system consists of a high-speed motor that drives a precisely controlled rotary stage, a vacuum-or mechanically clamped chuck to secure the substrate, a dispense unit that delivers a measured droplet of coating solution onto the substrate center, and a containment bowl or sealed chamber to capture the excess fluid expelled by centrifugal forces; the motor controller regulates acceleration, spin speed and dwell time, while integrated temperature and humidity sensors—or an optional heated substrate holder—enable real-time management of solvent evaporation. A peripheral exhaust or solvent recovery line can be installed to capture volatile organic compounds (VOCs) during the spin-off and drying phase.^101^ The spin-coating tool has a minimal direct land footprint; sustainability hinges on choosing low-impact (ideally biodegradable) chemistries from waste/secondary biomass, applying them in water, and routing coated products into compatible end-of-life systems (recycling or organics) as defined by established standards.^79,139^ Spin coating can yield functional textiles with acceptable mechanical and environmental durability for specific, nonmass production applications.^140^

**Figure 7:**
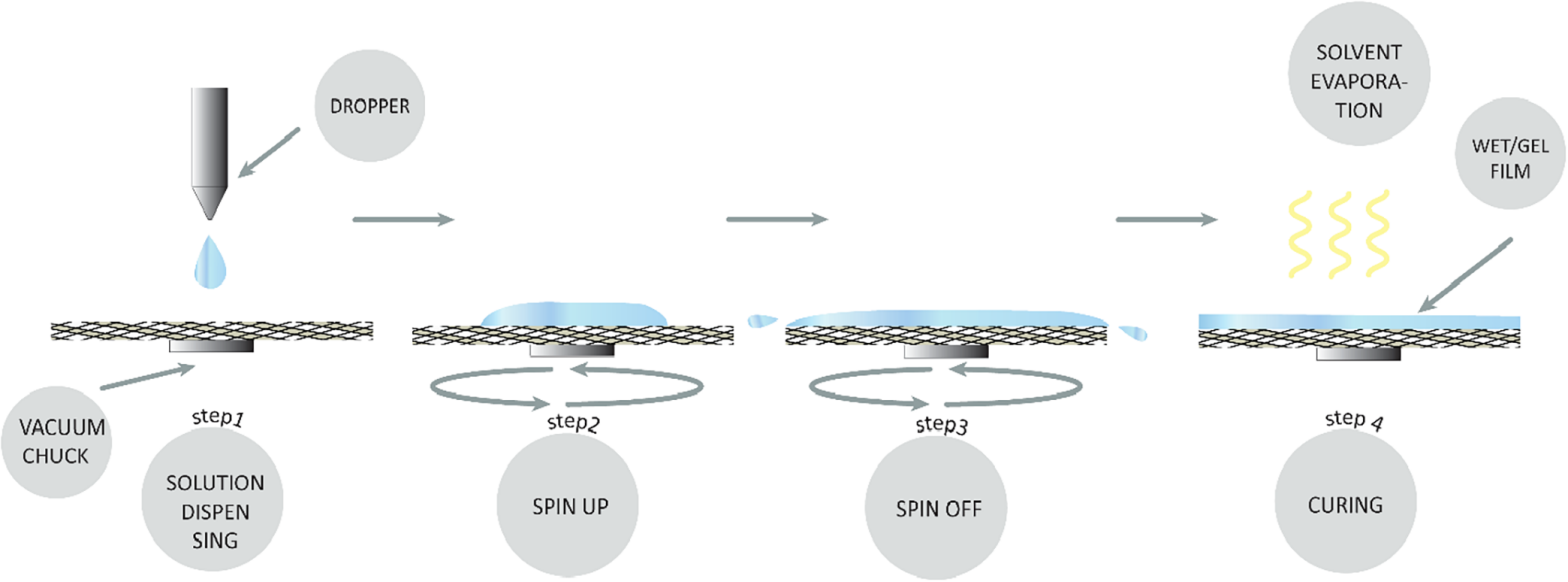
Schematic presentation of spin-coating: solution dispensing, spin up, spin off and curing step.

**Table 6:** Natural-Based Hydrophobic Coatings Using Spin-Coating Techniques: Used Methods and Reaction Mechanism, Textile Substrate and Coating Materials, Process Steps, and Treatment Conditions, Hydrophobic Performance and Durability

Methods used and reaction mechanisms	Textile substrate and coating materials	Process steps and treatment conditions	Hydrophobic performance and durability	ref
Sol–gel preparation of NPS, dip-spin coating	100% cotton	1.) Synthesis of TiO_2_–SiO_2_ via sol gel, stirring, 30 min at room T,	WCA = 90 °C	^141^
		2.) adding CS powder, stirring, aging for 15 h, drying at 110 °C		
		3.) pretreatment of cotton		
Grafting	TiO_2_–SiO_2_, CS, hexadecyltrimethoxysilane (HDTMS	4.) spin coatings, 15 min at 80 °C,		
		5) immersion in HDTMS solution, placed in the autoclave for 3 h at 120 °C.		

The study by Rilda et al.^141^ synthesized a TiO_2_–SiO_2_/CS composite via a sol–gel route using titanium isopropoxide/diethanolamine (TIP/DEA) and Tetraethyl Orthosilicate/Hydrochloric Acid (TEOS/HCl) precursors in isopropanol, mixed with CS (in acetic acid) and Cetyltrimethyl Ammonium Bromide (CTAB). The sol was aged (15 h), dried (110 °C, 3 h), and calcined (500 °C) to yield the composite powder. Precleaned cotton fabrics were impregnated with the solution at 100 °C, cross-linked with butanetetracarboxylic acid (BTCA) (24 h), and coated with the TiO_2_–SiO_2_/CS dispersion via dip–spin coating. After drying and washing, the fabrics were treated with hexadecyltrimethoxysilane (HDTMS) and cured at 120 °C for 3 h to form a hydrophobic silane layer. The treatment converted the fabric from fully hydrophilic to hydrophobic, with a WCA of ∼90°, and maintained stability for over 100 min.

#### 4.1.2 Self-Assembly Techniques And Molecular Structuring Techniques

Self-assembly is the spontaneous organization of components into ordered structures through local interactions, without external direction.^58,103,142^ It is a process in which pre-existing parts arrange themselves into a larger, organized structure through specific interactions among the components. These interactions can be noncovalent, such as hydrogen bonding, van der Waals forces, electrostatic forces, or π-π interactions.^103^ These techniques enable the creation of low-surface-energy coatings or the deposition of NPS to enhance surface functionality.

##### 4.1.2.1 Layer-by-layer (LBL) Self-Assembly

It is a technique used to deposit thin films or coatings onto surfaces by leveraging electrostatic interactions between adjacent layers (Figure 8, Table 7). This method entails the stepwise deposition of positively and negatively charged materials in alternating layers onto a substrate, resulting in a multilayered coating with organized surface features. Initially, a negatively charged substrate is utilized, typically immersed in a polycation solution. The polycations adhere to the substrate surface for an optimized duration, dependent on the specific molecular properties. This crucial step facilitates the creation of a uniform and firmly adhered layer. Following this, the substrate is extracted from the solution, thoroughly rinsed with deionized water for several minutes, and then dried. Subsequently, the substrate undergoes immersion in a polyanion solution. Consequently, the overall solution maintains electrical neutrality.^143,144^ Afterward, a similar rinsing and drying procedure as described earlier follows. Various application methods, such as spraying, dip-coating, and solution casting, can be employed for this deposition process.^143^

**Figure 8:**
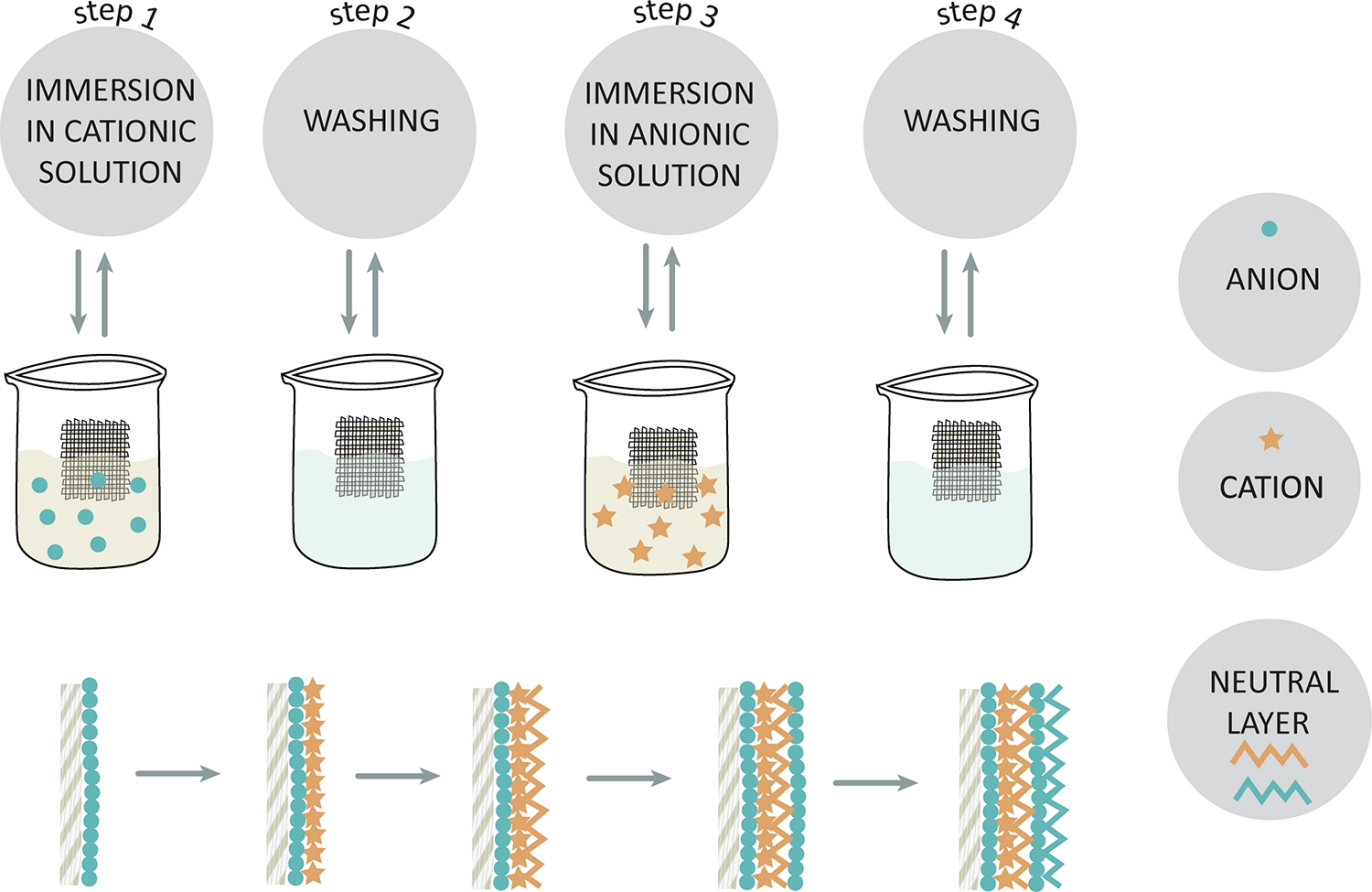
Schematic presentation of LBL self-assembly: alternating immersion of the substrate in cationic and anionic solutions, with rinsing steps in between. The cycle is repeated to build up the desired number of layers.

**Table 7:** Natural-Based Hydrophobic Coatings Using the LBL Self-Assembly: Used Methods and Reaction Mechanism, Textile Substrate and Coating Materials, Process Steps, and Treatment Conditions, Hydrophobic Performance and Durability

Methods used and reaction mechanisms	Textile substrate and coating materials	Process steps and treatment conditions	Hydrophobic performance and durability	ref
LBL, dipping/spraying/brushing	100% weave cotton, 100% knit cotton, blend (55% hemp, 45% natural carnauba wax)	1.) Preparation of the textiles (soaking in water),	WCA (cotton, carnauba wax+PLL) = 155 (±15)°, SA (cotton, carnauba wax+PLL) = 9°	^59^
electrostatic attraction	natural carnauba wax, PLL/cationic starch	2.) immersion for 5 min in polycation solution (subsequently in anionic wax solution),		
		3.) rinsing three times,		
		4.) drying at room temperature,		
		5.) repeating steps 2–4,		
		6.) curing at 70° at least 15 min.		
LBL self-assembly vid pad-dry-cure	100% cellulosic fibers	1.) Synthesis of solid carnauba NPS (sonification for 4 min, 100W power, rapid cooling),	WCA = 131°WCA (washing stability) = 102°	^35^
electrostatic attraction	carnauba wax NPS, chitosan	2.) pretreatment of fabric (scouring, aminization, pH adjustment)		
		3.) immersion in anionic solution (solid carnauba NPS) and subsequently in cationic (chitosan) solution for 5 min, padding,		
		4.) drying and curing in an oven at 70 °C for 15 min after each immersion,		
		5.) repetition of steps 3–4.		
plasma pretreatment with O_2_ (atmospheric plasma), LBL	100% cellulosic fibers	1.) Fabric preparation and activation (scouring, atmospheric plasma pretreatment with O_2_, -frequency 25 kHz, power 2,5 kW, line speed 1m/min, 6 repeated passes),	WCA = 131°	^36^
Oxidation (pretreatment), electrostatic attraction	carnauba wax NPS, ZnO NPS	2.) preparation of carnauba NPS emulsion (sonication for 4 min, 30 kHz, 100 W),	WCA (washing durability) = 103°	
		3.) preparation of ZnO nanoparticle dispersion (ultrasonification at 20 W, 10 min),		
		4.) immersion in ZnO suspension (subsequently in carnauba wax NPS) for 5 min, pad to 100% wet pick up,		
		5.) oven drying at 70° for 15 min after each immersion,		
		8.) repetition of steps 4 and 5		
		9.) final curing at 70° for 15 min.		
LBL self-assembly	100% cotton	1.) Preparation of cotton,	WCA = 90 (±8)°	^147^
electrostatic interactions	CS, poly(sodium-4-styrenesulfonate-PSS)	2.) immersion of PSS anionic solution, 5 min at room T, and subsequently cationic (chitosan) solution,		
		3.) standard washing (rinsing in distilled water for 5 min) or ultrasonication-assisted washing after each immersion,		
		4.) oven drying at 60–80° for 10–15 min after each immersion,	N/A	
		5.) drying in oven at 60 °C for 10 min after each bilayer,		
		6.) repetition of steps 3–5,		
		7.) final curing in the oven at 70–80 °C, 30 min.		

The technique is simple, cost-effective, and robust. It requires minimal technology and can be easily optimized, reducing equipment costs. LBL assembly requires lower amounts of colloids to produce functional coatings compared to conventional techniques. Only about 1% of colloids are needed in LBL, while traditional methods require a minimum of 10%.^144^ Water-soluble polyelectrolytes are primarily used as coating materials in LBL assembly, reducing the need for organic solvents.^144^ No high-energy inputs are needed for the synthesis of materials during deposition. However, the technique’s energy consumption also raises potential concerns, as it can be high due to multiple washing and drying steps, which can also be time-consuming. LbL hydrophobic coatings are technically scalable today via spray-LBL on roll-to-roll finishing lines for outdoor and lifestyle textiles. The biggest industrial barrier is the wash/abrasion durability of purely electrostatic stacks.^59,145^ The waste generated primarily comes from unabsorbed materials that are washed off during the rinsing steps. Given that LBL involves the deposition of charged materials through controlled electrostatic interactions, it does not generate significant chemical waste or byproducts during the deposition process. Through the application of precise stoichiometry, optimization is straightforward and not reliant on intricate chemical reactions for layer deposition. This versatile technique accommodates a broad spectrum of deposition materials. LBL is compatible with full biodegradability if degradable polyelectrolytes (e.g., CS/alginate/cellulose) are selected and natural hydrophobes.^146^ Furthermore, the method offers control over coating thickness, enabling the creation of ultrathin films as slim as 1 nm.^143^ This is why they cause only a marginal reduction in air permeability. At the same time, LBL depositions do not typically form a continuous film that blocks the pores of the fabric, unlike conventional coatings.^147^ In their purely electrostatic form, as already mentioned, they are typically less wash-durable than other finishes that are chemically cross-linked. With chemically locked or primer-assisted LbL, stable hydrophobicity after multiple wash cycles and abrasion is achievable. Introducing ultrasonication during the intermediate rinsing steps is an effective method for removing loosely held polyelectrolyte layers. This action results in a more uniform surface deposition, often eliminating surface cracks observed in nonultrasonicated samples, thereby enhancing the coating’s durability and preserving its functional attributes, such as antimicrobial activity, after washing.^147^ Bashari et al.^35^ demonstrated retained hydrophobic performance with carnauba wax NPS and CS LbL after washing protocol. The initial WCA dropped from 131° to 102°. The coating also preserved air permeability and antibacterial performance. After each immersion, the samples were carefully padded and then dried in an oven set at 70 °C for 15 min. In a separate study, Bashari et al.^36^ combined carnauba wax with ZnO on plasma pretreated cotton, both achieving stable coatings after five bilayers achieving WCA of ∼131°. Joshi et al.^147^ used CS and oppositely charged polyelectrolytes to form up to 20 bilayers on cotton, enhanced by ultrasonication during washing, with contact angle stabilization occurring after eight layers. Forsman et al.^148^ alternately deposited anionic carnauba wax dispersion with cationic poly-l-lysine (PLL) or starch, requiring two LBL cycles for optimal performance. The carnauba wax dispersion was prepared by adding carnauba wax to hot water at 90 °C. Afterward, it was sonicated, ice-cooled, and filtered. Curing temperature strongly affects the morphology, wetting behavior, and durability of carnauba-wax/biopolymer LBL coatings. Heating slightly above the wax melting point (≈85–95 °C) promotes wax flow and spreading, forming a more continuous hydrophobic layer with a higher WCA of ∼155° and improved wash resistance. Controlled cooling aids recrystallization, enhancing surface roughness and water repellency. However, excessive temperatures (>150 °C) may damage cellulose or biopolymers and reduce air permeability, while insufficient curing leads to incomplete film formation and poor durability. Arfaoui et al.^60–62^ treated TiO_2_-pretreated nonwoven samples with STA in ethanol, highlighting that even simplified LBL-like processes can impart hydrophobicity through sequential surface modification. Samples were dried at 100 °C for 10 min, resulting in a high WCA of ∼150°, indicating strong hydrophobicity. The coating also showed good durability, maintaining its water-repellent performance after several washing and abrasion cycles, confirming the stable chemical bonding between STA and the TiO_2_-modified surface.

##### 4.1.2.2 Coordination Self-Assembly (CO Self-Assembly)

It is a process in which predesigned molecular components spontaneously assemble into ordered structures via coordinate bonds between metal ions and organic or inorganic ligands.^142^ This approach allows for the creation of diverse architectures, ranging from simple complexes to intricate supramolecular structures.^142^ Branched ligands create cross-linked, defect-healing films with improved stiffness/dielectric behavior; swapping metal nodes or interleaving different ligands gives hybrid multilayers; “accelerated self-assembly”—evaporation/dewetting—cuts layer growth time from hours to minutes.^149^ Generally, CO self-assembly requires simple, solution-based equipment designed to ensure uniform film formation and controlled metal–ligand interaction.

CO self-assembly can be considered a sustainable method when it uses benign metals, renewable ligands, and green solvents under low-energy conditions.^142^ Conceptually, it is simple because it relies on the intrinsic “instructions” within the components themselves to form the final structure without complex external intervention.^142^ Unlike traditional covalent synthesis, which often requires multiple purification and reaction steps, a large, complex supramolecular structure is achieved simply by mixing the appropriate metal ions and ligands in solution.^142,149^ If using dipping or spraying for deposition, coatings are compatible with industrial setups. The cost of CO self-assembly can vary widely, from relatively low-cost and competitive for bioinspired coatings using commodity chemicals to prohibitively expensive when highly specialized, predesigned molecular ligands or noble metal ions are required.^81^ In its simplest “one-pot” form, CO self-assembly can be speedy (minutes to a few hours). However, when the same chemistry is used to build multilayer films or textile coatings, the process can become time-consuming—often requiring several hours or even overnight soaking for each organic-ligand. Coordination self-assembly is an energy-efficient technology. Its primary energy advantage stems from operating at mild temperatures and often eliminating the highly energy-intensive water heating and evaporation steps inherent in conventional textile wet processing.^150^ As many metal salts (e.g., Zn^2+^, Fe^3+^, Cu^2+^) and ligands (e.g., carboxylates, catechols, polyphenols) are water-soluble, the choice of water or solvent plays a crucial role in determining coordination efficiency, film morphology, and environmental impact. CO self-assembly and related coordination-based coating methods can be successfully applied to virtually any type of material, regardless of its inherent chemical nature, size, or shape, as long as an initial anchor point can be established.^145,33,151^ CO self-assembly used as an aqueous LbL finish generates the same “type” of wastes as other wet finishing processes—primarily rinse and bath effluents containing salts/organics—with the advantage that, when paired with low-wet-pickup application and green chemistries, total waste and energy can be substantially reduced compared with conventional practices.^24,150^

Gu et al.^152^ employed TA and Fe^3+^ ions to form a uniform metal–polyphenol complex on the fabric surface, followed by hydrophobization with 1-octadecylamine. The resulting coating exhibited a high WCA of approximately ∼153°, demonstrating excellent surface hydrophobicity. Moreover, the TA–Fe^3+^ interfacial network provided strong adhesion to the fabric fibers, contributing to good washing durability and mechanical stability. The hierarchical roughness generated by the metal–polyphenol complex, combined with the long alkyl chains of octadecylamine, resulted in a stable, water-repellent surface that did not significantly impact fabric flexibility or air permeability.

Similarly, Zhou et al.^151^ used PA and various metal ions (e.g., Fe^3+^, Zr^4+^) to construct micro/nanostructured surfaces via layer-by-layer deposition. The successive adsorption of metal–phytate complexes created a hierarchical roughness that is critical for water repellency. After assembly, the surfaces were modified with PDMS, introducing long hydrophobic chains that further lowered surface energy. The resulting fabrics exhibited high WCA (>150°) and demonstrated excellent durability, retaining their hydrophobicity even after multiple washing and abrasion cycles. Additionally, the metal–phytate network provided strong adhesion to the fibers, ensuring mechanical stability without compromising air permeability or fabric softness, demonstrating the effectiveness of combining metal–organic coordination with hydrophobic functionalization. While both approaches rely on the coordination between biobased ligands and metal ions to create functional coatings, Zhou et al.’s method focuses more on building surface roughness through hierarchical structuring, whereas Gu et al. emphasize uniform complexation followed by molecular surface modification. The overview of studies using CO self-assembly to create hydrophobicity is presented in Table 8.

**Table 8:** Natural-Based Hydrophobic Coatings Using the CO Self-Assembly Method: Used Methods and Reaction Mechanism, Textile Substrate and Coating Materials, Process Steps, Treatment Conditions, Hydrophobic Performance, and Durability

Methods used and reaction mechanisms	Textile substrate and coating materials	Process steps and treatment conditions	Hydrophobic performance and durability	ref
LBL self-assembly, coordination self-assembly	100% cellulosic fibers	1.0 Cleaning of the substrate,	WCA(PA-FeIII-PDMS) = 152 (±1)°, SA PA-FeIII-PDMS) = 13°, WCA (PDMS) = 114 (±2)°	^151^
Cross-linking, electrostatic interactions	PA, metal ions (AgI, FeIII, CeIII, ZrIV, and SnIV), PDMS	2.) immersion in PA solution, 2 min,	WCA (600 mesh sandpaper, 30 cycles) = 142 (±2.5)°, WCA (1000 mesh, 30 cycles) = 149 (±1.5)°, WCA (peeling test, 300 cycles) > 150°, SA (peeling test, 300 cycles) = 23°	
		3.) immersion in metal ion solution, 2 min,		
		4.) repeating steps 2 and 3,		
		5.) rinsing, drying at 80°,		
		6.) immersion in PDMS solution, 2 min,		
		7.) final curing at 80 °C for 2 h.		
Coordination assembly, dip-coating	cellulosic fibers	1.) Immersion in FeCl_3_, stirring at room T, 1 min,	WCA = 145.35 (±0,4)°	^152^
cross-linking, grafting	TA, Fe (III), ODA	2.) addition of TA,	WCA (standard machine laundry) = 141°, WCA (acid, alkali, neutral for 12 h) = 142°, WCA (heating at 200 °C for 1 h) = 142,14°	
		3.) pH adjustment, stirring at room T for 1h,		
		4.) washing in ethanol and drying under N_2_		

#### 4.1.3 Wet Topography Techniques

The roughened substrate, characterized by its peaks and valleys, provides mechanical interlocking points, enhancing not only hydrophobicity but also the adhesion of the polymer coating layer through improved surface anchoring.^130^

##### 4.1.3.1 Wet (chemical) Etching

It is a subtractive microfabrication or bulk micromachining method that involves the use of chemicals to selectively remove thin films or surface fibers, thereby removing layers from the bulk volume of the substrate.^153^ Chemical etching (Figure 9, Table 9) increases the surface roughness of fiber substrates. The substrate undergoes a thorough cleaning process to eliminate contaminants before the etching stage. Subsequently, specific regions of the substrate that require protection from etching are coated with a protective mask or resist material, such as photoresists, tapes, or stencils. The properly prepared substrate is then either immersed in or exposed to a carefully chosen chemical etchant solution that is capable of selectively dissolving the material.^154^ The selection of the appropriate etchant is critical and is determined by both the substrate’s composition and the desired etching characteristics. Typical etchants encompass acids, bases, or specialized chemical solutions tailored to the specific material. The etchant initiates a reaction with the exposed areas, resulting in the removal of material. Achieving the desired etch depth and profile requires precise control of parameters such as temperature, concentration, and etching duration. Following the etching stage, the substrate is thoroughly rinsed to eliminate any lingering etchant and neutralize its effects. Subsequently, upon removal of the protective mask, the substrate undergoes another cleaning process to remove any residues from the etching procedure, followed by thorough drying.^154–156^ The overall etch rate is governed by both the surface reaction kinetics and the transport of reactants and products within the boundary layer near the surface.^157^ For textile and superhydrophobic materials, etching is often performed at room temperature or low heat to protect the substrate or rely on highly reactive chemistries.^4,124^

**Figure 9:**
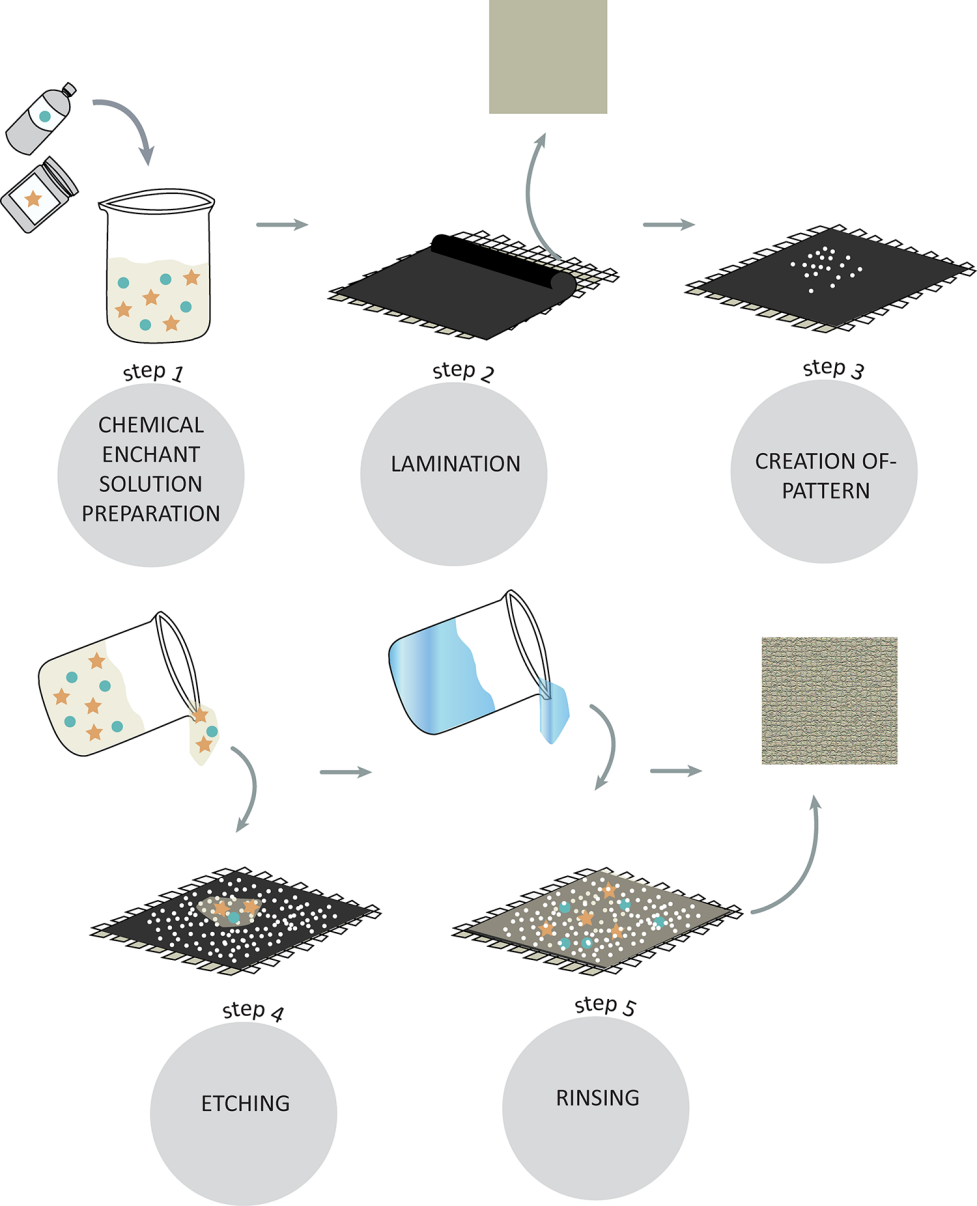
Schematic presentation of etching.: enchant solution preparation, lamination, creating pattern, etching, rinsing step.

**Table 9:** Natural-Based Hydrophobic Coatings Using the Wet Etching Technique: Used Methods and Reaction Mechanism, Textile Substrate and Coating Materials, Process Steps, Treatment Conditions, Hydrophobic Performance, and Durability

Methods used and reaction mechanisms	Textile substrate and coating materials	Process steps and treatment conditions	Hydrophobic performance and durability	ref
etching, dip-coating	100% cellulosic fibers	1.) Preparation of DES, heated to 80 °C under magnetic stirring,	WCA = 160 (±0.5)°	^38^
cross-linking	DES, CESO, STA	2.) preparation of cotton (washing),	WCA (1000 abrasion cycles) > 158, WCA (30 laundry washes) > 155°	
		3.) immersion in DES, 80 °C for 30 min, stirring,		
		4.) rinsing,		
		5.) immersion in CESO, STA solution for 5 min, withdrawal at control speed of 5 min,		
		6.) air-drying for 30 min,		
		7.) curing at 120 °C for 2 h,		
		8.) rinsing,		
		9.) drying at 60 °C for 30 min.		
etching, dip coating	100% cellulosic fibers	1.) Preparation of solutions,	WCA = 156 (±1)°	^4^
cross-linking	PA, ESO, STA	2.) immersion in PA solution for 20 min, stirring,	WCA (1200 abrasion cycles) > 155°, WCA (30 laundry cycles) = 155°, WCA (immersion in acid, alkali and neutral) > 150, WCA (heating at 100 °C for 6 h) = 154°	
		3.) washing in deionized water 3 times, 30 min,		
		4.) air drying at 100 °C, 2 h,		
		5.) precuring of fabric heated at 60 °C for 30 min,		
		6.) curing at 150 °C for 2 h,		
		7.) immersion in STA, ethanol solution for 6 h,		
		8.) curing at 80 °C for 30 min,		
		9.) washing with ethanol,		
		10.) drying at 100 °C for 2 h.		
etching, chemical vapor deposition (MTCS)	100% silk, 100% wool (only treatment with alkaline protease)	1.) Cleaning of fabric with ethanol and deionized water,	WCA (PA+MTCS) = 154°, WCA (MTCS) = 110°, WCA (PA, alkaline protease) = 157°, WCA (alkaline protease on wool) = 152°	^37^
silanization	papain (papaya enzyme), alkaline protease, methyltrichlorosilane (MTCS)	2.) immersion of silk in a water bath, 70 °C,	WCA (100 abrasion cycles) = 154°, SA = 16°, WCA (10 domestic cycles) = 150°	
		3.) washing in deionized water,		
		4.) drying at 80 °C,		
		5.) immersion in MTCS vapor for 10 h at 66 °C.		

Compared to dry etching (Section 4.2.2.1), wet etching exhibits a smoother surface, fewer etch-induced defects, a reasonable etch rate, and better selectivity.^157^ In terms of simplicity, wet etching is generally considered simpler than dry etching, requiring less sophisticated equipment. Its energy consumption is considered low to moderate in terms of direct energy input compared to dry washing. Still, the overall energy footprint of the process can increase due to auxiliary needs, such as heating and water purification.^157^ Cost-wise, it is typically more cost-effective due to lower equipment and operational expenses.^99^ However, wet etching consumes significant water for rinsing and dilution.^158^ The etching time can vary, and it may not achieve high aspect ratios due to its isotropic nature. Wet etching consumes chemicals and generates liquid waste that needs proper disposal, impacting its environmental footprint. The biodegradability of wet-etched textiles hinges on postetch finishes; some common antimicrobials severely inhibit the biodegradation of shed fibers, making finish selection and testing critical.^159,160^ While the initial setup may require some preparation time, certain wet chemical reaction techniques are attractive due to their low cost and large-scale production capacity.^58^ Wet etching is often preferred when high selectivity and minimal surface damage are critical. The technique can be applied to a wide range of textile substrates, including both natural and synthetic fibers. However, the process can be difficult to control, and achieving a uniform etch across the textile substrate can be challenging.^99,153,157^ Properly engineered wet-etched textiles—using gentle etchants to sculpt durable roughness and covalently anchored hydrophobes—can achieve long-lasting superhydrophobicity with excellent resistance to abrasion, laundering, solvents, temperature, and pH, while maintaining breathability and acceptable mechanical properties. In biobased coatings, natural enzymes and polyphenols, such as PA and tannins, are utilized. Specifically, PA serves as a robust chemical etchant to induce microscale surface roughness on cellulosic fibers, a prerequisite for subsequent hydrophobic modification. Concurrently, hydrolytic enzymes such as cellulose or protease are frequently used for precise biological etching, creating the delicate hierarchical structures essential for achieving the necessary roughness and ultimately securing a stable superhydrophobic state.^92,117^ Xu et al.^4^ utilized natural PA to etch cellulosic fibers by immersing the fabric in a PA solution for 20 min, followed by rinsing with deionized water and drying at 100 °C for 2 h. When subsequently coated with a low-surface-energy layer of CESO and STA, the final superhydrophobic cotton fabric achieved a high WCA of ∼156°. This modification preserved the inherent fabric properties, such as water vapor permeability and flexibility. The coating demonstrated excellent durability, maintaining WCA ∼ 155° after 1200 abrasion cycles and retaining ∼154° after 30 laundering cycles, along with outstanding chemical and thermal durability. In another study, the same group used a deep eutectic solvent (DES), prepared by heating a mixture of choline chloride and oxalic acid dihydrate at 110 °C for 2 h. Cellulosic fibers were then immersed in the DES at 60 °C for 2 h, rinsed with distilled water, and dried at 100 °C for 1 h.^38^ The DES treatment, being milder than traditional etching chemicals, was reported to nearly preserve the fabric’s mechanical strength. When subsequently coated with CESO and STA, this fabric achieved an even higher WCA of ∼159.7°. This coating exhibited excellent mechanical stability, enduring 1000 abrasion cycles and 30 laundering cycles while maintaining a WCA above 154.6°. Furthermore, it demonstrated strong thermal and chemical endurance, supporting its application in self-cleaning and high-efficiency oil–water separation.

Şahan and Demir et al.^161^ treated wool with protease as a pretreatment for hydrophobic modification. After enzyme application, the fabrics were rinsed at 60 °C and pH 4 for 10 min, followed by repeated rinsing with deionized water to remove residual enzymes. Following enzymatic etching to achieve surface roughness, the treated fabric was modified via a thermal chemical vapor deposition process at 70 °C to impart low surface energy. This two-step approach achieved WCA ∼ 152°. The resulting coating demonstrated excellent self-cleaning abilities and maintained a sustained high level of hydrophobicity after multiple laundering cycles. Similarly, Cheng et al.^92^ etched silk using papain, a natural enzyme from papaya, dissolved in deionized water. The fabric was treated at 70 °C and pH 7 for 30 min. This process created a rugged surface with trenches, improving WCA from ∼110° to 153.5°, and demonstrating durable surface roughness even after abrasion and pH variations. However, the tensile strength of silk fibroin decreased as the enzyme concentration and treatment time increased.

##### 4.1.3.2 Nanoparticle-Based Techniques

Another way to achieve surface roughness is by depositing NPS. NPS are defined as ultrafine particles whose dimensions typically lie between 1 nm and 100 nm. This nanoscale dimension results in unique physical and chemical characteristics, including a significantly high surface area-to-volume ratio, which makes their properties dependent on their size, shape, and structure.^162^ NPS can be grouped by composition (inorganic, polymeric, lipid-based, biological) and by dimensionality (0-D spheres, 1-D rods/wires, 2-D sheets, 3-D assemblies). The shape (Figure 10) and morphology—whether spherical, rod-like, cubic, star-shaped, core–shell, or hierarchical aggregates—strongly influence functional properties such as optical resonance, catalytic activity, and wettability.^162–164^

**Figure 10:**
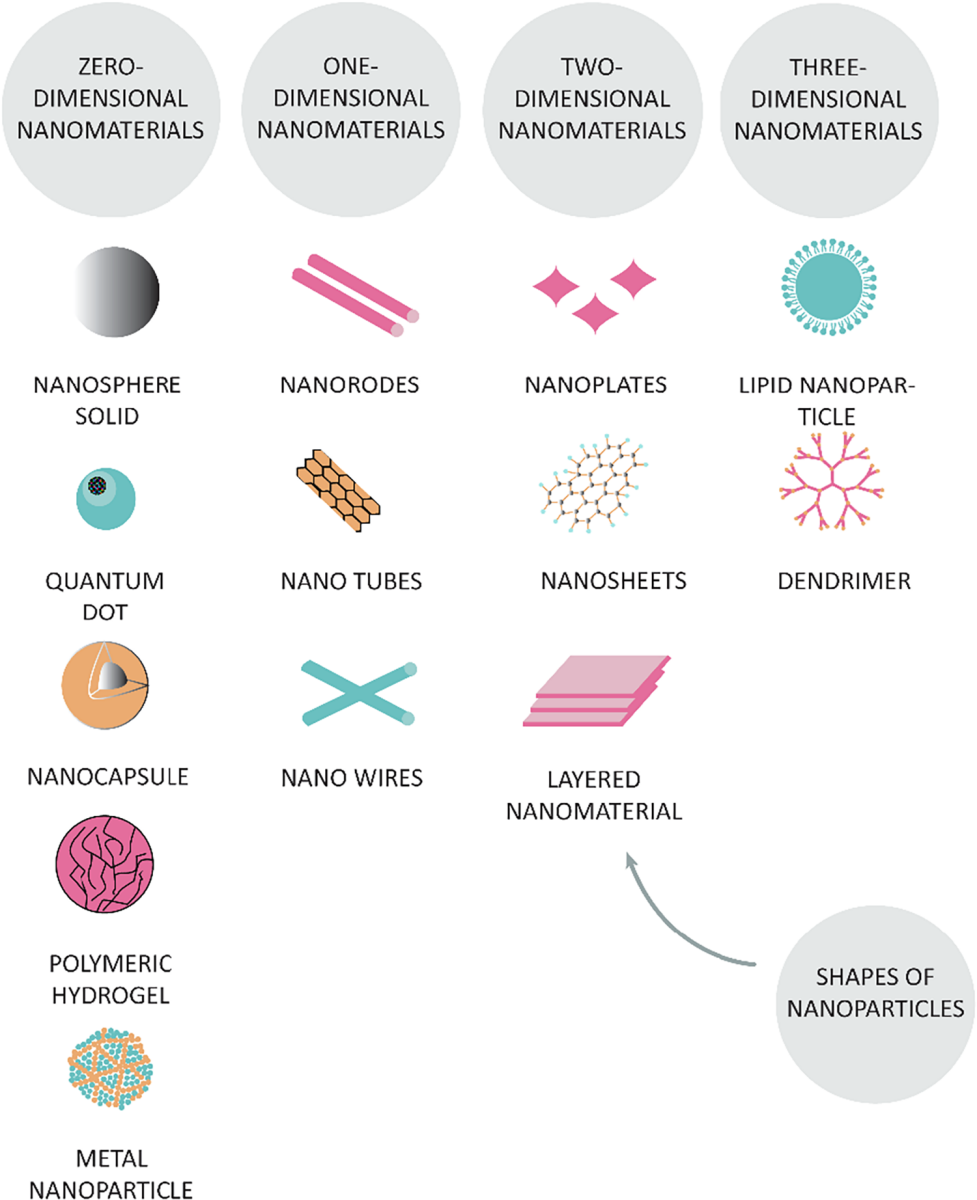
Schematic presentation of nanoparticle shapes: nanosphere, quantum dot, nanocapsule, polymeric hydrogel, metal NPS, nanorodes, nanotubes, nanowires, nanoplates, nanosheets, layered nanomaterial, lipid nanoparticle, dendrimer.

NPS techniques have varied sustainability implications, as some traditional ingredients, like halogenated compounds, are toxic and pose environmental issues.^32^ Under REACH, nanomaterials have to have nanospecific information requirements. European Chemicals Agency (ECHA) expects detailed physicochemical characterizations, exposure and release potential, and risk assessments (including for coated products). When textiles incorporate nanomaterials, manufacturers should anticipate requests for data on nanoparticles (NPS) identity, size distribution, surface treatment, durability (release), and worker/consumer exposure.^129^ Under EU REACH, any textile article that contains a substance of very high concern (SVHC) at ≥ 0.1 % w/w must be communicated to downstream recipients and, where required, to consumers.^165^ If a hydrophobic finish also provides antimicrobial or biocidal activity (e.g., silver NPS), the active ingredient must be authorized under the EU Biocidal Products Regulation (or equivalent national rules) and only approved claims may be used.^14,129,166^ LCA is crucial for evaluating the environmental impact of nanoparticle-based textiles. These assessments consider factors from design and material choices to end-of-life recyclability. Addressing the durability and potential leaching of NPS is essential for minimizing environmental and health risks.^162^ Employing eco-design principles and shifting toward nonmetallic, biobased compounds can further enhance sustainability.^100^ Examples of NPS that were already used for hydrophobic coatings on natural textiles are zein NPS,^88^ carnauba wax NPS,^36^ CNF,^40^ and CNC.^124,80^

The importance of NPS stems from their unique combination of physicochemical properties and superior environmental benefits, positioning them as fundamental components in developing sustainable functional materials. There are two general groups for the synthesis of NPS. Bottom-up and bottom-down approaches. Bottom-up approaches to nanoparticle synthesis involve assembling nanostructures from smaller components, such as atoms or molecules.^167^ These approaches offer exceptional control over material composition, enabling the incorporation of various functionalities, including those that rely on self-assembly processes.^67^ Common routes include the sol–gel processes.^168^ As this is the most widely used technique for nanoparticle synthesis, it will be discussed in a separate chapter. Another commonly used method for NPS, which has also been applied in combination with naturally based coatings, is nanoseeding. This technique is used to initiate and control the growth of nanomaterials—typically NPS, nanocrystals, or nanostructured films—by introducing small “seed” particles that act as nucleation sites.^145,164^ Arfaoui et al.^76^ treated scoured jute fibers by immersing them in a ZnO nanoseed solution (zinc acetate and NaOH in ethanol) for 15 min, followed by drying at 120 °C for 15 min. This cycle was repeated four times to ensure proper seed fixation. The ZnO-seeded fibers were then immersed in a solution of hexamethylenetetramine (HMTA) and zinc nitrate at 95 °C for 5 h to grow ZnO nanorods. After rinsing and drying, the fibers were dipped in a stearic acid solution in ethanol for 3 h and dried at 100 °C for 15 min. The stearic acid modified the ZnO-coated surface, lowering its surface energy and, together with the roughness provided by the ZnO nanorods, imparting superhydrophobicity.

Bottom-down nanoparticle synthesis starts from bulk material and reduces it to the nanoscale through destructive or decompositional processes.^67^ Typical methods include high-energy mechanical milling/attrition, where prolonged grinding (e.g., of coconut-shell precursors) progressively shrinks crystallite size, and chemical or plasma etching, which selectively removes material with acids or with ionized gases to create rough micro/nano-features on substrates.^162,169^

##### 4.1.3.3 Sol–Gel

The sol–gel process (Figure 11, Table 10) is used to create inorganic or hybrid organic–inorganic materials. It involves the transformation of a solution (sol) into a solid or gel-like material through a series of chemical reactions, typically at relatively low temperatures. The process starts with the hydrolysis of precursor compounds (metal alkoxides or metal salts) in a suitable solvent (usually an alcohol like ethanol or methanol).^168^ Hydrolysis involves the reaction of the precursor with water to form metal hydroxides or oxides. As hydrolysis continues, the solution begins to transform into a gel-like state. Polymerization reactions occur, leading to the formation of a three-dimensional network of interconnected particles or chains. The gel is allowed to age, during which further reactions occur, such as syneresis, further condensation, and dissolution–reprecipitation.^170,171^ After the desired gel structure is achieved, the solvent is gradually removed through drying. This step involves evaporation or controlled heating to eliminate the liquid, leaving behind a porous solid.^168,172^ Thin-film coatings are achieved by dipping, padding, spinning, or spraying the substrate with a sol. After deposition, controlled drying and curing (often at temperatures below ∼130 °C) consolidate the gel (via further condensation) and remove the solvent, forming adherent thin films or particle-decorated surfaces.^173^ The sol–gel process allows precise control over the composition, porosity, and structure of the resulting materials.^168^ Sol–gel coatings can be highly homogeneous at the molecular level, leading to superior purity and uniformity in the final material.^168^ The technique allows the formation of new crystalline phases from noncrystalline solids.

**Figure 11:**
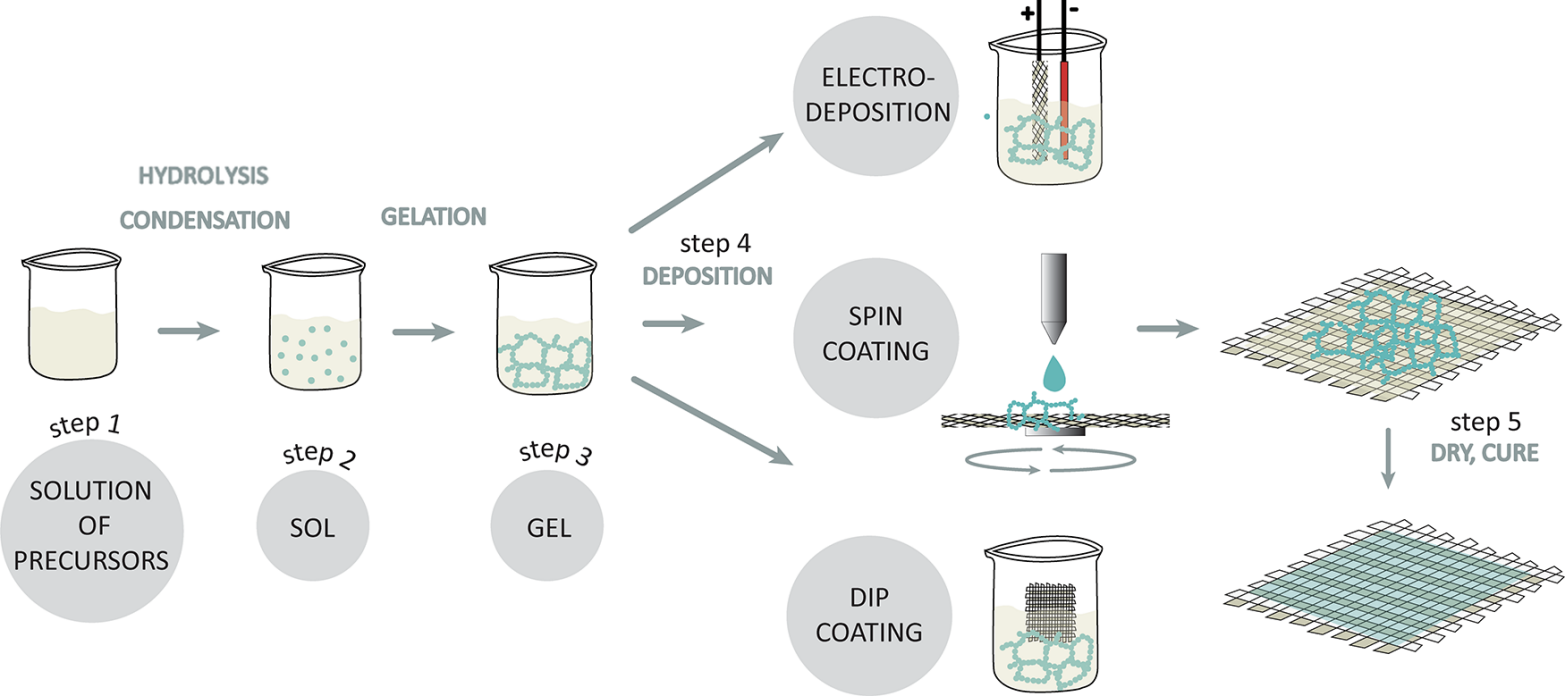
Schematic presentation of sol–gel: preparation of a solution of precursors, hydrolysis condensation into sol, gelation, deposition (electrodeposition, spin coating or dip coating), drying, curing.

**Table 10:** Natural-Based Hydrophobic Coatings Using the Sol-Gel Technique: Used Methods and Reaction Mechanism, Textile Substrate and Coating Materials, Process Steps, and Treatment Conditions, Hydrophobic Performance and Durability

Methods used and reaction mechanisms	Textile substrate and coating materials	Process steps and treatment conditions	Hydrophobic performance and durability	ref
sol–gel (TiO_2_ solution preparation), dip-pad-dry	100% jute fabric	1.) TiO_2_ nanoparticle synthesis via sol gel (stirring at room T 6 h),	WCA (TiO_2_+STA) = 135 (±3)°, WCA (STA) = 127 (±5)°	^60–62^
hydrolysis, condensation	TiO_2_, STA	2.) immersion in nanoparticle solution at room T,		
		3.) drying at 100 °C, 10 min,		
		4.) rinsing with ethanol,		
		5.) drying at 100 °C for 10 min,		
		6.) immersion in STA solution for few seconds,		
		7.) drying at 100 °C for 10 min.		
sol–gel (for preparation of nano fibrils), spraying	100% cellulosic fibers	1.) Preparation of peanut shell powder NPS(extraction, air drying, lignin removal 2×, washing, hemicellulose removal, mechanical processing),	WCA = 160°	^66^
oxidation (pretreatment), silanization	CNF (from peanut shell powder), MTMS, PMDS	2.) preparation of CNF- aerogel (addition of MTMS solution),		
		3.) spraying fabric with CNF-aerogel,		
		4.) drying for 5 min		
		5.) spraying of PDMS,		
		6.) drying in an oven at 120 ° for 60 min.		
sol–gel (silica), pad-dip dry	cellulosic fibers	1.) Preparation of silica sols,	Sink in time = 35 s	^125^
electrostatic interactions, hydrogen bonding, dipole–dipole interactions	CS, silica sol	2.) preparation of shitosan solutions, stirring,		
		3.) padding		
		4.) drying at 120 °C for 3 min.		
pad-dry, sol–gel	100% weaved wool, 100% knitted and weaved cotton	1.) Synthesis of coating solution,	WCA = 135°	^176^
electrostatic interaction, grafting	Phase-transited lysozyme (PTL), TA, 3-mercaptopropyltriethoxysilane (MPTES), dimethyloctadecyl[3-(trimethoxysilyl)propyl]ammonium chloride (DTSAC)	2.) pretreatment of wool in aqueous solution for 3 h at 60 °C,		
		3.) pretreatment of cotton in NaOH solution for 1 h, 70 °C,		
		4.) padding, 2 dips and 2 rollings,		
		5.) drying/curing at 40 °C,		
		6.) immersion in MPTES solution for 12 h at 40 °C		
		7.) addition of DTSAC, stirred for 4 h at 25 °C,		
		8.) washing in ethanol		
		9.) drying 30 min at 120 °C,		
		10.) electrically conductive modification.		
sol–gel, dip-pad-dry	100% silk	1.) Preparation of PA-doped sol, stirring,	WCA (PA) = 83°, WCA (APDTM) = 114°, WCA (PA + APDTM) = 130°	^92^
cross-linking	PA+TEOS+ethanol, APDTM	2.) preparation of silk (scouring),	N/A	
		3.) immersion in sol solution, 10 min, room temperature,		
		4.) padding, teo dips, two nips,		
		5.) drying in an oven at 80 °C,		
		6.) curing at 160 °C for 3 min.		
		7.) rinsing with cold distilled water,		
		8.) dried at room temperature.		

The sol–gel technique is widely considered a simple, versatile, and facile method.^67^ The method can be used with various precursor types, often eliminating the need for expensive or sophisticated equipment.^58^ The process is considered a low-cost and sustainable method for creating inorganic and hybrid materials. Sol–gel is generally a low-energy route compared with conventional melt/solid-state processing because most chemistry (hydrolysis/condensation, gelation) occurs at or near ambient temperature, and many coatings only require low-temperature drying/curing (typically 80–140 °C for tens of minutes to ∼1 h).^171^ However, it involves individual steps and is time-consuming, making the cost and complexity of achieving uniform coatings on large-scale textiles a significant challenge for practical application.^129^ The technique is characterized by less chemical consumption, which can lead to reduced waste. While the reactions typically rely on water and/or organic solvents for hydrolysis, the overall process requires far less water than traditional aqueous dyeing and finishing.^70^ Conventional sol–gel finishes on textiles are predominantly inorganic (silica-, titania-, or zirconia-based) or inorganic–organic hybrids. Consequently, they are not biodegradable in the sense of compostability commonly applied to plastics. On cotton substrates, the cellulose fibers can biodegrade; however, the thin mineral-like sol–gel residue remains as amorphous SiO_2_ or other oxides, which are generally inert.^19,174,129^ That is why it is essential to use nature-based NPS, which can safely biodegrade in the environment.^175^ Sustainability challenges also include the high cost of precursor materials and issues such as shrinkage and cracking during the drying process.^172,173^ Properly engineered sol–gel finishes (good bonding, controlled thickness, and robust low-energy topcoats) can be highly durable, withstanding hundreds to thousands of abrasion cycles, dozens of washing cycles, week-long solvent immersion, and UV/thermal exposure, while maintaining high water repellency and functional performance on textiles and other porous substrates.^151^ Chen et al.^66^ prepared cellulose nanofibrils (CNCs) from peanut shell powder, highlighting an eco-friendly approach to valorize agricultural waste. The peanut shells were first processed into NPS through extraction, air drying, lignin removal (twice), washing, hemicellulose removal, and mechanical treatment, yielding high-purity cellulose NPSs. These NPS were then transformed into CNF aerogels via a sol–gel approach, using MTMS to promote silanization and stabilize the nanofibril network. The CNF-aerogel was sprayed onto 100% cellulosic fabrics, followed by a 5 min drying period, and a second spray coating with PDMS was applied. The final fabrics were oven-dried at 120 °C for 60 min, resulting in a superhydrophobic surface with a WCA of ∼160°. This multistep approach, which combines oxidation pretreatment, sol–gel–assisted nanofibril formation, and sequential spraying, demonstrates a scalable and sustainable strategy for producing durable, high-performance hydrophobic coatings from agricultural byproducts. Cheng et al.^92^ 100% silk fabrics were functionalized using a sol–gel dip–pad–dry method to enhance hydrophobicity. The PA–doped sol was prepared by stirring a mixture of PA, TEOS, and ethanol, while APDTM was used as a cross-linking agent in selected samples. The silk fabrics were first scoured and then immersed in the sol solution at room temperature for 10 min. This was followed by padding with two dips and two nips to ensure a uniform coating. The fabrics were subsequently dried in an oven at 80 °C, cured at 160 °C for 3 min, rinsed with cold distilled water, and air-dried at room temperature. The WCA measurements indicated that PA alone imparted a WCA of ∼83°, 3-aminopropyldimethoxymethylsilane (APDTM) alone achieved ∼114°, and the combination of PA and APDTM enhanced hydrophobicity to 130°, demonstrating a synergistic effect of the sol–gel components and cross-linking in producing moderately hydrophobic silk surfaces.

For the deposition of NPS (Figure 12, Table 11), traditional solution-based deposition techniques, as described in Section 4.1.1, are commonly used.

**Figure 12:**
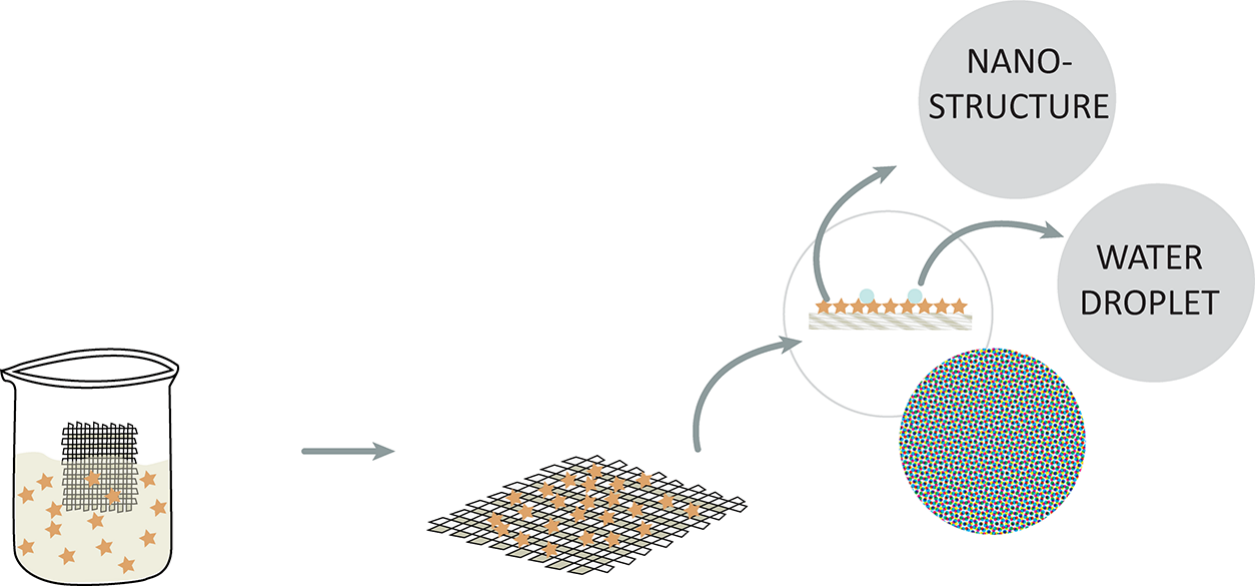
Schematic presentation of nanoparticle’s deposition.

**Table 11:** NPS Used in Natural-Based Hydrophobic Coating: Nanoparticles Used, Synthesis Technique, Deposition Technique, Size and Shape^a^

NPS used	Synthesis technique	Deposition technique	Nanoparticle’s size	Nanoparticle’s shape	ref
ZnO	nanoseeding/nanorod treatment	dip coating	40 ± 44 nm	nanorod with hexagonal shape	^76^
TiO_2_, ZnO	nanoseeding, growth of nanostructures	dip-pad-dry	TiO_2_: 13 ± 6 nm	nanorods with a hexagonal truncated pyramidal shape	^126^
		SA, ZnO nanoparticles, TiO_2_	ZnO: 40 ± 44 nm,		
CNF	2,2,6,6-tetrametilpiperidin-1-oksil (TEMPO) oxidation, aqueous counter collision (ACC) treatment	spraying	N/A	N/A	^40^
ZnO	commercially available	spray coating	50 ± 10 nm	N/A	^98^
carnauba wax NPS	solvent-free emulsion/melt dispersion technique	LBl self-assembly technique	80–200 nm	cubic shape	^36^
CNC	TEMPO oxidation and mechanical homogenization	PDC	Diameter: 10–25 nm, length: 200–400 nm	N/A	^80^
zein NPS	N/A	drop-casting, dip-pad	380–450 nm	N/A	^88^
CNC	acidolysis of cellulosic fiber pulp	dip-coating	Diameter 7–10 nm, length: 200 nm	N/A	^110^
78TiO_2_	commercially available	dip-coating	N/A	N/A	^26^
TiO_2_	commercially available	dip-coating	60 nm	N/A	^82^
natural rubber NPS, gold NPS	in situ synthesis of AuNP within the NRL matrix	dip-coating	0.5–3 um	spherical shape	^62^
silver NPS	chemical reduction method	dip-coating	20–30 nm	core–shell shape	^87^
TiO_2_	sol gel	dip-pad-dry	varies depending on the ethanol concentration: 13 ± 6 nm, 83 ± 51 nm, 837 ± 279 nm	N/A	^60–62^
CNC (from peanut shell)	sol–gel	spraying	10 to 30 nm	hollow-rod shape	^66^
silica NPS	sol–gel	pad-dip dry	N/A	N/A	^125^

a N/A indicates that data in not available.

### 4.2 Dry Methods

#### 4.2.1 Dry Functionalization Techniques

##### 4.2.1.1 Plasma Treatment

Plasma-based textile treatment (schematically presented in Figure 13, Table 12) stands as a revolutionary approach for altering both the physical and chemical properties of a surface.^109,177^ It is often referred to as the fourth state of matter. It is a gas that is partially ionized. Khelifa et al.^178^ describes plasma as “a quasi-neutral gas of charged and neutral particles characterized by a collective behavior”. When subjected to a continuous increase in temperature, a solid material undergoes sequential transitions to liquid and gaseous states.^178^ When material comes into contact with the plasma, the surface is bombarded by a range of plasma particles (including electrons, ions, radicals, and neutrals) and UV photons, each impacting the surface with a broad energy distribution.^179^

**Figure 13:**
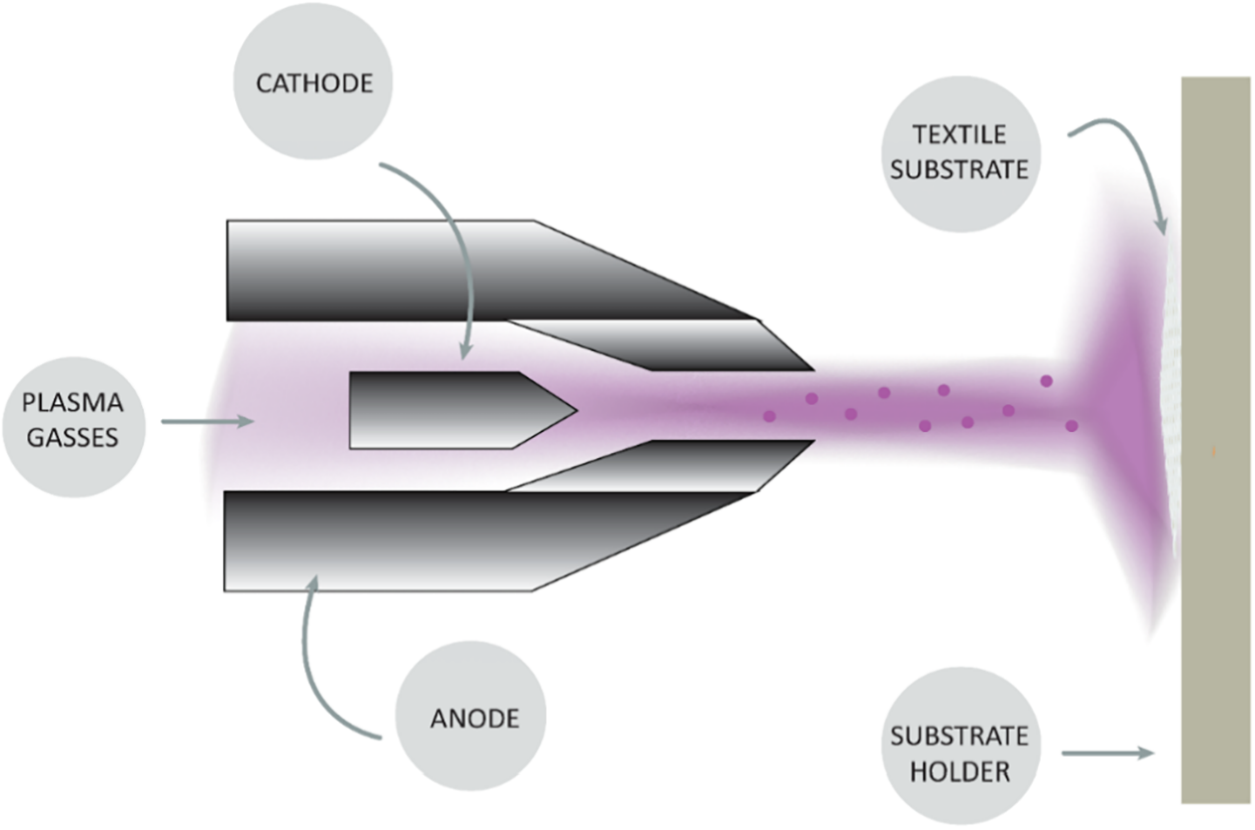
Plasma treatment: a gas is introduced into the chamber and ionized by a high-frequency power source, resulting in the generation of plasma.

**Table 12:** Nature-Based Hydrophobic Coatings Using Plasma: Type of Plasma, Plasma Conditions, Textile Substrate, Post-treatment Methods and Their Conditions, Materials

Type of plasma	Plasma conditions	Textile substrate	Post-treatment method and its conditions	Materials	ref
atmospheric plasma	gas: O_2_ (frequency: 25 kHz, power 2.5 kW, treatment speed 1m min^–1^, 6 times)	100% cellulosic fibers	LBL-self-assembly, pad-dry-cure (immersion for 5 min, 100% pick up, oven drying at 70° for 15 min after each immersion)	carnauba wax NPS, ZnO NPS	^36^
low-pressure plasma	microwave plasmareactor; generator frequency: 2.45 GHz, discharge power:500W, exposure time 240 s, Ar flow rate; 60 mL/min, pressure 25 Pa)	100% cellulosic fibers	dip-coating (immersion, air-drying)	oleic acid	^60^
atmospheric plasma	microwave plasma, 200 W, 2 min	100% cellulosic fibers	spraying (drying at 60 °C, 30 min)	CNF (from hardwood pulp), ODA, glutaraldehyde	^40^

Plasmas can be categorized as either hot/thermal (equilibrium) plasma or cold/nonthermal (nonequilibrium) plasma, depending on the temperature of the plasma zone.^179^ In thermal plasmas, temperatures range from 800 to 9700 °C, and are used in applications like welding and coating. Nonthermal plasmas are cool, with hot electrons (9700 to 49700 °C) but ions and neutrals near room temperature.^109^ The high-energy electrons efficiently transfer energy through inelastic collisions to feedstock gas molecules, causing dissociation and fragmentation to yield chemically reactive species that are far from thermodynamic equilibrium.^34^ The mechanism involves concurrent processes such as surface cleaning (ablation/etching of organic contaminants via ion/radical bombardment),^34,158^ bond scission and cross-linking in the polymer surface,^34,158^ and the covalent grafting of functional groups (e.g., hydroxyl—OH, carboxyl—COOH, and amino—NH) through free-radical chemistry, thus modifying the surface energy, wettability, and adhesion without affecting the material’s bulk properties.^34,180^ Low-pressure (vacuum) reactors and atmospheric-pressure systems both exist; the former offer highly uniform, controllable treatments, while the latter integrate in-line more easily with textile finishing lines.^34^ In the low-pressure plasma process, a vacuum chamber is first evacuated to a pressure of around 10^–2^–10^–3^ mbar using high-vacuum pumps. A gas is then introduced into the chamber and ionized by a high-frequency power source, generating plasma. Atmospheric plasma systems operate without vacuum equipment, enabling continuous, high-speed processing that is compatible with industrial textile manufacturing. Ambient air is commonly used as the gas source, making these methods practical and cost-effective. Corona discharge produces bright, filament-like plasmas between a sharp, high-voltage electrode and the moving textile. It is the most established atmospheric plasma technique; however, the treatment is mainly superficial and short-lived. There is limited penetration into yarns or densely woven fabrics. It requires tiny electrode gaps (∼1 mm) and precise positioning. Not ideal for thick or structured materials. Dielectric barrier discharge uses insulating barriers on one or both electrodes and high-voltage alternating current. It produces many small, cold microarcs, enabling atmospheric-pressure operation and better energy distribution than corona. However, microdischarges can be nonuniform, leading to uneven treatment across the textile width.^34^ Atmospheric pressure glow discharge (APGD) generates a homogeneous, stable, nonthermal glow plasma, often with gases such as helium or argon. It combines uniform activation, similar to low-pressure plasma, with no vacuum chamber required. Therefore, APGD offers a stronger and more controlled atmospheric plasma option for functionalizing fabrics and nonwovens (e.g., hydrophilicity, adhesion, dyeability).^34^

The simplicity of plasma treatment depends on the specific type of plasma system used and the complexity of the desired surface modification. It usually requires a trained operator or specialist to set up, run, and maintain the process safely and reproducibly. The method is time-efficient as it often involves fewer steps^181^ and short treatment times.^109,158^ It is a highly controllable and versatile alternative to traditional wet chemical finishing methods.^150,182^ It offers multiple benefits, including operation at low temperatures, which reduces the risk of fabric degradation. Its adaptability to a wide range of thermal, physical, and chemical conditions enables tailored treatments for specific textile properties.

Plasma pretreatments can reduce water and chemical use, as well as effluent (e.g., when coupled with enzymatic steps), thereby lowering life-cycle burdens at the preparation/finishing stages.^24,150^ The management of waste gas from plasma treatments is crucial for environmental compliance and economic efficiency, relying on closed systems that minimize releases. Gases are managed by type: expensive inert gases like argon and helium are often recycled with recirculation devices, significantly reducing consumption. Effluents generated during plasma etching or cleaning, which contain volatile contaminants and sometimes water vapor, are typically evacuated by vacuum pumps and filtered using traps. For systems using reactive gases (like oxygen) or air, hazardous byproducts such as ozone must be actively destroyed using catalytic converters before venting. Specialized hardware, including adsorption columns and catalytic destruction units, is integrated to ensure that unreacted precursors, solvents, and pollutants are contained or converted into safe forms, providing a key environmental advantage over conventional wet processes.^34,180,183^ The production cost can be relatively low due to reduced consumption of chemicals and water.^183^ However, plasma treatment has notable limitations. Since it primarily modifies the surface layer, contaminants or inconsistencies on the textile surface (e.g., differences between weft and warp directions) can lead to uneven results. Additionally, plasma species may not penetrate the porous, three-dimensional structure of textiles as effectively as wet processes do. The effects of plasma treatment are often not permanent, and its energy-intensive nature increases operational costs.^179^ However, Kan et al.^150^ reported that plasma treatment uses approximately 9.8 mL of gasoline per meter of fabric, whereas a conventional pretreatment method requires around 62.5 mL per meter. The technology’s efficiency depends heavily on precise system parameters, yet achieving uniform reaction conditions can be challenging even with controlled flow rate, gas pressure, and power input. Plasma technology has made significant advancements, making it viable for industrial-scale applications.^60^ However, the high cost of plasma equipment and the technical complexities of scaling up from pilot to continuous processes remain significant barriers. Structural overlaps in fibers or high-twist yarns exacerbate these challenges, creating shadow effects that shield areas from effective plasma exposure.^183^ Plasma also enhances adhesion and provides sterilization capabilities, a key advantage for natural fibers that are susceptible to bacterial growth.^184^

Plasma processes can be employed in preparing materials for hydrophobic textile treatments. Plasma can pretreat or post-treat surfaces already coated using wet chemical methods, employing precursor-free plasmas in gas mixtures. This approach modifies textile surfaces, creating a rough, hydrophilic surface that enhances the adhesion of subsequent hydrophobic coatings, thereby reducing surface energies.^109^ Plasma can also be effectively utilized for surface cleaning. It removes contaminants, grease, and other unwanted substances from the material surface, ensuring better adhesion and performance of hydrophobic treatments. In the natural-based (fluorine-free) studies, plasma is most often used for surface activation. Plasma can activate the textile surface by introducing polar functional groups and increasing surface energy, thereby enhancing coating adhesion and uniformity without altering the bulk properties of the material. Bashari et al.^36^ used oxygen pretreatment on cellulosic fibers using an atmospheric plasma machine. The machine operated at a frequency of 25 kHz and a power of 2.5 kW, with a treatment speed of 1 m/min. This process was repeated six times to ensure adequate surface modification. After the plasma treatment, the specimens were sealed in a bag at ambient temperature to prevent contamination and maintain the activated surface. This pretreatment, followed by carnauba wax and ZnO nanoparticle coating, achieved a high hydrophobic WCA of ∼131° with water droplets remaining on the surface for over three minutes without spreading. Crucially, this plasma-induced adhesion conferred durability, as the WCA remained hydrophobic ∼ 130° even after washing. Plasma grafting involves using mild plasma conditions, such as low plasma power or pulsed plasma techniques, to retain the chemical structure of precursors. Free radicals are created on the polymer surface using inert gas plasma treatment. These radicals then react with monomer gases introduced into the treatment chamber. This technique can lead to a thin-film coating on substrates.^109^ By this method, a layer with unique functions can be deposited.^185^ Cabrales et al.^60^ enhanced cotton’s water repellency by plasma-induced grafting of oleic acid using microwave plasma treatment with argon gas. The fabric was treated for 240 s at 500 W, 60 mL/min flow rate, and 25 Pa pressure to generate surface radicals, followed by immersion in oleic acid solution. Higher oleic acid concentrations led to more copolymer formation and reduced grafting efficiency. A second round of the 240 s plasma treatment completed the process. Optimal grafting was achieved at oleic acid concentrations between 0.1 and 0.15 mol/L, resulting in superhydrophobic fabrics with WCA exceeding 150°. Concentrations higher than 0.15 mol/L led to detrimental homopolymerization, reducing grafting efficiency; however, the optimal conditions successfully demonstrated a clean, cold-process method using a renewable, plant-derived monomer to impart durable water repellency. Roy et al.^81^ grafted octadecylamine onto cationic CS after oxygen plasma treatment of cellulosic fibers to improve coating adhesion. CS was dissolved in acetic acid, ODA in ethanol, and Fourier Transform Infrared Spectroscopy (FTIR) confirmed the bonding of ODA and glutaraldehyde through characteristic alkyl chain peaks. The application of this CS-ODA (CS-ODA) coating resulted in a superhydrophobic surface on the cotton fabric, achieving a high WCA of 161°.

Plasma treatment can also modify the surface of materials through etching^150^ as described in Section 4.2.2.1.

#### 4.2.2 Dry Topography Techniques

##### 4.2.2.1 Dry Etching

Dry etching is typically an anisotropic process in which ion species, propelled toward the substrate by momentum, in combination with a masking process, are used to physically remove the target materials. Dry etching offers better controllability and anisotropic etching, making it suitable for high-aspect-ratio features.^153^ It uses vapor-phase etchants, often involving plasma-assisted chemical reactions or energetic ion beams to remove material. Dry etching usually has a higher etch rate than wet etching. However, dry etching can cause ion-induced damage, exacerbating surface roughness.^21,34,157,158^ Dry etching needs a vacuum chamber to ensure controllability and reproducibility. It requires high-density plasma etch techniques, such as inductively coupled plasma, which can enhance efficiency. Dry etching produces less wastewater, reduces wastewater treatment costs, and is faster compared to wet etching. Dry etching is an energy-efficient process that requires minimal chemicals and generates no downstream pollution.^21,34,157,158^

##### 4.2.2.2 Plasma Etching

It involves removing surface material from the substrate through a combination of physical etching and chemical reactions, producing volatile products. The resulting etched surface exhibits lower-molecular-weight fragments. Typically, inert gases such as argon or helium, as well as nitrogen or oxygen plasmas, are employed for etching polymers.^179^ Caschera et al.^186^ employed plasma etching as a pretreatment to modify cellulosic fiber fabrics prior to diamond-like carbon (DLC) film deposition. Cellulosic fibers substrates were treated with different plasma gases, O_2_, Ar, and H_2_, to optimize DLC nucleation. This approach successfully achieved extremes of wettability, producing surfaces that were both superhydrophilic WCA via plasma etching, and durable superhydrophobic WCA up to 146° by applying a subsequent DLC film to the roughened surface. The research demonstrated that specific plasma pretreatments, notably oxygen plasma, significantly influence the surface morphology and enhance the resulting hydrophobicity and self-cleaning capabilities, with the superhydrophobic effect showing long-term stability for up to 12 months.

However, we could not find a study employing plasma etching in combination with natural compounds on natural textiles.

##### 4.2.2.3 Laser Ablation

It is a process in which a high-intensity laser beam removes material from a solid surface. (Figure 14, Table 13) When the laser beam illuminates the sample, the beam energy is absorbed by the material, causing localized heating, rapid vaporization or ionization, and consequently the removal of material in the form of charged particles and neutral aggregates. The process occurs in several temporally and spatially separate stages: initially, the emission of electrons, followed by the release of ions, atoms, molecules, clusters, and particles.^187,188^ The material absorbs the laser beam’s energy at sufficiently high fluence or with strong pulses, electrons are excited into high-energy states (for ultrashort pulses, also following a two-temperature model). At this stage, chemical bonds are broken, and extensive ionization occurs; electrons and ionized species can induce a Coulomb explosion, especially with ultrashort pulses.^189,190^ As the material becomes highly excited, a plasma cloud forms containing ions, electrons, neutral atoms, and clusters. This cloud expands away from the surface—in vacuum, gas, or liquid—with rapidly propagating shock fronts, contact surfaces, and ionization fronts. During this stage, processes such as hydrodynamic expansion, plasma collapse, plasma-ambient gas interaction, and recombination strongly influence the rate and extent of material removal.^190,191^ Simultaneously or following the plasma phase, mechanical and thermal ejections can occur at the surface—droplets, fine particles, clusters—which are expelled from the surface. These species may be neutral or charged and can condense back on the surface or remain in the plume. Significantly, the velocity, direction, and composition of the ejected material depend on the laser parameters (pulse width, repetition rate, fluence, wavelength), the type of material (metals, polymers, ceramics), and the surrounding atmosphere (vacuum, gas, liquid).^187,192^ As the plasma expands and loses energy, it begins to cool; ionization decreases, electrons and ions recombine, and neutral atoms condense. Clusters grow and aggregate, leading to the formation of particles. At this stage, redeposition of material back onto the surface may occur, particularly at higher ambient pressures, which can affect the resulting surface morphology and material residues. The result is the removal of material from the surface (as vapor, plasma, or ejecta), leaving behind a groove, opening, modified micro- or nanostructure, residual layers, or altered chemical/structural states. Furthermore, laser ablation can induce thermal effects, back-reactions, molecular decomposition, and phase transitions within the material.^187,188^ The core equipment for laser ablation systems consists of three primary components: the laser source, the optical system (for focusing and conditioning the beam), and the target/sample stage within a controlled ablation chamber.

**Figure 14:**
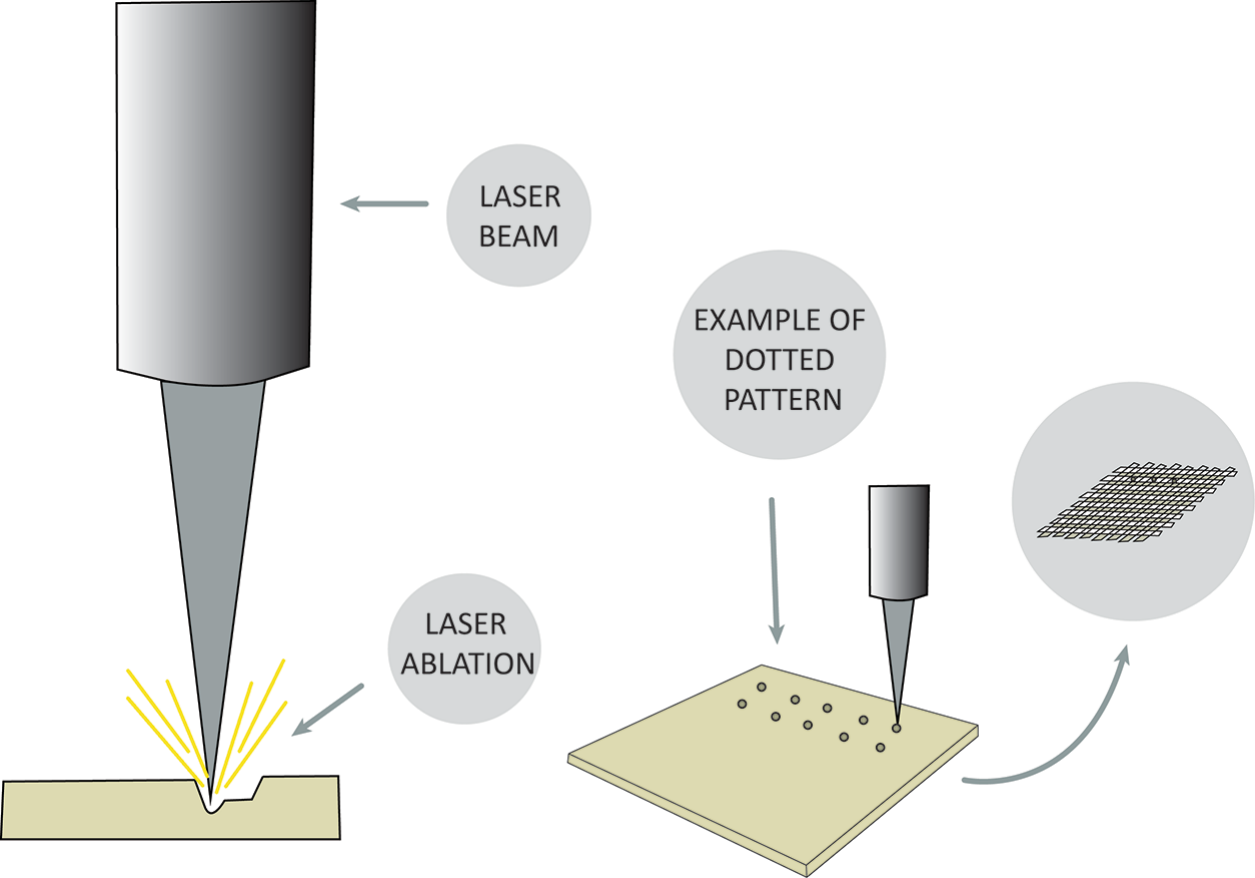
Schematic presentation of laser ablation: when the laser beam illuminates the sample, the beam energy is absorbed by the material, causing localized heating, rapid vaporization or ionization, and consequently the removal of material.

**Table 13:** Nature-Based Hydrophobic Coatings Using Laser Method: Used Methods, Reaction Mechanism, Textile Substrate, Materials, Process Steps, and Treatment Conditions, WCA in SA

Methods used and reaction mechanisms	Textile substrate and coating materials	Process steps and treatment conditions	Hydrophobic performance and durability	ref
PDC, laser dotting (L)	cellulosic fibers	1.) Preparation of coating solution,	WCA (PT) = 145°, WCA (PT, laser power 2,4w) = 138°, WCA (PT, laser power 4W) = 110°	^64^
physical interactions (hydrogen bonding)	persimmon tannin	2.) impregnation and padding for 15 min,	WCA (250 domestic washing cycles) = 97°, WCA (15000 cycles) = 134°	
		3.) dry at 120 °C for 20 min,		
		4.) laser treatment (CO_2_ laser engraving cutting machine, output power 80w, laser power 4w, speed 300 mm/s, dot pattern.		

Laser ablation is conceptually simple but technically complex, requiring experts for safe, precise, and optimal operation.^190^ The process is rapid, requiring no complex chemical dissolution procedures, and can be performed in situ with minimal sample preparation.^188^ It offers high precision and accuracy in material removal, enabling the creation of intricate patterns and structures. It causes minimal thermal damage to the surrounding areas, preserving the material’s integrity and properties. The process often results in minimal contamination because there is no physical contact between the laser and the material. Laser ablation can achieve high processing speeds, making it efficient for various industrial and research applications. Waste generation is minimized, as the process requires only micrograms of material and, being a dry method, eliminates the need for water and produces less waste.^188^ However, the method has its drawbacks. The scalability of laser ablation, defined by high throughput and quality, is fundamentally limited by plasma shielding and heat accumulation at high power densities, which cause ablation efficiency to plateau and surface quality to degrade.^187^

The initial cost of laser systems can be substantial, especially for high-powered lasers and specialized setups. It requires stringent safety measures. Some materials may not respond well to laser ablation due to their optical or thermal properties, limiting the applicability of the process^187,188,192,193^ From an energy perspective, laser ablation is energy-intensive, requiring high-power lasers to deliver short energy bursts.^188^ Laser-only approaches are not sufficient in durability; however, laser patterning layered onto a hydrophobized fibrous matrix can be durable. Tian et al.^64^ employed an 80 W CO_2_ laser engraving and cutting machine to imprint a dotted pattern onto cellulosic fabric treated with persismon tanin, with its primary function to selectively degrade the hydrophobic coating and cellulose fibers, creating specific morphological and chemical changes that enable directional water transport. Throughout the experiment, the laser machine maintained a consistent distance between the laser nozzle and the working platform. The dot patterns were meticulously applied to the fabric at a speed of 300 mm/s. The side exposed to the laser beam was defined as the top surface, while the opposite side was denoted as the back. The areas with and without the dot patterns were labeled as the dotted area and the nondot area, respectively. A water drip test confirmed that droplets could travel to the fabric’s top side through the dotted area under antigravity conditions, but they could not penetrate in the reverse direction. The initial treatment of cotton fabric with persismon tanin successfully converted the naturally hydrophilic cotton into a hydrophobic material with a WCA of ∼145°. At the same time, laser-textured dot areas became more wettable, driving directional water transport. This kind of treatment was durable even after 250 domestic washing and 1500 abrasion cycles, achieving WCA of ∼97° and 134°, respectively. For the sole hydrophobic effect as described above, the laser technique was used only in combination with synthetic compounds and nontextile substrates.^194^

### 4.3 Other Methods and Emerging Techniques

Those methods were not yet used in combination with natural compounds on natural textiles, which is why they represent a significant area of research.

#### 4.3.1 Electrospinning

It is a dry process that utilizes a high-voltage electric field to draw a polymer solution or melt from a spinneret into a fine jet, which solidifies into ultrafine fibers (2 nm–several μm). The process relies on the formation of a Taylor cone at the needle tip, solvent evaporation (or cooling), and collection of the fibers on a grounded substrate. Key parameters, solution viscosity, conductivity, applied voltage, flow rate, and ambient temperature/humidity, determine fiber diameter and morphology. The technique can process both synthetic and natural polymers (e.g., CS, cellulose, gelatin), often yielding mats with high surface area, porosity, and tunable mechanical properties.^58,195,196^ Electrospinning can create superhydrophobic textiles with its dual effect. On one hand, it produces fibrous membranes (nanofiber mats) with ultrafine fiber diameters (nanoscale to microscale) and high porosity. This inherent structure, characterized by a high surface area and nanoscale roughness, effectively mimics the hierarchical micro- and nanostructures found in nature. To ensure superhydrophobicity, the electrospun fibers must be composed of or modified with materials that possess low surface energy.^109^

#### 4.3.2 Digital Systems and UV-Curing

These techniques offer a sustainable pathway to engineer highly functional, hydrophobic textile surfaces by optimizing both the application and fixation processes.^158^ UV curing is fundamentally a rapid, energy-efficient alternative to conventional heat-intensive methods, allowing for surface modification without altering the bulk properties of the fabric and eliminating the need for prolonged heating.^24,123^ This process has been successfully utilized to create robust superhydrophobic cotton fabrics, removing the requirement for prolonged high-temperature curing typically associated with conventional methods.^24,67^ When integrated with advanced digital application methods, such as digital finishing or lithography, UV curing enables precision and efficiency, supporting localized finishing applications that reduce chemical use and waste.^123^ For instance, lithography is a sophisticated digital technique used to generate the intricate micro/nanostructures required for achieving superhydrophobicity, while high-speed digital single-pass machines are being commercialized to incorporate rapid curing solutions, like UV curing, directly into the production line to fix coatings.^24,138^ By minimizing solvent emissions and drastically lowering energy consumption during fixation, the combined approach of digital systems and UV curing accelerates the industry’s transition toward resource-efficient and environmentally conscious textile manufacturing.^123^ Taurino et al.^197^ successfully utilized this at the beginning of 2025. The study employed a sol–gel route in which CS was combined with vinyltrimethoxysilane (VTMS) and, in some formulations, TEOS to create hybrid precursors at 2 and 5 wt % concentrations; the mixtures were stirred at 27 °C, hydrolyzed for 30 min, and then applied to pure cotton and polyester fabrics by dip-coating (30 s immersion) or by a custom microsolenoid inkjet 3D printer, followed by a 15 min UV cure at ≈30 mW cm^–2^. WCA results for cotton showed a dramatic increase from the untreated value of 0° to exceeding 120° when the lower-viscosity formulation was used. Moreover, the water-repellent behavior persisted after multiple washing cycles, where contact angles either remained stable or slightly increased due to the removal of residual solvents, confirming the robustness of the hybrid coating.

#### 4.3.3 Supercritical Carbon Dioxide CO_2_

The technique stands out as a highly innovative and sustainable textile processing method, leveraging CO_2_ above its critical point (around 31 °C and 72 bar) to function as a powerful, nontoxic, and reusable solvent medium that replaces water and hazardous chemical solvents. This dry process eliminates industrial wastewater generation and significantly reduces the energy required for processing and drying, marking a transformative step toward green chemistry in textile manufacturing. Due to its nonpolar nature and high diffusivity, it excels as a carrier for various hydrophobic finishing agents, including those derived from natural resources.^24^ Examples of its successful application include the use of high-pressure CO_2_ impregnation methods to introduce alkyl ketene dimer (AKD) onto cellulose fibers, leading to uniform coverage and WCA of ∼140°.^198^ Additionally, this method has demonstrated utility in solubilizing and depositing other natural nonpolar compounds, such as certain oils and beeswax, for cleaning or finishing applications, thus validating its potential to integrate biobased, renewable compounds into high-performance, water-repellent fabrics.^198^

#### 4.3.4 Foam Finishing

It is environmentally friendly and a highly efficient alternative to conventional wet processing methods for imparting hydrophobic properties to textiles, offering significant advantages in sustainability and product quality. This technique uses air as a partial replacement for water, resulting in much lower liquid uptake by the fabric, often by 30 to 90%. It can reduce wet pick up values of 60–80% to a range of 25–35% for cellulosic materials, leading to quicker drying and energy efficiency.^199^ By depositing materials predominantly on the surface of the textile with limited penetration into the fibers, foam finishing produces lighter and thinner textiles.^24^

## 5 Sustainability Assessment and Discussion

Sustainability was evaluated through an integrated assessment of environmental impact and industrial feasibility, focusing exclusively on naturally derived, PFAS-free hydrophobic coatings. These systems are assumed to be inherently low in toxicity and biodegradable, so these aspects were not assessed separately. It is important to note that the analysis is based on estimations from literature rather than experimental data, and the criteria are limited to gate-to-gate impacts, not a full life-cycle sustainability assessment.

### 5.1 Environmental Impact Assessment

Gate-to-gate environmental impact criteria were determined as outlined in Section 3 and are presented in Table 14 and Figure 15. The resulting semiquantitative overall environmental impact assessment is summarized in Table 15.

**Figure 15:**
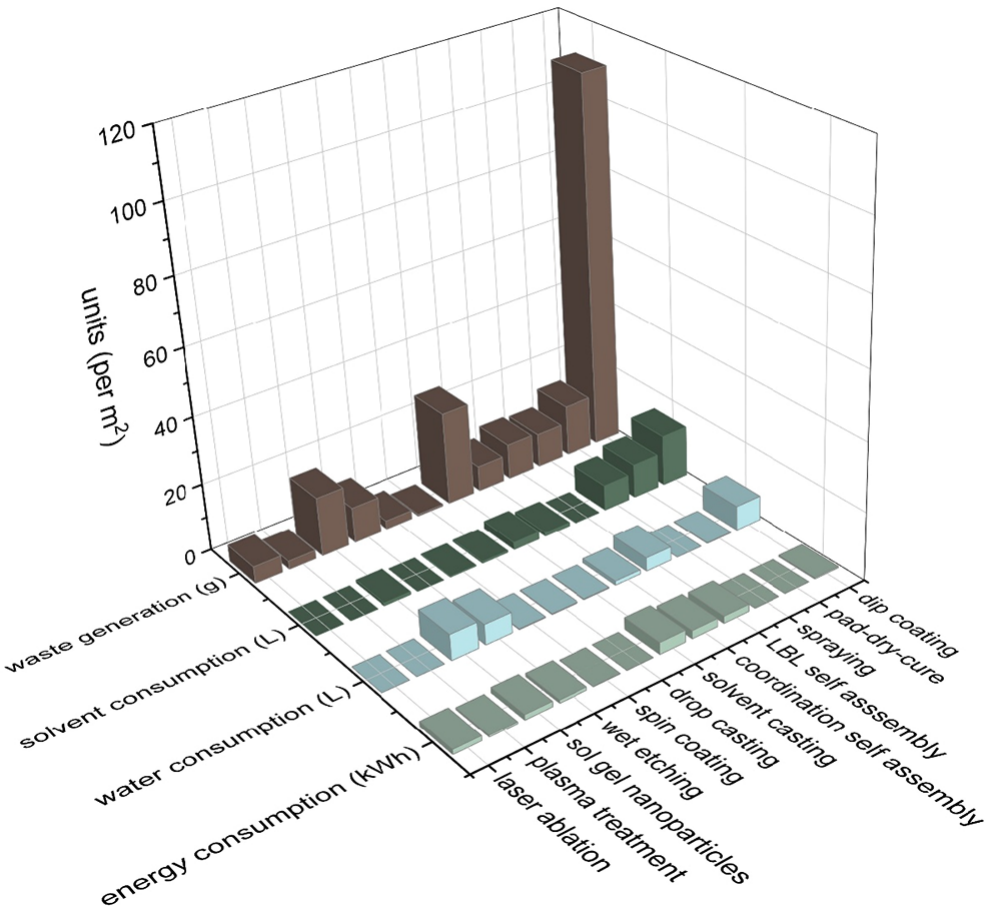
Estimated quantitative values of energy, water, solvent and waste generation for nature-based hydrophobic methods: dip coating, PDC, spraying, LBL self-assembly, coordination self-assembly, solvent casting, drop casting, spin coating, wet etching, sol gel-nanoparticle method plasma treatment, laser ablation.

**Table 14:** Quantitative Analysis of Natural-Based Methods for Hydrophobicity on Natural Textiles, with Calculated Values of Gate to Gate Impacts: Energy Use, Water Consumption, and Waste Generation per 1 m^2^ of Cotton, Grammage 200 g/m^2^

	Energy use (kWh)	Water consumption (L)	Solvent consumption (L)	Waste generation (g)
Dip coating	∼0.26–0.53	∼2.4–12.0	∼0–30	∼30–190
Pad-dry-cure	∼0.04–0.1	∼0.1–0.3	∼0–20	∼10–20
Spraying	∼0.02–0.09	∼0.02–0.06	∼0–15	∼5–15
LBL self-assembly	∼0.45–3.77	∼2–6	∼0	∼6–15
Coordination self-assembly	∼0.59–4.19	∼0.5–2	∼0.50–1.50	∼0–15
Solvent casting	∼1.39–5.56	∼0–0,5	∼0.3–4	∼5–50
Drop casting	∼0.014–0.139	∼0–0,05	∼0.01–0.1	∼0–1
Spin coating	∼0.03–0.28	∼0–0.05	∼0.05–0.5	∼0–5
Wet etching	∼0,28–1,67	∼2–10	0	∼1–20
Sol gel nanoparticles	∼0.42–2.78	∼0.2–1.5	∼0.2–1.5	∼5–30
Plasma treatment (atmospheric)	∼0.25–0.83	0	0	∼0–5
Laser ablation	∼0.14–2.78	0	0	∼0–10

**Table 15:**
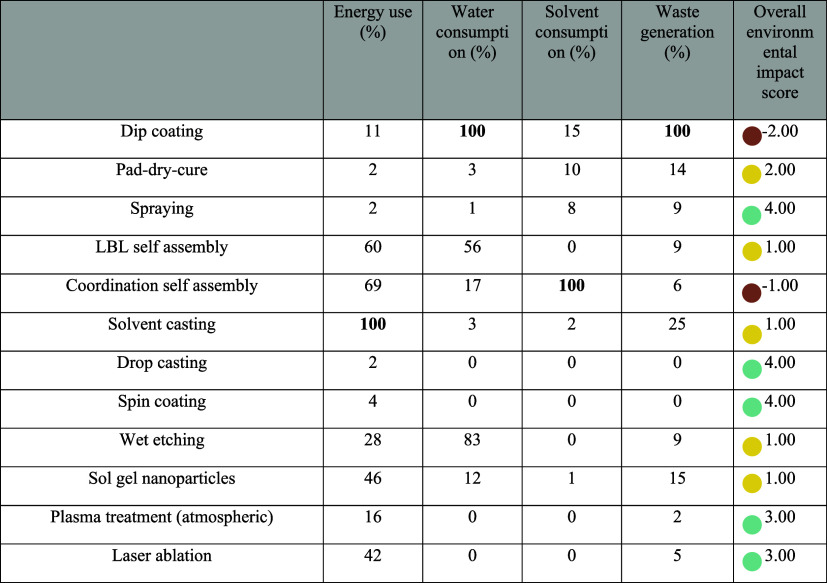
Overall Environmental Impact Classified According to the Final Score: +4 (Very Low Environmental Impact; Highly Favorable), +3 (Low Impact), Marked with Sky Blue Circle Solid, +2 (Moderate Impact), +1 (Slightly Elevated Impact) Marked with Yellow Circle Solid, 0 (Moderate Environmental Impact) Marked with Gray Circle Solid, −1 (Elevated Impact), −2 (High Impact) Marked with Brown Circle Solid, −3 (Very High Impact), and −4 (Extremely High Impact; Environmentally Burdensome) Marked with Dark Brown Circle Solid

#### 5.1.1 Energy Consumption

Energy consumption depends on the process type (wet or dry), thermal requirements and conditions, fluid/pressure management, substrate saturation, and formulation. Wet processing techniques—particularly multilayer approaches requiring repeated evaporation steps, such as self-assembly methods or prolonged solvent/water removal in solvent-casting processes—are often most energy-intensive (Figure 15, Table 14). Large volumes of liquid must be evaporated, necessitating extensive drying and frequently additional curing stages. Energy demand is further amplified by solvent volatility and temperature–time processing profiles.^89,130^ Dry techniques generally require less energy overall, as they avoid water removal; however, they still necessitate electrical power for plasma generation, gas flow, or pressure maintenance, with the exact draw depending on power, duration, and gas composition. Material factors—such as solvent content, the need for post-treatment curing, and the intrinsic thermal stability of the coating—also affect the energy required. Meanwhile, scale-up efficiency, equipment design, and heat integration strategies can either mitigate or accelerate the overall energy footprint.^70^

#### 5.1.2 Solvent and Water Consumption

The consumption of solvents and water strongly depends on the type of hydrophobization technique employed. To align with environmental, health, and safety (EHS) mandates, waterborne methods are preferable for manufacturing coatings compared to organic solvent-based methods, as they significantly reduce the release of VOCs, minimize fire and explosion hazards, and lower overall toxicity risks associated with petroleum-derived liquids.^19^ The chosen method must also obey wastewater and effluent compliance (facility level). Jurisdictions set discharge limits for pH, COD, BOD, TSS, oil/grease, metals, cyanide, etc. (e.g., EU Water Framework; U.S. Clean Water Act/NPDES permits). Textile finishing must ensure compliant pretreatment and advanced treatment (coagulation, flocculation, biological treatment, membrane/adsorptive polishing) to meet permitted limits and protect receiving waters.^15^

Wet-chemical methods typically require large volumes of water or organic solvents for preparing dispersions, rinsing, and cleaning steps. These methods often generate wastewater containing residual chemicals or solvents, thereby contributing to greater environmental impact.^24^ Catarino et al.^24^ reported that use of plasma technology in finishing was associated with a 66% reduction in water consumption and a ∼60% reduction in wastewater loads compared to conventional wet-chemical processes. Dry techniques eliminate the use of water and solvents, which is why they are often considered to have lower environmental impact. As seen from our analysis (Figure 15, Table 14) dip coating consumes the most water and solvents as it relies on large solution reservoirs. The substrate must be fully immersed in the coating bath, requiring a substantial initial volume of solution. Additionally, numerous treatment and rinsing steps, along with solvent evaporation during processing, further contribute to high solvent and water consumption. Next in terms of water consumption is the wet etching technique, which involves repeated washing and neutralization steps to remove reaction byproducts and improve surface quality. In terms of solvent consumption, solvent casting ranks second, as it typically requires high concentrations of organic solvents to dissolve hydrophobic coating materials and form uniform films during the evaporation process.^200^

#### 5.1.3 Waste Generation, End of Life, and Circularity

The amount of waste depends on process design factors (dry/wet), the use of solvent, and the choice of carrier. Aqueous systems minimize VOC emissions but still produce aqueous effluent; green solvent guidance recommends short alcohols, alkanes, and water, with recovery loops when organics are required.^19^ Wet routes can be water-borne, but they create rinse waters loaded with salts, catalysts, acids/bases, and suspended NPS that need treatment.^145^ Another contributor to waste generation lies in infrastructure, policy, and system-level factors. Additionally, the performance and durability of the treated textile, as well as the availability of repair and reuse pathways, significantly influence overall waste output. Waste generation at the end of life also depends on factors such as biodegradability, recyclability, and the feasibility of material recovery.^201^ Coatings based on natural compounds help reduce end-of-life environmental burdens, as they are biodegradable and enable organic recycling through composting, transforming waste into valuable compost rather than relying on incineration or landfilling, which can release potent greenhouse gases such as methane. Moreover, natural coatings are generally associated with lower greenhouse gas emissions, including CO_2_, throughout their entire life cycle—from raw material extraction and production to product use and disposal. In contrast to synthetic, fossil-based coatings, natural compounds typically require less energy during synthesis, contain fewer toxic reagents or solvents, and do not generate persistent microplastics during degradation.^174,202,203^ Their renewable origin, nontoxic degradation products, and compatibility with circular waste management systems make them a more sustainable alternative, contributing to reduced carbon footprint and enhanced material circularity. However, achieving high durability and long-term performance solely with natural-based coatings remains a significant challenge.^59,204^ To improve mechanical stability, water repellence, and wear resistance, hybrid systems that combine natural and synthetic components are often employed. While such hybrid coatings extend product lifespan and thus enhance sustainability from a durability perspective, they simultaneously compromise environmental compatibility at the end of their life. The inclusion of synthetic fractions can hinder biodegradability, complicate recycling processes, and reintroduce concerns associated with microplastic formation or toxic degradation residues. This creates a critical trade-off between durability and degradability: increasing one often diminishes the other.^204^ Therefore, future research must focus on optimizing this balance—developing hybrid or modified natural coatings that achieve long-term functionality without sacrificing their inherent environmental advantages.

Our quantitative analysis (Figure 15, Table 14) showed that among all assessed methods, dip coating generates significantly more waste than others, consistent with previous observations of also high water and solvent consumption. While reusing the coating bath is an essential strategy to reduce this environmental burden, it presents complex challenges, as bath properties fluctuate during continuous operation.^103^ Among wet techniques, spin and drop coating produce the least waste, as they use only small, localized volumes of coating solution. These methods also enable precise thickness control, reducing the need for reprocessing and minimizing waste during optimization. As expected, dry techniques generate minimal waste since they do not involve water or solvents and require no washing or rinsing steps. Material utilization is high because the deposited layer forms directly on the substrate, and unused precursors can often be recovered or recycled, particularly in closed systems. Additionally, dry processes eliminate energy-intensive solvent evaporation and reduce chemical emissions.

#### 5.1.4 Overall Environmental Impact Assessment

As observed in Table 15, spraying, drop casting, and spin coating exhibit the lowest environmental impact according to the proposed scoring system, whereas dip coating and coordination self-assembly rank lowest. Among the assessed techniques, dip coating exhibits the highest water consumption and waste generation, whereas coordination self-assembly scores the lowest due to its extensive use of solvents. Solvent casting shows the greatest energy demand, further reducing its sustainability score. Conversely, dry techniques generally perform favorably across multiple parameters, reflecting their lower environmental impact and operational efficiency. The results emphasize the importance of selecting coating methodologies that minimize solvent use, water consumption, and energy requirements to achieve safer and more sustainable textile finishing processes.

### 5.2 Industrial Feasibility Assessment

The practical feasibility of the methods (Table 16) at the industrial scale was evaluated as described in Section 3. The scoring scheme is defined as follows: 4 (very highly industrially feasible), 3 (highly feasible with minor limitations), 2 (moderately feasible/possible with some optimization), 1 (limited feasibility but possible), 0 (neither feasible nor infeasible), −1 (difficult to implement at industrial scale), −2 (very difficult to implement at industrial scale), −3 extremely challenging (mostly lab scale), −4 (not industrially feasible). Additionally, they were grouped into 3 main classes depending on industrial maturity which refers to the current adoption of the method in actual industrial practice, including availability of infrastructure, operator experience, and integration into existing production lines: established (widely implemented in textile manufacturing), emerging (demonstrated at pilot or early commercial scale), lab-scale (primarily restricted to research environments).

**Table 16:** Hydrophobic Methods and Evaluation of Their Industrial Feasibility Criteria, Their Industrial Relevance, Key Evidence, and Feasibility Scores

**Industrial Feasibility Criteria**	**Industrial relevance**	**Key evidence**	**Score**
**DIP COATING**			
Scalability and equipment	Compatible with standard textile finishing lines.^33,70,100,104^	Continuous dip lines; roll-to-roll yarn and nonwoven handling established^33,70,100,104^	**4**
Time-efficiency	Moderate multistage wet cycle	Immersion + withdrawal + drying/curing ≈ 10–35 min^114^	**2**
Process control	Mature but substrate-dependent variability	Thickness governed by viscosity, surface tension, withdrawal speed^104,138,199^	**2**
Performance	Superhydrophobicity achievable	WCA > 150° with nano/micro roughness + low-energy binders^114^	**4**
Durability	Moderate; binder dependent.^100,108,129^	10–30 wash cycles or ∼30 abrasion cycles are typical^38,69^	**1**
Cost	Low CAPEX but water/effluent intensive^19,70,199^	High water use; often repeated multiple cycles to reach the desired add-on or build multilayers, compatible with biobased chemistries^19,70,199^	**1**
Final Score			**2**
**PAD-DRY-CURE**			
Scalability, time, and equipment	Dominant industrial wet finishing route.^205^	Uses padding mangles, dryers, stenters without hardware change^205^	**4**
Time efficiency	Short continuous cycle^124^	Total process ≈ 5–15 min^124^	**3**
Process control	Mature control; migration risk manageable^123,206^	Wet pickup, nip pressure, and curing profile are critical	**3**
Performance	Superhydrophobicity achievable	WCA > 150° with nanoparticle-enabled systems^88^	**4**
Durability	Moderate-high with cross-linkers^2^	10–30 wash cycles; abrasion resistance moderate	**2**
Cost	High throughput but water- and energy-intensive.^70^	High drying load; mandatory effluent treatment^24^	**2**
Final Score			**3**
**SPRAYING**			
Scalability and equipment	Continuous roll-to-roll compatible.^138,148^	Nozzle bars on open-width lines; major liquor reduction^138,148^	**4**
Time efficiency	Fast deposition; moderate drying.	Spray seconds; drying ≈ 10–30 min^138,148^	**2**
Process control	Good with automated systems.^138,206^	Droplet size, pressure, and traverse pattern determine uniformity.^24,101^	**3**
Performance	Superhydrophobicity achievable.	WCA ≥ 150° across multiple material systems.^59,69,131^	**4**
Durability	Chemistry dependent; moderate overall.^98^	Tens–hundreds abrasion cycles; wash durability variable (reported up to 50 wash cycles).^59^	**2**
Cost	Efficient chemical use; higher equipment cost.^24,138^	Low effluent: enclosed systems increase CAPEX.^24,138^	**2**
Final Score			**3**
**LBL SELF-ASSEMBLY**			
Scalability and equipment	Multibath sequential process.^143^	Roll-to-roll implementation challenging.^58,143^	**-3**
Time efficiency	Repetitive immersion cycles.^147^	≥50–70 min minimum processing time (one bilayer).^147^	**-1**
Process control	Excellent nanoscale tunability.^36,59^	Precise layer thickness and chemistry control.^36,59^	**4**
Performance	High hydrophobicity achievable.	WCA ≈ 130–160°^147^	**3**
Durability	Poor durability.^70,145^	Weak wash fastness (WCA drops after 1 wash cycle). layer delamination common.^35^	**-2**
Cost	Low CAPEX; low throughput.^58,143^	Multiple passes, long process times, multiple baths increase OPEX, simple equipment.^207–209^	**-1**
Final Score			**0**
**COORDINATION SELF-ASSEMBLY**			
Scalability and equipment	Uses standard dip/pad lines.^113,151^	Multiple wet stages increase line length.^113,151^	**2**
Time efficiency	Very slow ligand adsorption.^149^	Total process often ≈8–16 h but faster complexation routes reported.^149^	**-3**
Process control	Highly tunable coordination chemistry.^142,210^	Stoichiometry, pH, solvent critical.^142,210^	**3**
Performance	High hydrophobicity achievable.^151^	WCA ≈ 140–150°^151^	**4**
Durability	Strong metal–ligand anchoring.^152^	Stable after 25 wash cycles, UV (after 24 h), 7-day solvents immersion, repeated abrasion, peeling.	**3**
Cost	Low material cost; moderate throughput.^151,152^	Aqueous processing lowers energy demand.^151,152^	**2**
Final Score			**2**
**SOLVENT CASTING**			
Scalability and equipment	Adaptable from film coating lines.^101,130^	Best for flat webs; drying limits width/thickness.^136,195^	**2**
Time efficiency	Extremely slow solvent evaporation.^136,195^	Total process ≈ 14–24 h^136,195^	**-4**
Process control	Sensitive to solvent and evaporation kinetics.^168,195^	Homogeneous films achievable with optimization^168,195^	**2**
Performance	Moderate hydrophobicity.^63^	WCA ≈ 130°^63^	**1**
Durability	Strong cohesive films.^130,168^	Crack risk under flexing.^195^	**3**
Cost	High solvent recovery and energy demand.	VOC control increases CAPEX/OPEX.^130^	**-2**
Final Score			**0**
**DROP CASTING**			
Scalability and equipment	Serial small-area process.^211^	Impractical for large textile areas.^128^	**-4**
Time efficiency	Fast deposition; slow drying.^128,212^	Deposition + drying ≈ 1–4 h.^128,212^	**-2**
Process control	Poor uniformity due to coffee-ring effect.^211^	Reproducibility challenges.^212^	**-2**
Performance	Limited hydrophobicity evidence.^65^	WCA > 90°^65^	**0**
Durability	Thickness gradients; stress cracking.^99,212^	Delamination risk high.^99,212^	**-2**
Cost	Very low CAPEX/OPEX.	Low throughput offsets savings.^128^	**3**
Final Score			**-1**
**SPIN COATING**			
Scalability and equipment	Batch single-substrate process.	Unsuitable for roll-to-roll textiles.^101^	**-4**
Time efficiency	Very rapid thin-film deposition.	Total cycle ≈ 1–3 min.^138^	**4**
Process control	Excellent thickness uniformity.^101^	Spin speed–viscosity relationship well-defined.	**4**
Performance	Moderate hydrophobicity.	WCA ≈ 90°^141^	**1**
Durability	Adhesion-dependent on textiles.^101,104^	Crack/delamination risk.^101,104^	**1**
Cost	Moderate equipment; poor material utilization.^101^	Low energy per run but huge solution waste and single-substrate, batch operation.^101^	**-1**
Final Score			**1**
**WET ETCHING**			
Scalability and equipment	Compatible with wet processing machines.^4,37^	Continuous bath processing feasible.^4,37^	**3**
Time efficiency	Long wet processing cycle.	Etch + rinsing + curing ≈ 2.5–3 h^4^	**-2**
Process control	Chemistry-sensitive.	Tight bath control is required to prevent fiber damage.^37^	**2**
Performance	Enables durable roughness.^4,38^	With hydrophobe: WCA ≈ 155–160° ^4,38^	**4**
Durability	Permanent topography.^4,38^	30–50 wash cycles; 400–1200 abrasion cycles.^4,38^	**3**
Cost	Low CAPEX; chemical and energy driven OPEX.^4,38^	Cheap tanks, but effluent treatment required.^4,38^	**1**
Final Score			**2**
**SOL GEL NANOPARTICLES**			
Scalability and equipment	Fully compatible with textile wet lines.^104,145,205^	Pad–dry–cure, spray, dip integration.^205^	**3**
Time efficiency	Moderate thermal cycle.	Application + drying + curing ≈ 20–60 min on continuous lines.^66^	**2**
Process control	High chemical tunability.^66^	Controlled hydrolysis/condensation; uniform nanolayers.	**4**
Performance	Superhydrophobicity achievable.	WCA > 150° with minimal handle loss.^66^	**4**
Durability	Strong covalent/hybrid networks.^205^	10–30 + washes; abrasion resistant.^145,197,205^	**3**
Cost	Moderate CAPEX/OPEX.	Thin add-on offsets precursor cost,^145,205^ uses pad/spray hardware.	**1**
Final Score			**3**
**PLASMA (ATMOSPHERIC) TREATMENT**			
Scalability and equipment	Roll-to-roll modules available.^34^	Inline integration without vacuum systems; industrial textile lines demonstrated.^34^	**2**
Time efficiency	Extremely fast inline process	Industrial speeds up to tens of m/min.^150^ Even at these slower optimization speeds lab scale (e.g., 5 mm/s), ≈3–4 min.^213^	**4**
Process control	System-specific recipe tuning required.^34,214^	Power, gas mix, gap distance critical.^34,109^	**2**
Performance	Superhydrophobicity achievable.	WCA ≈ 150–160° ^109,213^	**4**
Durability	Moderate; improved as pretreatment.^34,109,185^	Abrasion resistance remains barrier.^109^	**1**
Cost	High CAPEX; low water footprint.^109,215,216^	Electricity & gas dominate OPEX.^109,215,216^	**1**
Final Score			**2**
**LASER ABLATION**			
Scalability and equipment	Industrial garment lasers available.^187,192^	Resolution limits wide-web throughput.	**2**
Time efficiency	Laser texturing: few minutes per sample.	Minutes per area/panel.	**1**
Process control	High precision parameter control.^67,128^	Fluence, pulse count, scan speed adjustable- ^187,192,217^	**2**
Performance	Superhydrophobicity via hybrid routes.^187,192,217^	WCA ≥ 150° with hydrophobe^187,192,217^	**4**
Durability	Texture intrinsic to substrate.^194^	No delamination; high robustness.^194^	**4**
Cost	Energy & equipment intensive.^21,192^	High CAPEX; specialized operation.^21,192^	**-3**
Final Score			**2**

#### 5.2.1 Simplicity and Scalability

A method is considered simple if it is easy to formulate, easy to run on existing lines, needs little specialized equipment, and scales without much retraining or retooling. Judged by those criteria, most wet hydrophobization routes are simpler than dry routes, even though dry routes can be more controllable.^58^ Wet techniques are considered simpler in formulation and integration with existing infrastructure, whereas dry techniques entail greater technological complexity due to specialized equipment and intricate process tuning.^34^

For a process to be scalable, it must meet the following industry requirements. The process must be continuous, roll-to-roll processing at textile widths and speeds. Equipment must handle widths of 1.5–10 m with uniform treatment across the web and a throughput of more than 10 m/min. Uniformity across the lateral dimension and elimination of edge effects are critical for quality at scale.^34^ Processes should operate “cool” (near room temperature or <100 °C) to be compatible with most substrates and not degrade fibers.^34^ The industry prefers atmospheric, inline systems over batch vacuum systems. The process must have proven uniformity and process control at scale.^24^ Systems should be able to integrate with the existing finishing line and workforce.^34^ Processes using solvents or CO_2_ must be closed-loop with efficient recovery to minimize energy and emissions.^206^ It is also essential to evaluate the systems and the viable supply chain.^21^ Multiple step coating strategies, prolonged drying phases, or tightly controlled reaction conditions can reduce throughput and increase operational costs at scale. In contrast, processes compatible with existing industrial infrastructure—such as padding and spraying—offer greater scalability due to easier integration and process continuity which is also seen in Figure 16. Equipment adaptability and tolerance to variable textile properties (weave, porosity, fiber composition) further influence scale-up feasibility. Atmospheric-pressure plasma (APP) systems—DBD units, plasma jets, and hybrid corona/DBD heads—are now available as roll-to-roll modules treating widths up to ∼1.5–4 m at several–tens of m/min, and can be integrated before/after padding or coating lines.^34,218^ On the other hand LBL self-assembly technique, solvent casting and drop costing are not appropriate for a scale up. LBL self-assembly requires sequential adsorption/rinse/dry cycles. Each cycle is diffusion-controlled and sensitive to pH, ionic strength, and drying history, so maintaining uniformity over wide fabrics at industrial speeds is difficult.^147^ Even with accelerated variants, LbL is still far more time- and labor-intensive than continuous pad or spray lines.^149^ Solvent casting and drop casting were assessed as the least scalable techniques, as they are restricted to small substrate areas and primarily suitable for simple, flat sheet formats.^102^

**Figure 16:**
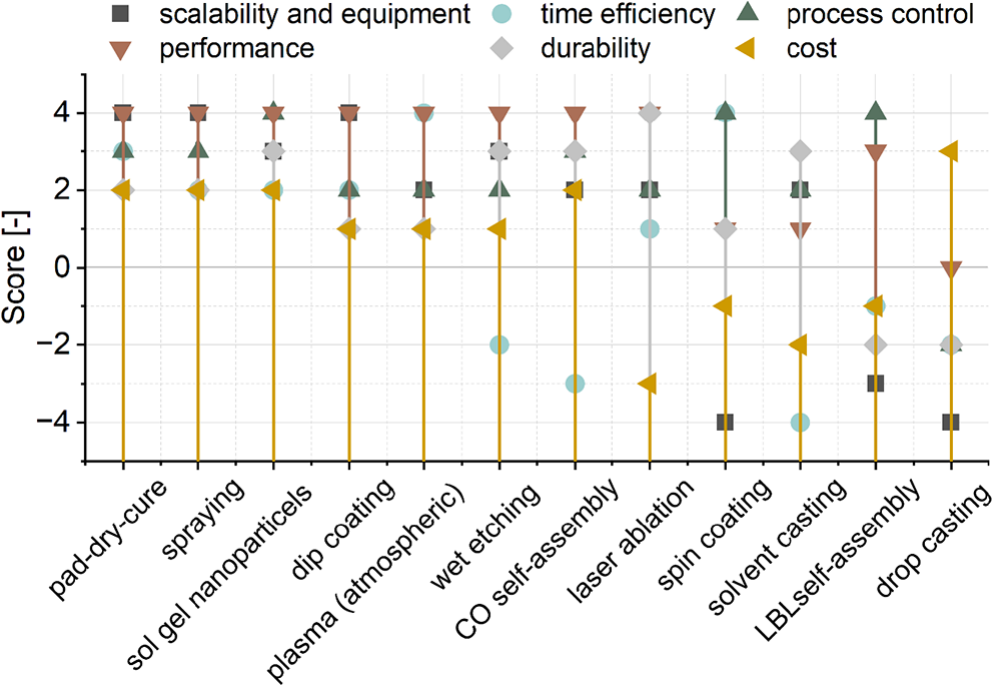
Hydrophobic methods and their assessment according to industrial feasibility criteria: scalability and equipment, time efficiency, process control, performance, durability and cost.

#### 5.2.2 Process Control and Versatility

Small deviations in process control can directly affect contact angle, durability, and cost. Key controlled variables include bath composition (polymer or nanoparticle concentration, solvent ratio, pH, viscosity), substrate speed, pick-up or deposition rate, and drying/curing temperature–time profiles.^130^ For pad-dry-cure and dip coating, solvent choice, diffusion, and evaporation must be modeled to minimize defects and residual solvent, often using binary/ternary diffusion models and free-volume/Flory–Huggins thermodynamics to guide set-points.^130^ Drying conditions (temperature, air flow, residence time) are optimized to avoid sagging, blistering, and gradients in hydrophobicity.^130^ Modern feasibility studies increasingly use Design of Experiments (DoE) or data-driven approaches to quantify how process parameters influence water contact angle, roll-off angle, mechanical properties, and permeability, then locate optimal windows.^219^ For newer methods, uniformity over wide webs is assured by controlling feed rate, temperature, pressure, and line speed, with in-line WCA or XPS sampling to verify coating chemistry and coverage.^220^ Closed-loop control of exhaust humidity or bath properties is also used to maintain constant solids add-on and hydrophobic performance at scale.^136^ Our assessment (Figure 16) shows highest process control for spin coating and LBL-self-assembly technique. Spin coating relies on predictable fluid mechanics. When a substrate rotates, centrifugal forces dominate the flow of the liquid precursor. By adjusting the spin speed and solution viscosity, film thickness at the nanoscale with extreme reproducibility can be controlled. This removes the “guesswork” found in dip or spray coating, where meniscus stability and gravity-induced drainage are highly variable.^104,138^ LbL self-assembly is controlled through building the material one molecular layer (or bilayer) at a time. Because it relies on electrostatic attraction (or hydrogen bonding), the deposition is self-limiting−) once the surface charge is reversed, further adsorption is inhibited. This creates uniform, defect-free layers regardless of the substrate.

Versatility refers to the extent to which a hydrophobization technique can be applied across different materials, processing conditions, and performance objectives. It encompasses several key dimensions, including substrate compatibility, functional diversity, process-mode flexibility, patternability and design control, chemical and technological adaptability, and scalability.^67^ A versatile technique should perform effectively on various textile substrates (e.g., natural, synthetic, or blended fibers) without requiring extensive pretreatment or chemical modification. It should also allow integration of multiple functionalities—such as hydrophobicity, antimicrobial activity, or UV protection—by adjusting formulation components or processing parameters. Process-mode flexibility includes the ability to operate in batch or roll-to-roll configurations, as well as to treat fabrics on one side, both sides, or selectively. Patternability and design control refer to the ability to spatially modulate surface properties, enabling the creation of customized surface patterns or functional gradients. Chemical and technological adaptability describes the method’s compatibility with diverse reagents, solvents, and reaction conditions, allowing it to be combined with other modification strategies or scaled to different materials.^109^ Finally, scalability without fundamental redesign means that the process can be transferred from laboratory to industrial production by adjusting parameters such as coating speed, concentration, or energy input—without altering the underlying mechanism. Most versatile are dry plasma techniques as they can be applied continuously or in a roll-to-roll configuration, to one or both sides of the fabric, or even selectively to specific areas, enabling precise surface modification. They offer high chemical adaptability, as different gaseous precursors can be used to tailor surface functionality without the limitations of solvent compatibility. Plasma also allows patterning and functional gradients, which are difficult to achieve with traditional wet methods. Additionally, these techniques are compatible with a wide range of substrates.^181^ From wet techniques PDC and spray coating are the most versatile.^109^ Their versatility stems from several factors. Both methods enable the controlled deposition of coating solutions, allowing treatment of one or both sides of the fabric, or even localized application with spray or foam coatings. They are compatible with a wide range of waterborne and biobased chemistry. PDC is particularly well-suited for continuous, roll-to-roll industrial lines, allowing for easy scale-up, while spray coating offers flexibility for lab-scale experimentation or patterning.^101^

#### 5.2.3 Time Efficiency

Time depends on process chemistry and kinetics, pretreatments, a multistep sequence, the energy source, reaction acceleration, the deposition technique, and mass transfer control. Microwave-assisted reactions can compress chemical steps to minutes (e.g., 3–15 min microwave times for fatty acid anhydride grafting), but longer exposures may compromise fabric strength, impacting sustainability.^39^ UV-triggered “click” chemistries and photoprocesses generally shorten reaction times compared to traditional techniques, which can take hours to days; this reduction is emphasized to improve production efficiency.^108^ Dry finishing techniques are generally less time-consuming than wet techniques because they avoid long liquor dwell times, rinsing, and extended drying/curing stages.^181^ Among wet techniques, the most time-efficient methods are those that use minimal immersion or localized application rather than full-bath processes and require fewer post-treatment steps, such as PDC and spraying. According to our evaluation (Figure 16), solvent casting was identified as the least time-efficient technique due to prolonged solvent evaporation. Self-assembly methods were also found to be time-inefficient because of their multiple sequential processing steps. Wet etching similarly exhibits low time efficiency, owing to diffusion-limited reaction kinetics, extended immersion and rinsing stages, and mandatory drying steps inherent to liquid-phase processing.^70^

#### 5.2.4 Performance and Durability

Performance encompasses the efficacy of the intended function and how well the treatment maintains the original comfort and physical integrity of the fabric.^129^ Features like self-cleaning enhance sustainability by providing practical benefits such as reducing the need for frequent washing. This translates directly to reduced consumption of water, energy, and chemical detergents by the consumer over the product’s life.^24^ Most from the observed hydrophobic methods, when combined with appropriate formulations, have already achieved superhydrophobic contact angles. The lowest contact angles reported so far were obtained using drop casting and solvent casting techniques, which may also be related to the smaller number of studies employing these methods.

Durability refers to the ability of a textile and its functional properties to withstand real-life conditions over time^114^ It is typically assessed by monitoring the retention of the critical property (e.g., WCA for hydrophobicity) after standardized tests, including laundering cycles (e.g., AATCC 61, ISO 105-C06), sandpaper abrasion cycles (e.g., ASTM D4966),^114^ chemical immersions,^38^ and UV exposure.^40^ Durability directly contributes to environmental sustainability by extending the product’s lifespan, thereby reducing the need for frequent replacement, which in turn lowers the overall burden of manufacturing, resource consumption, and waste generation.^34,114^

The performance and durability of coatings depend on both the application method and the formulation. Although natural-based coatings readily provide high initial performance, ensuring their long-term durability remains more difficult. First, the literature often does not evaluate coating durability comprehensively, or performs only a single test. The use of different standards further complicates direct comparisons. Nature-based coatings have been reported to withstand a maximum of 50 wash cycles and approximately 1,000 abrasion cycles, whereas commercial PFAS-based coatings can demonstrate wash durability of 50–100 cycles^221^ and up to 250,000 abrasion cycles.^222^

Nature based studies from Xu et al.^38^ and Cheng et al.^37^ both employing etching achieved durable superhydrophobic solution, withstanding more than 30 washing, cycles and more than 1000 abrasion cycles. Lower performance and durability were observed in studies using LbL assembly and nanoparticle deposition without cross-linkers, fatty acids applied alone. Fatty acids, although common in biobased hydrophobization, are typically insufficient to achieve high contact angles on their own and must be combined with a surface-roughening step, such as nanoparticle deposition or etching.^4^ To achieve adequate performance and durability (especially robustness), many natural-based coatings still require the inclusion of synthetic compounds (e.g., synthetic binders, cross-linking agents, or NPS like TiO_2_, ZnO), which compromise the coating’s final biodegradability, environmental profile, and cytotoxicity.^81,223^ Study from Huang et al.^63^ compared performance and durability of dip coating and solvent casting technique, depositing the same betulin based coating formulation. The results showed that while dip-coated fabrics achieved a much higher initial WCA solvent-cast or film-coated fabrics maintained significantly better durability which is consistent also with our results in Figure 16. From all the evaluated methods drop casting and LBL self -assembly show the lowest durability score. LBL self -assembly connects the coating on the substrate by electrostatic and hydrogen-bonding interactions between very thin polyelectrolyte layers (nm scale), not by a continuous polymer binder or covalent grafting to the fiber.^144^ Drop casting relies on simple solvent evaporation from sessile droplets, often producing nonuniform films with coffee-ring effects—thicker at the edges or center and thinner elsewhere. These variations arise from uncontrolled flows during drying, resulting in many areas with only a weak, physically adsorbed layer that is easily removed by washing or abrasion.^138,212^ However, due to variations in formulations, it is difficult to determine durability scores with certainty.

#### 5.2.5 Costs

The cost structure of hydrophobization techniques includes both capital (CAPEX) and operating expenses (OPEX). Capital costs cover equipment purchase, installation, integration, facility modifications, and tooling.^24^ Operating costs include utilities (electricity for line drives, dryers, plasma, pumps, heating), consumables (water, solvents, waxes, silanes, sol–gel precursors, binders, surfactants, process gases), labor, maintenance and spare parts, and wastewater or emission treatment to meet environmental regulations. Additional expenses relate to product performance, such as recoating, rework, durability testing, and staff training.^34^ In general, dry techniques tend to have higher initial capital expenditures, while wet techniques generally incur higher operational costs related to resource consumption and waste disposal.^34^ Dry techniques require specialized, high-capital equipment, such as vacuum systems, complex gas handling infrastructure, high-power plasma generators, or laser ablation tools. While the cost per square meter can be competitive or acceptable when the equipment is highly utilized, the initial investment is significant.^34^ Wet chemical application methods rely on established textile machinery (like padding mangles and dryers) that are already standard in most textile plants. This means lower investment or little to no retooling is needed for integration. However, these methods consume significant amounts of water, energy (primarily for drying/curing the water-laden fabric), and chemicals, leading to substantial wastewater volumes that require costly treatment before discharge. Wet chemical treatments often result in higher waste disposal/recycling needs than dry processes.^24,70^ Based on our estimations (Figure 16), solvent casting, spin coating, layer-by-layer (LbL) self-assembly, and laser ablation are among the most costly hydrophobic surface fabrication techniques. In comparison to pad–dry–cure, dip coating, spray coating, and conventional sol–gel processes—which are continuous, high-throughput methods using relatively simple equipment—LbL, solvent casting, spin coating, and laser ablation offer superior process control and high performance on small areas. However, these advantages come at the expense of expensive equipment, high energy consumption (particularly for laser ablation), slow batch processing, and, for solvent-based techniques, significant solvent and energy use. For large-scale PFAS-free hydrophobization of natural textiles, the most cost-effective near-term strategies include PDC or spray applications as seen from Figure 16. Dip coating remains a viable option only where existing lines are already installed, although it entails higher solvent and water consumption, as well as increased waste management costs. Plasma treatments can provide added value as a preactivation step, particularly when the high capital investment can be justified.^109,138,224^

#### 5.2.6 Overall Industrial Feasibility Score

The comparative industrial feasibility assessment highlights substantial differences in the readiness of hydrophobic finishing technologies for large-scale textile manufacturing. While limitations vary across methods, our assessment identifies scalability, time efficiency, and cost as the main challenges, as well as reduced durability relative to traditional PFAS-based coatings. Methods classified as established (Figure 17) demonstrate consistently high feasibility scores, reflecting proven scalability, process reliability, and seamless integration into existing production infrastructure. Among these, spraying and pad–dry–cure show strong feasibility due to their compatibility with continuous roll-to-roll processing, high throughput, and widespread industrial adoption. Sol–gel nanoparticle treatments also fall within the established category, indicating growing industrial implementation despite moderate process complexity. Dip coating, while slightly less feasible due to higher costs and lower durability scores. remains a practical and well-understood finishing route with existing infrastructure. Techniques categorized as emerging (Figure 17) exhibit moderate feasibility scores, suggesting promising industrial potential while still facing barriers to widespread deployment. Atmospheric plasma treatment demonstrates good performance, lower OPEX, and time efficiency; however, limitations related to capital investment, equipment availability, and process uniformity constrain rapid adoption. Similarly, wet etching, coordination self-assembly, and laser ablation show technical viability but require further optimization to improve throughput, cost-efficiency, and integration into continuous textile processing lines. Methods classified as laboratory-scale (Figure 17) present the lowest feasibility scores, reflecting fundamental scale-up constraints. Spin coating, solvent casting, layer-by-layer assembly, and drop casting are primarily batch-based, substrate-size-limited, and dependent on research-grade equipment, making them unsuitable for bulk textile manufacturing. While these techniques provide valuable mechanistic insights and enable precise surface engineering, their industrial translation remains limited under current technological and economic conditions. The results indicate that industrially established wet-finishing platforms remain the most feasible routes for near-term implementation of PFAS-free hydrophobic treatments.

**Figure 17:**
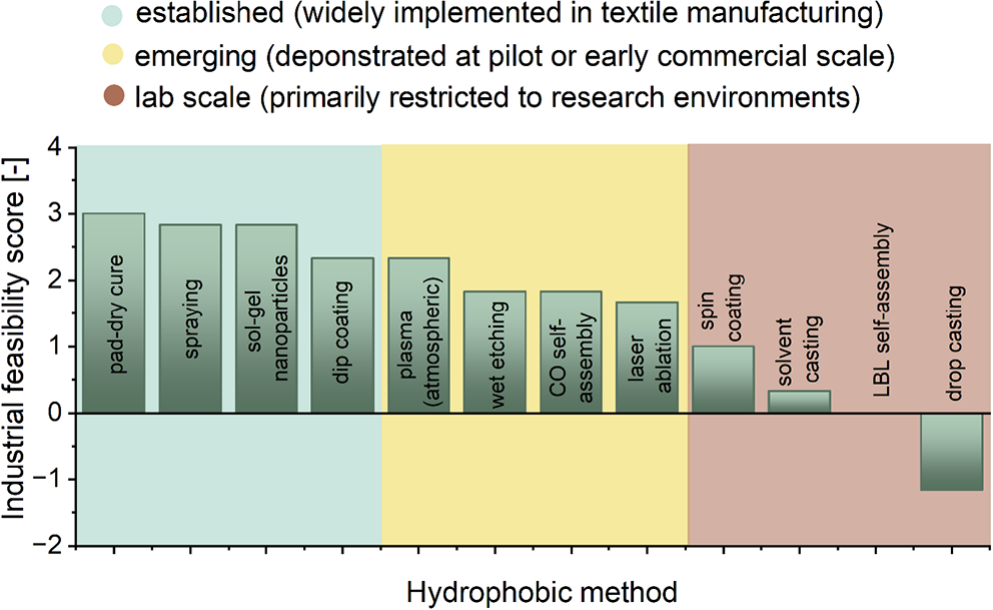
Industrial feasibility of hydrophobic finishing methods based on feasibility scores, categorized by industrial maturity as established, emerging, and laboratory-scale approaches.

### 5.3 Overall Sustainability Assessment and Trade-offs

The comparative sustainability assessment reveals pronounced differences in the trade-off between environmental performance and industrial applicability for the various hydrophobic-finishing techniques (Table 17, Figure 18). As outlined in Section 3, this evaluation is confined to natural-based, PFAS-free coatings, highlighting where certain methods excel environmentally while others offer greater industrial feasibility.

**Figure 18:**
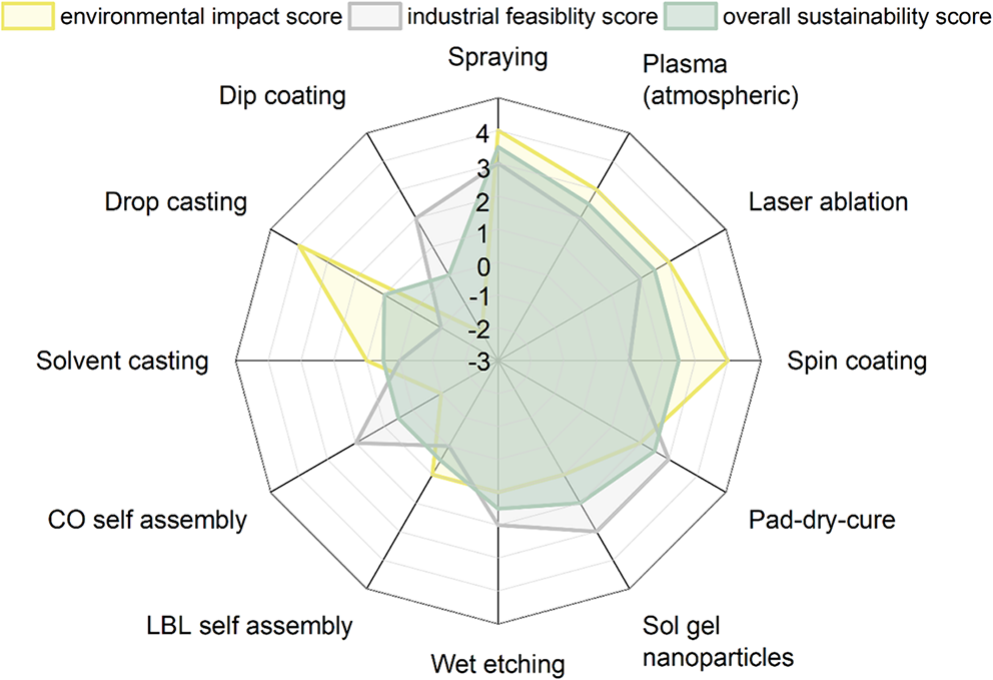
Visual comparison of environmental impact, industrial feasibility, and overall sustainability scores for the assessed methods for hydrophobic modification.

**Table 17:**
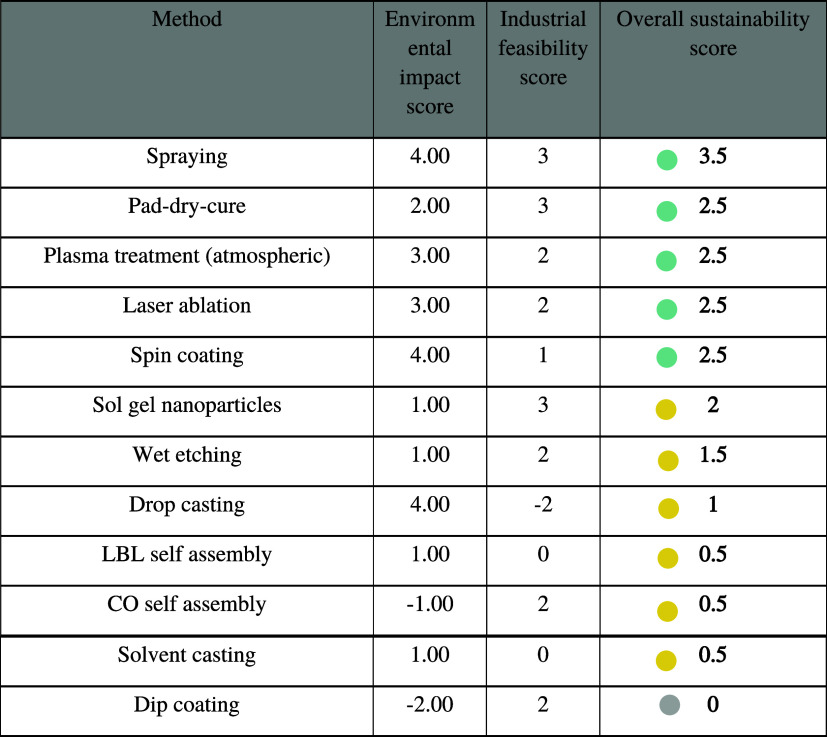
Overall Sustainability Scores Calculated from Environmental Impact Score and Industrial Feasibility Score for Methods Used for Hydrophobization: Spraying, Pad-dry-cure, Laser Ablation, Spin Coating, Sol Gel Nanoparticles, Wet Etching, Drop Casting, LBL Self Assembly, CO Self Assembly, Solvent Casting

Spray coating achieved the highest overall sustainability score (3.5), reflecting a favorable combination of minimal environmental burden and robust industrial feasibility. Compatibility with roll-to-roll processing, reduced chemical waste, and scalable implementation position spray coating as a promising approach for PFAS-free textile finishing. A second tier of methods, including pad–dry–cure, atmospheric plasma treatment, laser ablation, and spin coating, achieved comparable overall scores (2.5) for distinct reasons. Pad–dry–cure continues to serve as a primary industrial platform due to established infrastructure and process reliability, despite moderate environmental impacts related to water and thermal energy consumption. In contrast, plasma and laser technologies offer strong environmental profiles and functional performance but are limited by significant capital investment requirements and integration challenges. Spin coating demonstrates excellent environmental performance but lacks scalability, which restricts its practical application in bulk textile manufacturing. Sol–gel nanoparticle coatings exhibit strong industrial feasibility but are disadvantaged by environmental concerns associated with precursor chemistry and solvent usage, resulting in a moderate overall sustainability score (2.0). Lower-tier methods, including wet etching, drop casting, layer-by-layer assembly, coordination self-assembly, solvent casting, and dip coating, demonstrate limited sustainability due to trade-offs among scalability, process efficiency, and environmental impact. Laboratory-scale techniques such as drop casting and layer-by-layer assembly achieve favorable environmental scores but lack industrial feasibility, restricting their use to research contexts. In contrast, dip coating and coordination self-assembly offer moderate feasibility but incur higher environmental burdens, which diminishes their overall sustainability ranking.

The findings suggest that the most feasible near-term transition toward sustainable hydrophobic textile finishing involves optimizing scalable coating platforms, particularly spraying and pad–dry–cure. Concurrently, the maturation of advanced surface engineering technologies and insights from laboratory-scale methods will inform future mechanistic understanding.

## 6 Evaluation of the Frequency of Applied Techniques

The frequency of methods employed among research studies (lab scale) using at least one natural compound for hydrophobization of natural textiles during the periods 2011–2024 and 2022–2024 is shown in Figure 19. It can be observed that wet processes dominate research and applications in textile surface treatment, accounting for approximately 92% of all processes. Among these, nanoparticle techniques (∼27%), dip coating (∼24%), pad–dry–cure (∼16%), and spray coating (∼11%) are the most commonly used. The widespread preference for wet techniques for hydrophobization, despite their significant environmental drawbacks, stems primarily from a combination of economic, technical, and historical factors related to industrial feasibility, process integration, and familiarity. They are generally simpler, more versatile, and compatible with existing infrastructure, lowering the barrier to adoption compared to high-investment dry technologies.^24^ The inherent hydrophilic nature of most natural compounds, particularly biopolymers, makes them significantly easier and more effective to be used in wet coating techniques (specifically using water as a solvent).^225^ The highest percentage of nanoparticle use marks a significant shift toward nanotechnology-based solutions as they provide the hierarchical surface roughness, durability, and multifunctionality that purely natural or molecular coatings often lack. Nanotechnology offers potential for more sustainable textile finishing, but challenges related to the release of nanoparticles and their impact on human and ecological health must be addressed.^226^ The high prevalence of dip coating in the reviewed studies, despite its previously discussed challenges, can likely be attributed to its simplicity, low cost, and rapid setup in laboratory environments, as well as its broad substrate compatibility. It is also highly compatible with waterborne systems and biobased chemistries, making it particularly suitable for PFAS-free and sustainable treatments.^104^ Moreover, the method is directly translatable to industrial pad–dry–cure lines, facilitating scale-up. The increasing trend in spray coating use (from ∼11 to 16%) can be attributed to modern spray/automatization systems that apply formulations at very low pick-up, reducing drying and, consequently, costs, as well as facilitating easy lab-to-pilot adoption and compatibility with waterborne sol–gel precursors and colloids.^24,101^ This trend is particularly encouraging, as our sustainability assessment identifies spray coating as the most sustainable technique. There has also been an increase in the use of wet etching (from ∼4 to 8%), despite it being a water-intensive technique. This rise is likely driven by the emergence of more sustainable approaches, such as enzymatic treatments and etching with natural phenolic compounds, which offer environmentally friendly alternatives to conventional chemical etchants. Dry processes (gray-shaded area), despite their environmental advantages, remain underutilized. Plasma treatment is used in ∼4% of the studies, and laser dotting is used in only ∼1% of the studies. This gap reflects the technical and economic barriers that often limit the adoption of the dry methods.^34^ Overcoming these bottlenecks in textiles requires a combination of technological, process-engineering, and strategic measures. Shifting from vacuum-based to atmospheric-pressure nonthermal plasma (e.g., dielectric-barrier discharge or plasma-jet systems) eliminates the need for costly vacuum infrastructure and enables true roll-to-roll, in-line processing.^34^ Uniform, large-area treatment can be achieved by employing multinozzle or wide-electrode designs that create a continuous plasma curtain across the whole web width. Extending the plasma path through sequential modules or longer tunnels increases the deposited dose without slowing line speed.^34,227^ To mitigate shadowing and improve penetration into thick or highly twisted fabrics, indirect or after-glow configurations and dual-dielectric DBD setups suppress filamentation and allow reactive species to reach hidden surfaces. Recycling carrier gases (especially helium or nitrogen) and using nitrogen-dominant atmospheres reduce operating expenses, and modular pilot lines allow rapid recipe optimization and staff training before full-scale deployment. This improvements have already been proven at lab-to-pilot scale. Targeting high-value niche applications first—such as medical-grade or protective textiles—helps justify the initial capital outlay, after which standardized processes can be expanded to broader product lines.^181^ The limited use of laser-based methods arises primarily from the high cost of laser texturing systems, especially those employing femtosecond or picosecond lasers required to generate the precise nanoroughness needed for superhydrophobic surfaces. Current research in laser texturing is largely focused on rigid substrates, where controlled material removal and patterning produce well-defined results. In contrast, cellulosic textiles present additional challenges: laser processing involves localized, intense heating, and identifying the optimal processing window—in terms of fluence, pulse duration, and spot size—to create the desired surface morphology without inducing thermal degradation or damaging delicate micro/nanofiber structures remains a complex and time-consuming research task.^228,229^ Although scale-up is possible, it remains limited and is not compatible with roll-to-roll processing. Another bottleneck is that laser ablation becomes time-consuming at larger scales as seen from our evaluation. Layer-by-layer (LbL) self-assembly and spin coating were not reported between 2022 and 2024, which may be related to the challenges associated with their industrial implementation.

**Figure 19:**
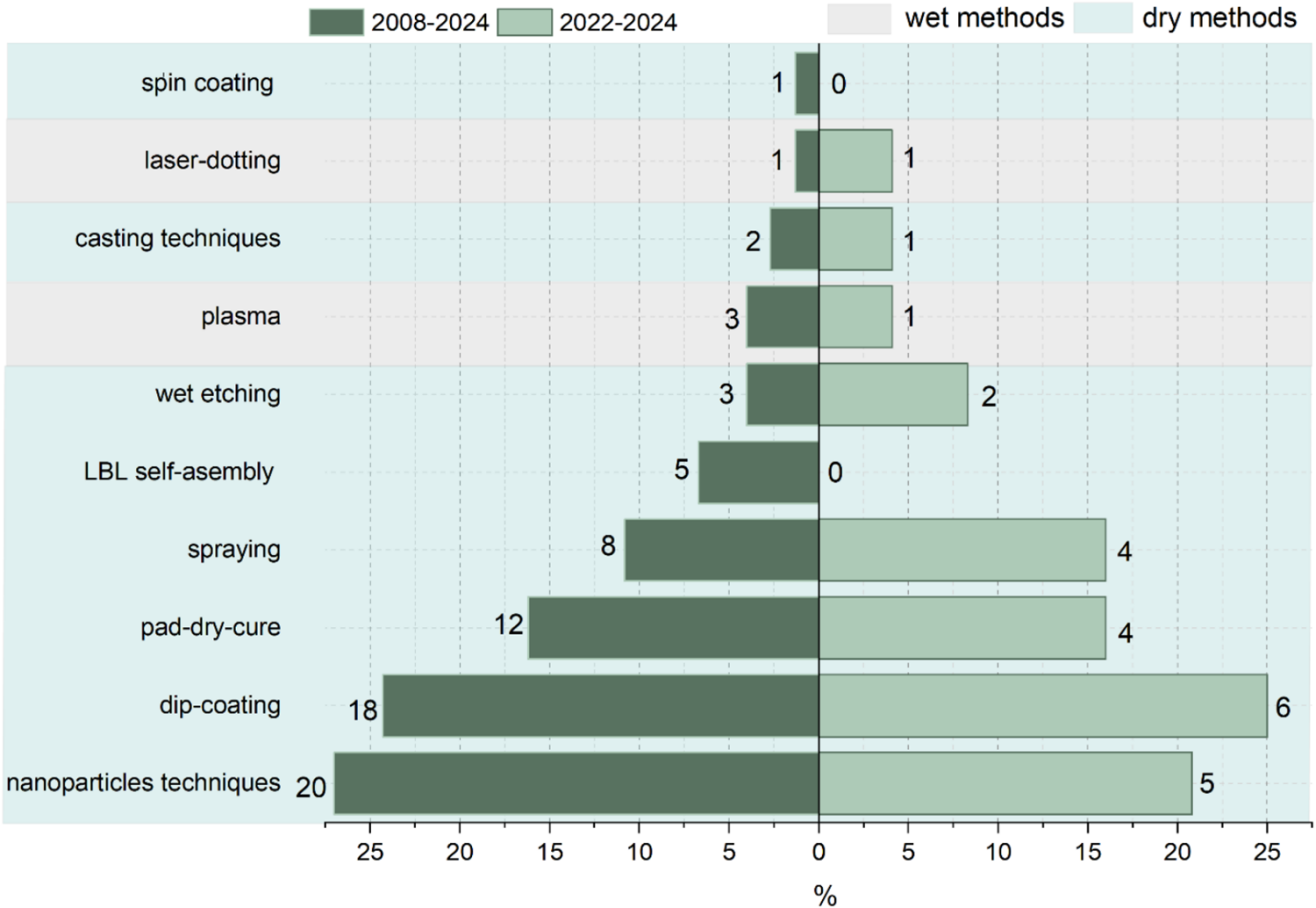
Percentage frequency and corresponding number of publications (values above bars) for each method applied to achieve hydrophobic effects on natural textiles, based on studies published from 2008–2024, highlighting the most recent developments between 2022–2024.

## 7 Conclusion and Future Perspectives

This review systematically evaluated PFAS-free approaches for enhancing (super)­hydrophobicity of natural fibers, providing an integrated environmental and industrial feasibility assessment as a guideline for sustainable textile hydrophobization based on gate-to gate criteria. The results demonstrate that while numerous naturally derived coatings can achieve excellent water repellency, overall sustainability outcomes are also strongly governed by process design.

Among the assessed methods, spray coating emerged as the most sustainable near-term solution, offering low environmental impact and strong industrial compatibility. Pad–dry–cure remains practical due to established infrastructure, whereas plasma and laser techniques offer environmental and functional benefits but are constrained by high capital costs and scale-up challenges. Laboratory-scale methods such as spin coating, solvent casting, drop casting, and layer-by-layer assembly offer excellent process control but are not suitable for large-scale production and are unlikely to be directly applicable to industrial textile manufacturing without substantial process redesign. Frequency analysis shows that wet chemical techniques dominate (>90%) due to operational simplicity, compatibility with existing lines, lower initial investment, and the hydrophilic nature of biobased fibers. However, reliance on wet processing poses sustainability challenges, including high water and solvent use, effluent generation, and chemical management, whereas dry technologies, despite lower environmental burdens, remain limited by scalability, capital, and process integration constraints.

Progress toward sustainable textile hydrophobization will depend on natural-based formulations and improvements in their durability, alongside hybrid processing strategies that integrate wet and dry methodologies to balance performance, scalability, and environmental responsibility. Plasma-enabled surface activation, combined with biobased wet coatings, can enhance interfacial adhesion and durability while reducing reliance on synthetic binders. Likewise, spray- or pad–dry–cure application of natural or sol–gel–derived coatings can be enhanced through localized, precision deposition techniques that minimize material inputs and waste generation. Such hybrid and sequential approaches provide viable pathways toward durable, PFAS-free, and circular textile finishing systems. Future research should prioritize standardized sustainability metrics that capture full life-cycle impacts. Evaluating circularity—particularly coating recoverability, recyclability of treated textiles, safe degradation pathways, and long-term retention of hydrophobic performance—will be essential for closing material loops within textile value chains. Advancing scalable and modular equipment for dry and semidry technologies that enable continuous roll-to-roll processing with reduced capital and energy demands will further accelerate industrial adoption. Future possibilities may also rely on advanced, yet still underexplored in combination with natural textiles, low environmental impact technologies such as electrospinning, UV-assisted digital finishing, supercritical CO_2_ processing, and foam-based applications. Concurrently, the development of multifunctional biobased coatings with self-healing, antimicrobial, UV-protective, and durable water-repellent properties can extend the service life of textiles and reduce resource consumption throughout their lifecycle.

The most viable near-term pathway toward sustainable and circular hydrophobic textile finishing lies in optimizing scalable coating platforms while advancing green chemistry, resource-efficient processing, and closed-loop manufacturing strategies. Continued technological maturation, supported by mechanistic insights from laboratory-scale research, will drive the next generation of environmentally responsible textile functionalization.

## Abbreviations

AKD: alkyl ketene dimer
APGD: atmospheric pressure glow discharge
BTCA: butanetetracarboxylic acid
CAPEX: capital expenses
CESO: cured epoxidized soybean oil
CNC: cellulose nanocrystal
CNF: cellulose nanofibers
CO: self-assembly; coordination self-assembly
CS: chitosan
CTAB: cetyltrimethyl ammonium bromide
DES: deep eutectic solvent
DTSAC: dimethyloctadecyl­[3-(trimethoxysilyl)­propyl]­ammonium chloride
ECHA: European Chemicals Agency
EHS: environmental, health, and safety
ESO: epoxidized soybean oil
FTIR: Fourier Transform Infrared Spectroscopy
GOTS: Global Organic Textile Standard
GWP: green warming potential
HDTS: hexadecyltrichlorosilane
HMTA: hexamethylenetetramine
LCA: life cycle assessment
MPTES: 3-mercaptopropyltriethoxysilane
MTMS: methyltrimethoxysilane
MRSL: Manufacturing Restricted Substances List
NPS: nanoparticles
ODA: octadecylamine
OPEX: operational expenses
PA: phytic acid
PAni: polyaniline
PDC: pad-dry-cure
PDMS: polydimethylsiloxane
PFAS: polyfluoroalkyl substances
PLL: poly-l-lysine
PSS: poly­(sodium-4-styrenesulfonate)
PTL: phase-transited lysozyme
REACH: Registration, Evaluation, Authorization, and Restriction of Chemicals
SA: sliding angle
STA: stearic acid
SVHC: substance of very high concern
TA: tannic acid
TEMPO: 2,2,6,6-tetramethylpiperidine-1-oxyl
TEOS: tetraethyl orthosilicate/hydrochloric acid
TIP/DEA: isopropoxide/diethanolamine
TPC: terephthaloyl chloride
TSCA: toxic Substances Control Act
VOCs: volatile organic compounds
VTMS: vinyltrimethoxysilane
WCA: water contact angle
ZDHC: Zero Discharge of Hazardous Chemicals
